# Author Correction: Prospective de novo drug design with deep interactome learning

**DOI:** 10.1038/s41467-025-55964-1

**Published:** 2025-01-17

**Authors:** Kenneth Atz, Leandro Cotos, Clemens Isert, Maria Håkansson, Dorota Focht, Mattis Hilleke, David F. Nippa, Michael Iff, Jann Ledergerber, Carl C. G. Schiebroek, Valentina Romeo, Jan A. Hiss, Daniel Merk, Petra Schneider, Bernd Kuhn, Uwe Grether, Gisbert Schneider

**Affiliations:** 1 ETH Zurich, Department of Chemistry and Applied Biosciences, Vladimir-Prelog-Weg 4, 8093 Zurich, Switzerland; 2 SARomics Biostructures AB, Medicon Village, SE-223 81 Lund, Sweden; 3 Roche Pharma Research and Early Development (pRED), Roche Innovation Center Basel, F. Hoffmann-La Roche Ltd., Grenzacherstrasse 124, CH-4070 Basel, Switzerland; 4 Department of Pharmacy, Ludwig-Maximilians-Universität München, Butenandtstrasse 5, 81377 Munich, Germany

**Keywords:** Structure-based drug design, Machine learning, Computational chemistry, Medicinal chemistry

Correction to: *Nature Communications* 10.1038/s41467-024-47613-w, published online 22 April 2024

The original version of this article contained an error in panel b of Fig. 3, in which the structure of compound 9 on the right side was incorrectly labelled as 6. The corrected version replaces the label 6 with 9. The description of panel b in the legend of Figure 3 was corrected to reflect this change and to clarify the numeric ordering of the structures. The original version stated: ‘**b** Molecular structures of the five top-ranking de novo designs. The ranking criteria were PPARγ  +  PPARδ dual-target affinity and structural novelty (left, **1** & **6**–**9**), or PPARγ single-target affinity and structural novelty (right, **2**, **6**, & **10**–**12**).’ The corrected version states “**b** Molecular structures of the top-ranking de novo designs. The predicted quality of the molecules decreases progressively from top to bottom. The ranking criteria were PPARγ  +  PPARδ dual-target affinity and structural novelty (left, **1** & **6–9**), or PPARγ single-target affinity and structural novelty (right, **2**, & **9–12**). Molecule **9** ranks among the top five molecules on both lists.” This has been corrected in both the PDF and HTML versions of the article.

