# Supplementary material for: Prospective de novo drug design with deep interactome learning
Source: Nat Commun. 2024 Apr 22;15:3408. doi: 10.1038/s41467-024-47613-w (PMC11035696; doi:10.1038/s41467-024-47613-w)
Supplement: Supplementary file 1 — Supplementary Information [file 41467_2024_47613_MOESM1_ESM.pdf]

Supplementary Information:
Prospective de novo drug design with deep interactome learning

Kenneth Atz[1], Leandro Cotos[1], Clemens Isert[1], Maria Håkansson[2], Dorota Focht[2], Mattis Hilleke[1], David F. Nippa[3,4], Michael Iff[1], Jann Ledergerber[1], Carl C. G. Schiebroek[1], Valentina Romeo[3], Jan A. Hiss[1], Daniel Merk[4], Petra Schneider[1], Bernd Kuhn[3], Uwe Grether[3] & Gisbert Schneider[1,*]

[1]ETH Zurich, Department of Chemistry and Applied Sciences, Vladimir-Prelog-Weg 4, 8093 Zurich, Switzerland.
[2]Roche Pharma Research and Early Development (pRED), Roche Innovation Center Basel, F. Hoffmann-La Roche Ltd., Grenzacherstrasse 124, CH-4070 Basel, Switzerland.
[3]Department of Pharmacy, Ludwig-Maximilians-Universität München, Butenandtstrasse 5, 81377 Munich, Germany.
[4]SARomics Biostructures, Scheelevägen 2, SE-223 81 Lund, Sweden.
* To whom correspondence should be addressed. E-mail: gisbert@ethz.ch

Contents

SI1 Neural Network Training **2**

SI2 Molecular Library Evaluation **2**
SI2.1 Novel Chemical Entities 2
SI2.2 Quantitative Structure-Activity Relationship (QSAR) 3
SI2.3 Retrosynthetic Accessibility 7
SI2.4 Molecular Novelty 8
SI2.5 Molecular Diversity 9

SI3 Amino Acid B-factor **9**

SI4 Data Set Preprossessing **9**

SI5 Chemical Alphabet **10**

SI6 Comparison of Different Dragonfly Models **11**
SI6.1 General Molecular Properties 11
SI6.2 Physicochemical Properties 13
SI6.3 Synthetic Ligand Templates for de novo Design 15
SI6.4 Additional Results on Ligand-based de novo Design 16

SI7 Training Data Set Extrapolation **19**

SI8 Amino Acid Sequence Identity **20**

SI9 Biological Characterization **21**

SI10 Protein-Ligand Co-Crystallization **24**
SI10.1 Constructs Details 24
SI10.2 Large-Scale Expression of PPARγ in *E. coli* BL-21(DE3) cells 24
SI10.3 Purification of PPARγrotein 24
SI10.4 Crystallization 24
SI10.5 Freezing 25
SI10.6 Data Collection 25
SI10.7 Structure Determination and Refinement 26
SI10.8 PPARγ Co-Crystal Structure Complex 26

SI11 Absorption Distribution Metabolism and Excretion and Toxicity Data **27**

SI12 Absolute Free Binding Energy Calculations **29**

SI13 Chemical Synthesis **30**
SI13.1 Experimental Procedures and Analytical Data 30
SI13.2 Reagent and purification information 30
SI13.3 Analytical information 30
SI13.4 Experimental procedures and analytical data 31
SI13.5 NMR Spectra 51

SI1 Neural Network Training

Neural network training was conducted using PyTorch Geometric (version 2.0.2)[1] and PyTorch (version 1.10.1+cu102)[2]. The training process occurred on a GPU, specifically the NVIDIA Tesla V100-SXM2 32 GB, for a total duration of 120 hours. A batch size of 128 was used for ligand-based design, while a batch size of 48 was employed for structure-based design. The optimization of the neural networks was performed using the Adam stochastic gradient descent optimizer [3], with a learning rate set at 0.0005. Cross-entropy loss was utilized as the loss function, and a decay factor of 0.5 was applied after every 10 epochs. Additionally, an exponential smoothing factor of 0.7 was incorporated into the training process. All the models used in this study were trained on the Euler computing cluster at ETH Zurich, Switzerland.

SI2 Molecular Library Evaluation

To assess the quality of molecular libraries generated by Dragonfly, several critical properties of these generated molecules come into play. Specifically, we focus on five key molecular characteristics: (i) number of novel chemical entities, (ii) predicted bioactivity, (iii) predicted synthesizability, (iv) structural and scaffold novelty, and (v) diversity. These five criteria served as the foundation for evaluating the molecular libraries produced by Dragonfly. We employed them to investigate Dragonfly's performance in ligand- and structure-based design using different molecular representations, such as SMILES-strings and self-referencing embedded strings [SELFIES]. This evaluation is detailed in Section SI6.

SI2.1 Novel Chemical Entities

In the realm of de novo molecular design using chemical language models (CLMs), the process involves generating strings when applying a trained CLM. However, it's crucial to note that the number of sampled strings doesn't necessarily equate to the number of novel chemical entities (NCEs) produced by the model. As a result, the standard practice in evaluating the quality of molecules generated by CLMs involves considering the fraction of valid, unique, and novel molecules they generate for each sampled string [4–6]. The three terms are described as following: validity, *i.e.*, removing strings that do not transform into a valid molecule; uniqueness, *i.e.*, removing duplicates, and novelty, *i.e.*, removing molecules that are in the training data set.

SI2.2 Quantitative Structure-Activity Relationship (QSAR)

In total, 1265 QSAR models were trained for each of the three descriptors. This number corresponds to each individual target from the ChEMBL database [7] (Version 29) that had at least 200 annotated molecules with bioaffinities available. The QSAR models were trained on datasets ranging from 200 to over 10'000 molecules, with an average of 1056 molecules per QSAR model (as depicted in Figure S2). When evaluating the performance of these models, it was found that the ECFP models consistently achieved the lowest test set mean absolute error (MAE), with an average MAE of 0.533 (±0.139) $\mathrm{p}K_{\mathrm{I}}$. In comparison, the chemical advanced template search (CATS) and ultra-fast shape recognition with atom types (USRCAT) models exhibited slightly higher errors, averaging 0.725(±0.226) and 0.790(± 0.247), respectively (as illustrated in Figure S2).

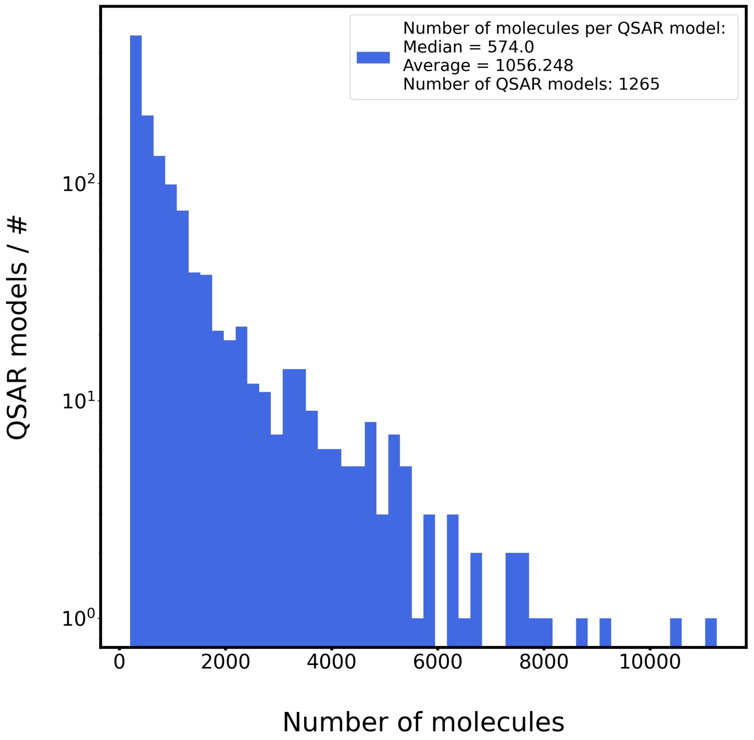

Figure S1: Histogram illustrating the number of molecules for each of the 1265 quantitative structure-activity relationship (QSAR) models. Median number of molecules per target: 574; Average number of molecules per target: 1056; Number of QSAR targets: 1265. Source data are provided as a Source Data file.

To assess the performance of the ligand-based QSAR models, mean absolute errors (MAE) were computed on the test sets. These errors were calculated across a range of training set sizes using a 10-fold cross-validation approach. In this setup, each model was independently trained ten times on different random splits of test and validation sets, and subsequently tested on the same fixed test set. It is worth noting that the resulting error term exhibits an inverse relationship with the number of training points in the training set, a phenomenon well-documented in the literature [8]. This relationship leads to linear-like curves when plotted on a log-log scale. In Figure S3, you can observe the learning curves for 24 known targets, each using one of the three investigated descriptors (ECFP, CATS, USRCAT).

Additionally, we conducted a comparison between the developed Kernel Ridge Regression (KRR) models and two decision tree-based baseline models, namely gradient boosting and extreme gradient boosting (XGBoost). Across the majority of the 1161 investigated targets, the KRR models consistently demonstrated higher accuracy when compared to the two decision tree baselines. This comparison is visually depicted in Figure S4 and Figure S5. These 1161 targets represent a subset of the total 1265 QSAR models. Specifically, this subset was selected

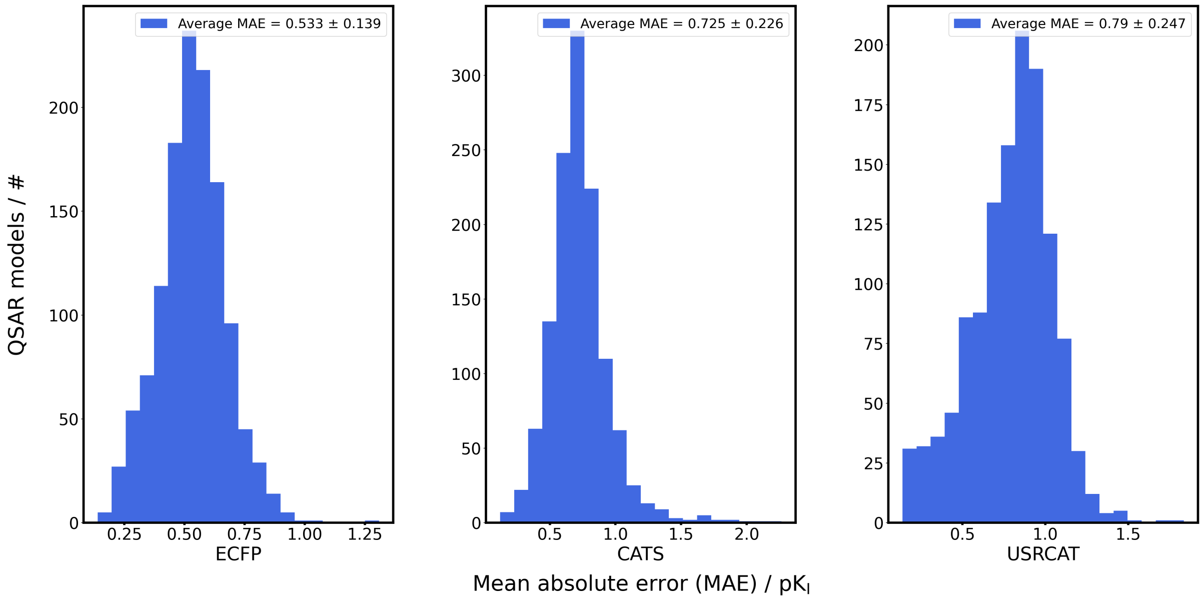

Figure S2: Histogram illustrating the test set errors for the 1265 QSAR models and the three descriptors. Source data are provided as a Source Data file.

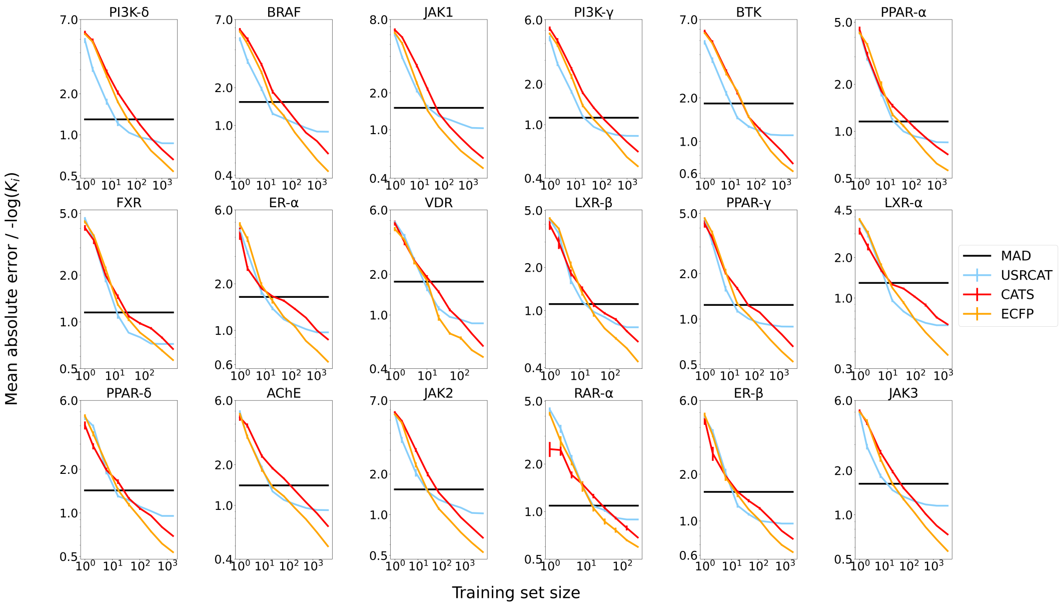

Figure S3: Learning curves for 18 selected drug targets showing the prediction error of the ligand-based QSAR models for three descriptors (ECFP, CATS and USRCAT). Each plot is scaled logarithmically on both axes, the mean absolute deviation (MAD) of the training data set is shown on each plot as a black line, and for all targets, the human proteins are selected. The error bars indicate the standard deviation observed in a 10-fold cross-validation. Source data are provided as a Source Data file.

to include all targets with up to 3000 annotated bioactivities, which enabled efficient hyperparameter screening for the comparison between the models.

The two decision tree algorithms were optimized using the following hyperparameters:

- **XGBoost**: The XGBoost algorithm (XGBoost Python Package version 1.6.2 [9]) was optimized by fine-tuning the following hyperparameters: n_estimators=[1, 2, 5, 10], reg_lambda=[0.01, 0.05, 0.1, 0.5, 1], eta=[0.01, 0.05, 0.1, 0.5, 1], gamma=[0.01, 0.05, 0.1, 0.5, 1], and max_depth=[1, 2, 4, 5].
- **Gradient boosting**: The gradient boosting algorithm (GradientBoostingClassifier and GradientBoostingRegressor by Sklearn version 0.23.2 [10]) was optimized by fine-tuning the following hyperparameters:

n_estimators=[1, 2, 5, 10], learning_rate=[0.01, 0.05, 0.1, 0.5, 1], and max_depth=[1, 2, 4, 5].

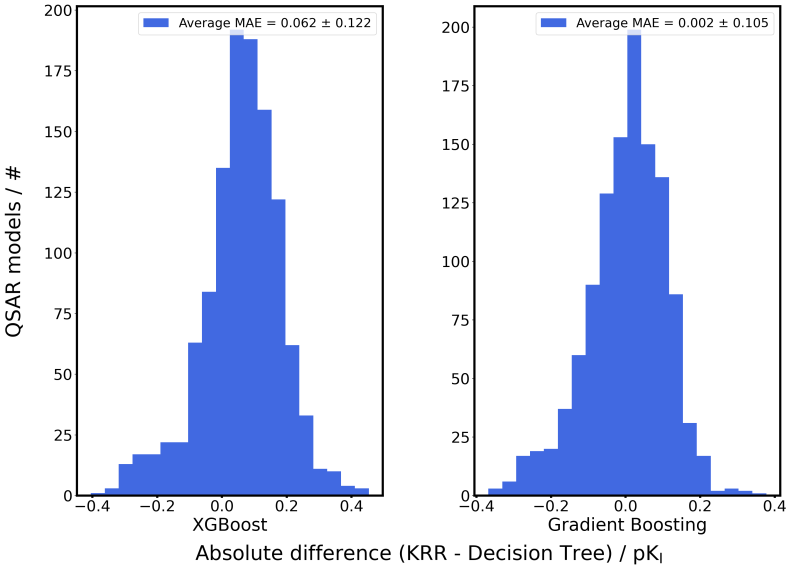

Figure S4: Absolute performance difference (Kernel ridge regression (KRR) - Decision Tree) for each of the 1265 targets. Positive numbers indicate better performance of KRR models. Source data are provided as a Source Data file.

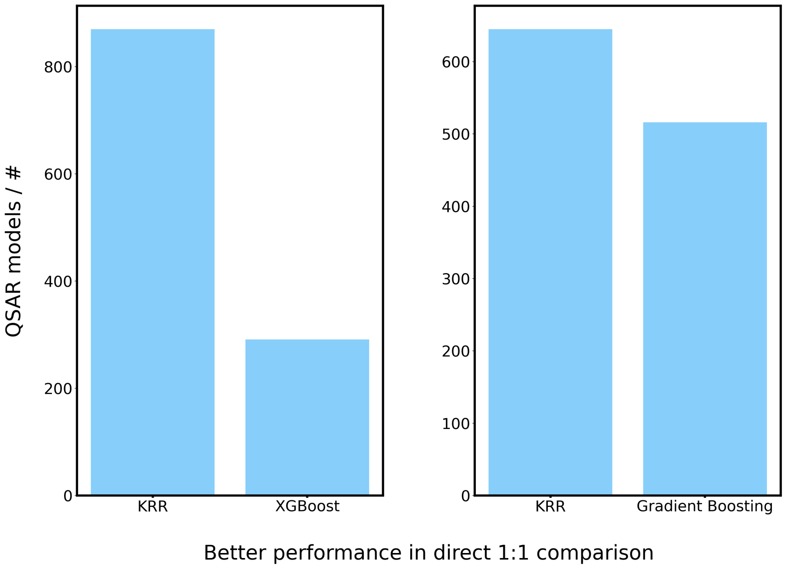

Figure S5: Direct comparison of Decision Tree and KRR models. The bar indicate the number of targets for which the individual models achieve better performance. Source data are provided as a Source Data file.

SI2.3 Retrosynthetic Accessibility

The retrosynthetic accessibility score (RAScore) [11] was integrated into the workflow to estimate the synthesizability of the generated molecules. RAScore is a validated synthesizability prediction model based on AiZynthFinder[12] and was trained to predict if AiZynthFinder [12] (*i.e.*, a rule-based retrosynthesis method that yields a binary output) finds a path for a given molecule. RAScore uses an ECFP bit-vector (radius = 3, dimension = 2048) as input and outputs a value between 0 (not synthetically accessible) and 1 (synthetically accessible). RAScore has shown to accurately reflect the binary output of AiZynthFinder by being almost 10^4 times faster. [11] The RAScore values for the molecules of the training data set show (Figure S6 for details) (i) that most of the molecules are predicted to be synthesizable (*i.e.*, $> 90\%$ are predicted with a RAScore ≥ 0.5), and (ii) that most of the values are very close to 0 or to 1. The latter is expected since RAScore was trained on binary values. [11]
Figure S6 illustrates the distribution of retrosynthetic accessibility scores (RAScoces) for the 360k molecules used within the drug-target interactome.

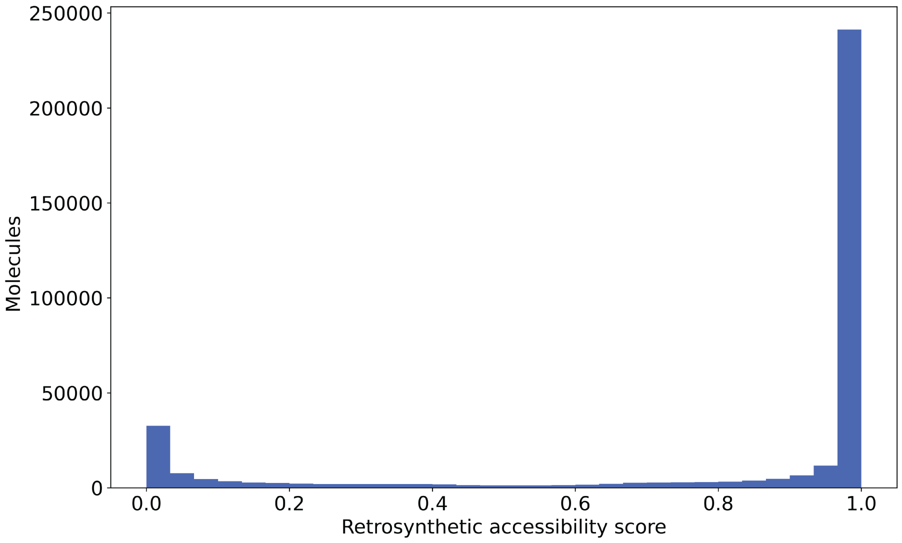

Figure S6: Histogram visualizing the calculated retrosynthetic accessibility scores (RAScoce) for the molecules in the training data set. Source data are provided as a Source Data file.

SI2.4 Molecular Novelty

Next to desired biologic activity (Section SI2.2 for details) and practical retrosynthetic accessibility (Section SI2.3 for details), the novelty of de novo designed molecules is indispensable for the designs to be useful in modern drug discovery to circumvent patented chemical space. [6] Recently reported bioactive small molecules with desired synthetic accessibility and *in vivo* profile have only shown incremental changes to known compounds, that were used to train the deep learning model. [13] Consequently, standards regarding the aspect of structural novelty of novel molecules designed by an AI have become central to de novo drug design. [14] Whereas the novelty criteria that are applied in our study are described in the main part of the paper, Figure S7 illustrates the two central scaffolds used for the novelty score, *i.e.*, carbon and Murcko [15] scaffolds.

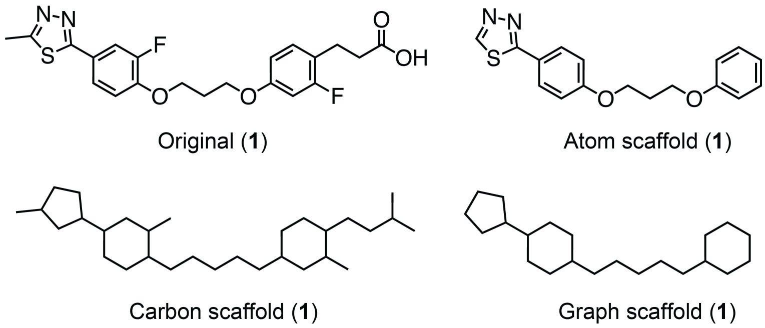

Figure S7: Atom, carbon, atom (*i.e.*, Murcko) and graph scaffolds are visualized for the de novo design **1**.

Figure S8 illustrates the closest structures in ChEMBl for the two de novo designs **1** and **2**.

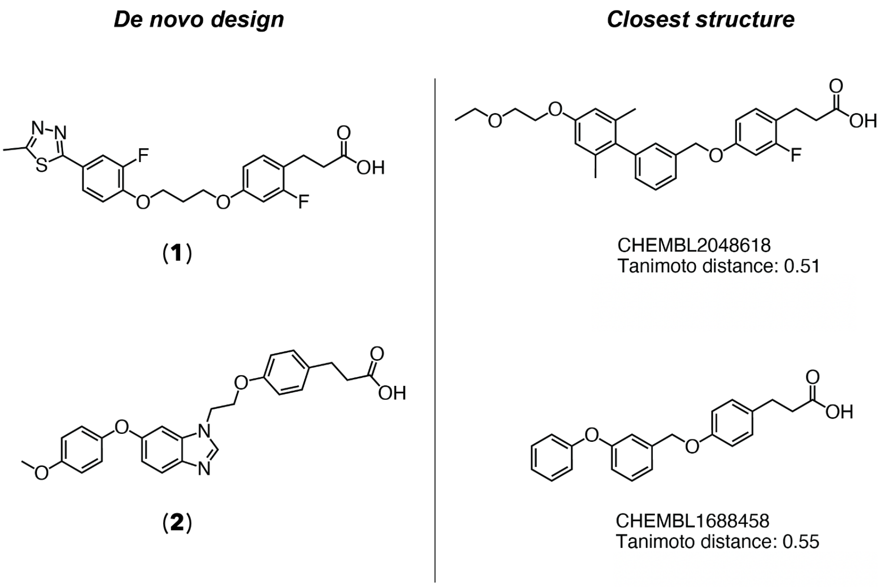

Figure S8: The two closest structures in ChEMBl for the two de novo designs **1** and **2**.

SI2.5 Molecular Diversity

Molecular diversity describes structural differences among the generated molecules with each other, whereas molecular novelty describes structural differences between the generated molecules and known molecules in the training set. Molecular diversity is quantified by structural and scaffold diversity.

- *Structural diversity* is defined by two values: (i) By the average Jaccard distance from a each generated molecule to all other molecules generated by the model (*i.e.*, Diversity I). (ii) By the average fraction of molecules with a Jaccard distance > 0.5 for every molecules in the generated set (*i.e.*, Diversity II). A Jaccard distance > 0.5 means that the two molecules share more than 50% of their structural features (*i.e.*, the two molecules are highly similar to each other). For both diversity values higher numbers translate to more diversity within the generated molecules.
- *Scaffold diversity* is quantified by the number of unique and novel atom and carbon scaffolds, yielding four values.

SI3 Amino Acid B-factor

The B-factor of amino acids in protein structures describes the flexibility of the individual side chains in the folded protein. [16, 17] The B-factor has been calculated for the side chains of the proteins in the training data set and has been added as additional node feature.

SI4 Data Set Preprossessing

The following software was used for the preprocessing of ChEMBL and PDBBind:

- MySQL (Version 8.0.28): Reference 18
- ChEMBL29 database: Reference 7
- ChEMBL Structure Pipeline package: Reference 19
- PDBBind (Version 2020): Reference 20 and 21
- UNIPROT: Reference 22
- data set covering allosteric modulation: Reference 23

SI5 Chemical Alphabet

Table S1 describes the number of characters for the two alphabets, *i.e.*, SMILES-strings [24] and self-referencing embedded strings (SELFIES) [25]. The individual characters Ω_{SMILES} and Ω_{SELFIES} and are described below.

Table S1: Key numbers for the two alphabets generated for SMILES-strings and SELFIES.

	**SMILES**	**SELFIES**
Number or characters	57	85
Padded string length	110	130

Ω_{SMILES} = #, (,), −, /, 1, 2, 3, 4, 5, 6, 7, 8, 9, =, C, F, I, N, O, P, S, \\, c, n, o, s, Cl, [nH], Br, [N+](=O)[O−], O=[N+][O−], [O−][N+](=O), S(=O)(=O), [C@@H], [C@H], [C@@], [C@], [N−]=[N+]=N, [O−], [n+], [n−], [N+], [N−], [s+], [S−], [S+], [S@@], [S@], =O, O=, C#N, N#C, [H], [C−], x, y, z.

Ω_{SELFIES} = [#Branch1], [#Branch2], [#C−1], [#C], [#N+1], [#N],[#Ring2], [−/Ring1], [−/Ring2], [\\Ring1], [−\\Ring2], [/Br], [/C@@H1], [/C@@], [/C@H1], [/C@], [/C], [/Cl], [/F],[/I], [/N+1], [/NH1], [/N], [/O−1], [/O], [/S+1], [/S],[=Branch1], [=Branch2], [=C], [=N+1], [=N−1],[=N], [=O], [=Ring1], [=Ring2], [=S+1], [=S@@], [=S@],[=S], [Br], [Branch1], [Branch2], [C−1], [C@@H1], [C@@],[C@H1], [C@], [C], [Cl], [F], [I], [N+1], [N−1], [NH1],[N], [O−1], [O], [P], [Ring1], [Ring2], [S+1], [S−1], [S@@], [S@], [S], [\\Br], [\\C@@H1], [\\C@@], [\\C@H1],[\\C@], [\\C], [\\Cl], [\\F], [\\I], [\\N+1], [\\NH1], [\\N],[\\O−1], [\\O], [\\S+1], [\\S], [\\X], [\\Y], [\\Z].

SI6 Comparison of Different Dragonfly Models

The performance of the Dragonfly models was measured by three different benchmark experiments discussed in the following sections. Section SI6 discusses the comparison of Dragonfly trained within four different frameworks, namely ligand- and structure-based design for SMILES-strings and SELFIES. Moreover, the comparison between Dragonfly and a fine-tuning-based recurrent neural network (RNN) with a long-short term memory (LSTM) cell architecture is discussed for the application to library design for 20 ligand templates with different targets. Section SI6.2 discusses the incorporation of physical and chemical properties into Dragonfly applications. Dragonfly was applied in all the discussed scenarios without any additional fine-tuning and without any knowledge of the studied templates.

For the RNN baseline, a model pre-trained on ChEMBL24 [26] was fine-tuned using individual templates with standard settings. These settings comprised fine-tuning for up to 40 epochs, a learning rate of 10^{-4}, and compound generation with a temperature of $T = 0.7$. Four different numbers of fine-tuning epochs were compared: 10, 20, 30, and 40. Notably, 30 fine-tuning epochs yielded the best trade-off between molecules with desired novelty and desired properties. Consequently, 30 fine-tuning epochs were chosen for the subsequent comparison.

SI6.1 General Molecular Properties

There are three trends that can be observed when sampling molecules with different temperature values (Figure S9). (i) Lower temperature yields more molecules with higher synthesizability, predicted bioactivity and validity. (ii) Higher temperature yields greater diversity. (iii) Since validity decreases with increasing temperature values, and uniqueness increases with decreasing temperature values, the resulting molecules that are valid and unique are derived from a temperature T=0.5 which was found to be the best trade-off for de novo design with Dragonfly regarding the two diverging trends. The same trend has been observed for all four Dragonfly set-ups. Figure S9 illustrates the observed trends during training for ligand-based design using SMILES-strings.

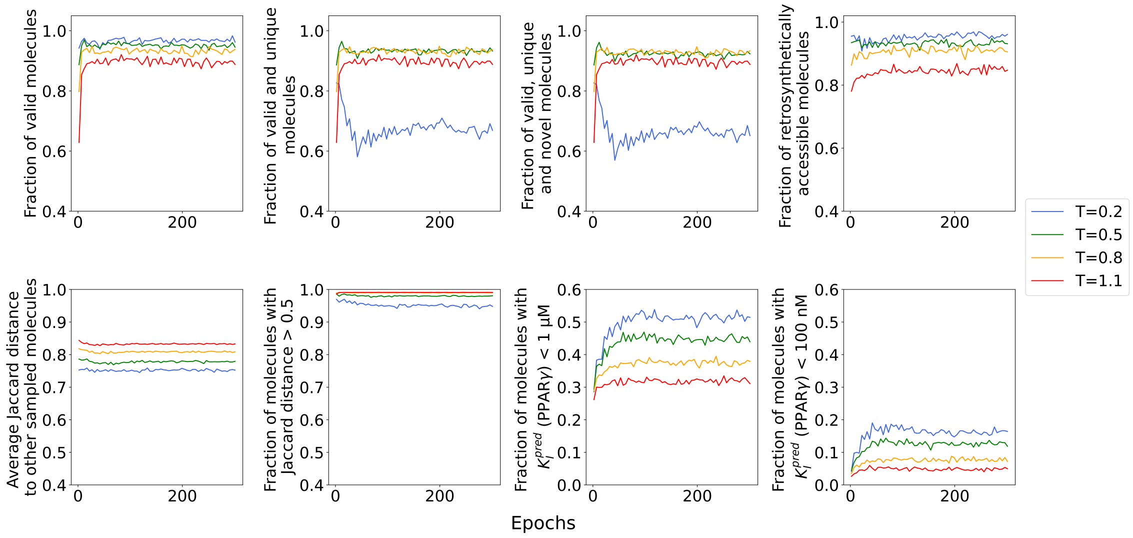

Figure S9: Eight properties are plotted over all epochs during neural network training. 2500 molecules are sampled at each epoch for each temperature value. The plot has been smoothed by visualizing every fifth point as a rolling average to enable better visibility. Dragonfly model: Ligand-based design using SMILES-strings. Source data are provided as a Source Data file.

Sampling molecules at different temperature values has shown that the number of unique and novel scaffolds increases with sampling temperature. The same trend has been observed for all four Dragonfly set-ups. Figure S10 illustrates the observed trends during training for ligand-based design using SMILES-strings.

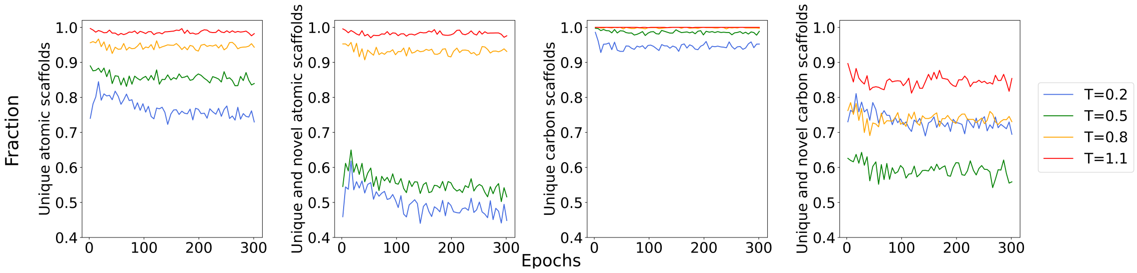

Figure S10: Scaffold properties plotted over all epochs during neural network training. 2500 molecules were sampled at each epoch for each temperature value. The plot has been smoothed by visualizing every fifth point as rolling average to enable better visibility. DRAGONFLY model: Ligand-based design using SMILES-strings. Source data are provided as a Source Data file.

SI6.2 Physicochemical Properties

Figure S11 illustrates the translation of desired properties for the first five generated molecules when sampling from the PPARγ crystal structure as input. It can be seen that the desired amount of hydrogen bond donors (1, 0 and 3) are found in all sampled molecules.

Sampling molecules with different temperature values has yielded trends such as shown in Figure S12 (ligand-based design, SMILES-strings), where molecules sampled with lower temperature more accurately reflect desired chemical and physical properties.

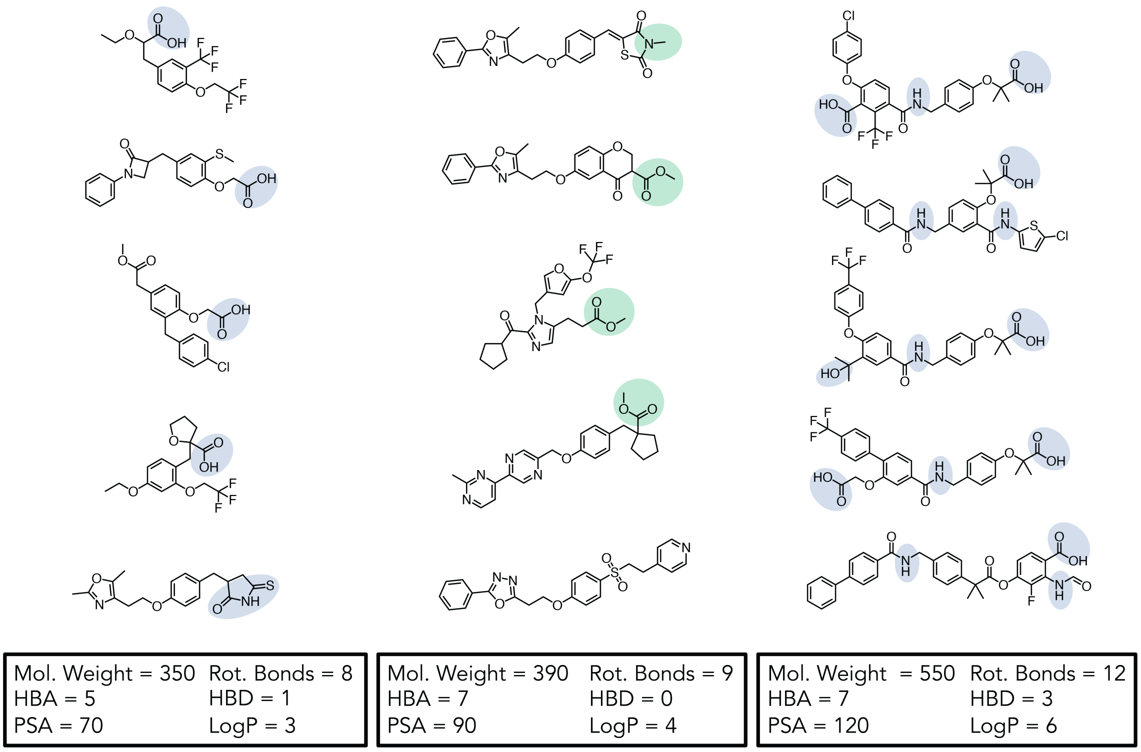

Figure S11: The first five molecules for three different sets of molecular properties sampled from the PPARγ crystal structure (PDB-ID 3G9E [27]) are depicted (**121 - 135**). Special focus is placed on a different number of hydrogen bond donors (1, 0 and 3), which translate to all the generated molecules. The hydrogen bond donors are visualized on the molecules in light blue. For the case where 0 hydrogen bond donors per molecule are generated (center), the moieties which could allow for hydrogen bond donors by exchanging a methyl group with a hydrogen are highlighted in green. Source data are provided as a Source Data file.

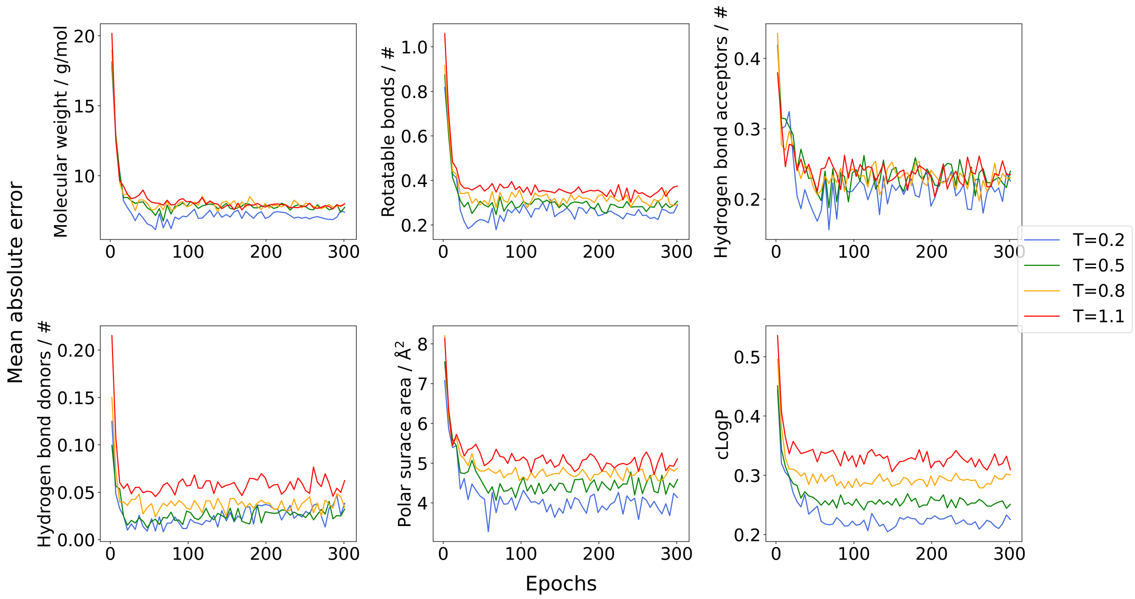

Figure S12: Physico-chemical properties plotted over all epochs during neural network training. 2500 molecules are sampled at each epoch for each temperature value. The plot has been smoothed by visualizing every fifth point as a rolling average to enable better visibility. DRAGONFLY model: Ligand-based design, SMILES-strings. Source data are provided as a Source Data file.

SI6.3 Synthetic Ligand Templates for de novo Design

Table S2: Mapping of drug target to selected ChEMBL ids of the small-molecule ligands. JAK = Janus kinase; PPAR = Peroxisome proliferator-activated receptor; BRAF = Serine/threonine-protein kinase B-Raf (rapidly accelerated fibrosarcoma); ABL = Tyrosine-protein kinase ABL1; BTK = Bruton's tyrosine kinase; PI3K = Phosphoinositide 3-kinases.

**Target**	**ChEMBL ids**
JAK1	CHEMBL2159207, CHEMBL3903172, CHEMBL3911395, CHEMBL3944525, CHEMBL3647748
JAK2	CHEMBL2381979, CHEMBL3912167, CHEMBL3647725, CHEMBL3797902, CHEMBL570686
JAK3	CHEMBL3589169, CHEMBL392998, CHEMBL3937204, CHEMBL3930342, CHEMBL3622144
PPARα	CHEMBL374090, CHEMBL220152, CHEMBL218784, CHEMBL219626, CHEMBL384203
PPARδ	CHEMBL411045, CHEMBL1081601, CHEMBL4592038, CHEMBL220508, CHEMBL1081764
PPARγ	CHEMBL519504, CHEMBL50885, CHEMBL399310, CHEMBL3976442, CHEMBL121
BRAF	CHEMBL3948705, CHEMBL3952726, CHEMBL3639601, CHEMBL3694189, CHEMBL3661251
ABL	CHEMBL3685175, CHEMBL3689545, CHEMBL1336, CHEMBL1171837, CHEMBL459850
BTK	CHEMBL3908775, CHEMBL4214229, CHEMBL4107322, CHEMBL4096195, CHEMBL3652671
PI3Kδ	CHEMBL496941, CHEMBL4105075, CHEMBL2089114, CHEMBL3905026, CHEMBL512648

Table S3: Mapping of drug target to selected ChEMBL ids of the small-molecule ligands. PI3K = Phosphoinositide 3-kinases; RAR = Retinoic acid receptor; LXR = Liver X receptor; FXR = Farnesoid X receptor; RXR = Retinoid X receptor; ER = Estrogen receptor.

**Target**	**ChEMBL ids**
PI3Kγ	CHEMBL1213117, CHEMBL3950901, CHEMBL589345, CHEMBL592445, CHEMBL2165268
RARα	CHEMBL344973,CHEMBL550796, CHEMBL165843, CHEMBL131925, CHEMBL3954703
RARβ	CHEMBL152740, CHEMBL146636, CHEMBL512879, CHEMBL34464, CHEMBL3916016
LXRα	CHEMBL595483, CHEMBL215108,CHEMBL459472, CHEMBL516464, CHEMBL3360963
LXRβ	CHEMBL215108, CHEMBL460198, CHEMBL3673700, CHEMBL593960, CHEMBL517015
FXR	CHEMBL101327, CHEMBL4641017, CHEMBL560147, CHEMBL3746388, CHEMBL1209365
VDR	CHEMBL4202601, CHEMBL2172539, CHEMBL3747675, CHEMBL2172538, CHEMBL4437809
RXRα	CHEMBL44478, CHEMBL432140, CHEMBL247000, CHEMBL312395, CHEMBL337393
ERα	CHEMBL4455921, CHEMBL85536, CHEMBL3774647, CHEMBL4167575, CHEMBL4459248
ERβ	CHEMBL194774, CHEMBL4225690, CHEMBL195080, CHEMBL4203290, CHEMBL182404

SI6.4 Additional Results on Ligand-based de novo Design

Table S4: Results obtained for the following set up: Fine-tuned RNN using synthetic ligand templates and SMILES-strings. The numbers are presented as the mean and standard deviation, with a sample size of $N = 3$, *i.e.*, 3 Dragonfly runs, each sampling 2000 SMILES-strings.

**Template**	**Valid, unique and novel / %**	**Novelty score ≥ 0.65 / %**	**RAScore ≥ 0.5 / %**	**QSAR score ≤ 1 µM / %**	**Predicted hits / %**
**ER**α	75 (±11)	35.0 (±4.5)	59.0 (±7.1)	44.6 (±5.5)	11.5 (±1.2)
**LXR**α	91.1 (±4.9)	59.6 (±4.3)	88.0 (±4.5)	23.6 (±1.5)	8.7 (±0.4)
**RAR**α	69.7 (±5.9)	41.9 (±3.3)	57.2 (±4.3)	30.1 (±1.8)	11.1 (±0.7)
**RXR**α	60.9 (±3.8)	32.4 (±1.8)	26.5 (±1.1)	19.9 (±0.9)	1.1 (±0.0)
**ABL**	75 (±11)	22.5 (±2.9)	71.4 (±7.9)	37.7 (±4.3)	4.9 (±0.5)
**ER**β	66.8 (±4.4)	32.5 (±4.1)	50.1 (±5.7)	37.4 (±1.8)	8.8 (±0.4)
**LXR**β	92.4 (±2.5)	65.9 (±2.6)	87.9 (±2.8)	28.6 (±0.9)	11.3 (±0.4)
**RAR**β	79.0 (±1.5)	39.7 (±2.5)	70.0 (±3.7)	33.7 (±1.2)	10.6 (±0.2)
**BRAF**	89.2 (±3.5)	35.1 (±3.1)	85.9 (±3.0)	35.0 (±1.3)	6.7 (±0.3)
**BTK**	82.0 (±4.4)	64.5 (±4.1)	61.9 (±4.7)	20.7 (±1.8)	4.5 (±0.2)
**PI3K**δ	85.1 (±0.9)	66.1 (±3.4)	72.0 (±3.2)	43.6 (±1.4)	26.5 (±1.2)
**FXR**	79.9 (±1.9)	38.0 (±1.4)	72.4 (±2.2)	25.7 (±1.3)	3.6 (±0.1)
**PI3K**γ	78.2 (±6.6)	44.9 (±3.9)	73.6 (±5.5)	42.2 (±4.0)	17.0 (±1.4)
**JAK1**	52 (±12)	20.4 (±3.1)	43.1 (±5.3)	35.1 (±4.5)	6.3 (±0.8)
**JAK2**	88.8 (±3.9)	60.2 (±4.2)	79.9 (±3.4)	35.0 (±2.2)	14.5 (±0.8)
**JAK3**	75.5 (±2.0)	62.3 (±1.7)	59.2 (±2.1)	22.5 (±0.7)	7.3 (±0.3)
**PPAR**α	79.2 (±3.0)	21.5 (±1.2)	75.6 (±2.4)	20.9 (±0.8)	2.0 (±0.1)
**PPAR**δ	73.4 (±7.3)	19.6 (±1.6)	71.5 (±5.2)	40.0 (±3.2)	6.3 (±0.5)
**PPAR**γ	75.4 (±2.7)	28.7 (±1.1)	67.9 (±2.3)	29.6 (±2.4)	5.1 (±0.2)
**VDR**	53.3 (±7.4)	40.8 (±5.0)	27.2 (±5.0)	24.3 (±1.9)	6.1 (±0.5)

Table S5: Results obtained for the following set up: DRAGONFLY using synthetic ligands templates and SMILES-strings. The numbers are presented as the mean and standard deviation, with a sample size of $N = 3$, *i.e.*, 3 DRAGONFLY runs, each sampling 2000 SMILES-strings.

**Template**	**Valid, unique and novel / %**	**Novelty score ≥ 0.65 / %**	**RAScore ≥ 0.5 / %**	**QSAR score ≤ 1 μM / %**	**Predicted hits / %**
**ER**α	87.1 (±0.2)	46.2 (±0.8)	68.3 (±0.8)	56.1 (±0.7)	18.8 (±0.2)
**LXR**α	93.8 (±0.1)	79.8 (±1.5)	87.7 (±0.2)	11.4 (±0.0)	1.7 (±0.0)
**RAR**α	92.2 (±0.4)	62.4 (±0.7)	75.6 (±0.5)	32.4 (±0.7)	12.7 (±0.2)
**RXR**α	92.2 (±0.4)	62.5 (±0.4)	72.4 (±1.0)	17.1 (±0.2)	1.6 (±0.0)
**ABL**	90.4 (±0.4)	48.3 (±0.2)	85.1 (±0.5)	32.9 (±0.5)	8.4 (±0.1)
**ER**β	86.8 (±0.8)	48.7 (±0.7)	71.2 (±1.2)	53.5 (±0.5)	19.1 (±0.3)
**LXR**β	94.3 (±0.5)	80.2 (±1.2)	89.1 (±0.5)	26.2 (±0.2)	11.8 (±0.1)
**RAR**β	93.6 (±0.1)	62.4 (±1.1)	80.7 (±0.5)	33.7 (±0.5)	12.5 (±0.3)
**BRAF**	87.9 (±0.6)	46.0 (±0.8)	80.9 (±0.5)	42.9 (±0.5)	10.7 (±0.1)
**BTK**	88.9 (±0.7)	53.2 (±0.4)	69.6 (±0.9)	36.3 (±0.7)	8.8 (±0.1)
**PI3K**δ	88.0 (±0.3)	65.1 (±0.3)	76.8 (±0.3)	60.7 (±0.2)	39.7 (±0.2)
**FXR**	94.3 (±0.2)	72.0 (±1.1)	86.4 (±0.4)	22.5 (±0.1)	4.4 (±0.0)
**PI3K**γ	88.1 (±1.2)	57.6 (±1.5)	79.9 (±1.0)	43.9 (±0.8)	18.8 (±0.3)
**JAK1**	83.7 (±0.6)	36.1 (±0.5)	66.9 (±0.5)	61.5 (±0.9)	14.5 (±0.1)
**JAK2**	84.8 (±1.0)	39.4 (±0.9)	69.0 (±1.0)	55.9 (±1.5)	14.8 (±0.2)
**JAK3**	87.9 (±0.1)	48.1 (±1.5)	73.1 (±0.5)	36.6 (±0.2)	11.0 (±0.1)
**PPAR**α	92.3 (±0.3)	43.2 (±0.2)	85.4 (±0.3)	27.3 (±0.1)	4.4 (±0.0)
**PPAR**δ	91.5 (±0.2)	43.6 (±0.9)	85.8 (±0.2)	36.3 (±0.7)	10.1 (±0.1)
**PPAR**γ	91.8 (±0.3)	47.9 (±1.4)	86.0 (±0.3)	34.7 (±0.3)	9.4 (±0.0)
**VDR**	91.1 (±0.3)	57.0 (±0.5)	53.6 (±0.2)	41.3 (±0.1)	6.6 (±0.0)

Table S6: Results obtained for the following set up: DRAGONFLY using synthetic ligands templates and SELFIES. The numbers are presented as the mean and standard deviation, with a sample size of $N = 3$, *i.e.*, 3 DRAGONFLY runs, each sampling 2000 SMILES-strings.

Template	Valid, unique and novel / %	Novelty score ≥ 0.65 / %	RAScore ≥ 0.5 / %	QSAR score ≤ 1 μM / %	Predicted hits / %
**ER**α	98.5 (±0.1)	75.2 (±0.9)	72.5 (±1.1)	52.9 (±0.7)	26.3 (±0.3)
**LXR**α	99.9 (±0.1)	89.8 (±0.6)	84.6 (±0.6)	13.7 (±0.1)	1.7 (±0.0)
**RAR**α	99.8 (±0.0)	87.5 (±0.3)	77.1 (±0.2)	29.6 (±0.3)	14.0 (±0.1)
**RXR**α	99.9 (±0.0)	89.5 (±0.3)	71.5 (±0.7)	9.6 (±0.0)	0.9 (±0.0)
**ABL**	99.7 (±0.1)	77.7 (±0.5)	84.8 (±0.4)	27.6 (±0.3)	10.4 (±0.1)
**ER**β	97.4 (±0.4)	70.4 (±1.0)	75.2 (±0.8)	53.2 (±0.4)	24.9 (±0.1)
**LXR**β	100 (±0.0)	91.3 (±0.5)	84.2 (±0.3)	27.9 (±0.2)	11.1 (±0.1)
**RAR**β	99.7 (±0.1)	84.6 (±0.2)	79.7 (±0.9)	29.1 (±0.2)	9.1 (±0.0)
**BRAF**	99.7 (±0.1)	81.1 (±0.6)	77.3 (±0.4)	34.3 (±0.1)	12.4 (±0.0)
**BTK**	100 (±0.0)	85.8 (±0.7)	68.2 (±1.0)	25.8 (±0.1)	5.8 (±0.0)
**PI3K**δ	99.9 (±0.0)	89.7 (±0.8)	75.0 (±0.5)	56.8 (±0.9)	38.4 (±0.5)
**FXR**	100 (±0.0)	90.1 (±0.9)	79.0 (±0.8)	17.0 (±0.0)	2.9 (±0.0)
**PI3K**γ	99.8 (±0.0)	84.5 (±0.5)	79.2 (±0.1)	43.2 (±0.4)	21.2 (±0.2)
**JAK1**	98.5 (±0.1)	68.2 (±0.4)	67.9 (±0.4)	59.4 (±0.3)	19.0 (±0.1)
**JAK2**	99.2 (±0.0)	73.3 (±0.8)	70.5 (±0.5)	50.5 (±1.0)	18.3 (±0.2)
**JAK3**	99.5 (±0.1)	76.8 (±0.8)	73.3 (±0.2)	31.9 (±0.1)	10.0 (±0.1)
**PPAR**α	99.7 (±0.1)	73.0 (±0.5)	81.8 (±0.2)	22.0 (±0.2)	5.0 (±0.0)
**PPAR**δ	99.8 (±0.0)	73.5 (±0.7)	82.9 (±0.3)	26.5 (±0.2)	7.8 (±0.0)
**PPAR**γ	99.8 (±0.1)	77.4 (±0.1)	82.2 (±0.2)	31.9 (±0.1)	13.3 (±0.0)
**VDR**	99.9 (±0.1)	88.7 (±0.3)	47.5 (±0.5)	31.9 (±0.1)	8.7 (±0.0)

SI7 Training Data Set Extrapolation

To assess the extrapolation ability of DRAGONFLY-generated molecules beyond the confines of the training data set (which is a subset of ChEMBL [7]), we conducted a comparison of the Tanimoto similarity between the generated molecules and the most similar molecules found within both the training data set and an external data set (PubChem [28], excluding training set). Among the 10 out of 20 targets analyzed, the molecules generated by DRAGONFLY exhibited a higher average similarity to PubChem compounds compared to the training data set (Table S7).

Table S7: Average Tanimoto similarity in % (100% indicates identical fingerprints) for molecules generated with DRAGONFLY in comparison to the most similar molecules in the training data set (ChEMBL subset) and in an external data set (PubChem). The numbers represent mean and standard deviation for sets of 200 generated molecules.

Template	Training data (ChEMBL subset)	PubChem
**ABL**	55.0 (± 10.6)	54.9 (± 8.3)
**ER**β	49.5 (± 12.6)	52.7 (± 9.8)
**ER**α	53.1 (± 13.6)	54.5 (± 9.3)
**BRAF**	56.0 (± 11.7)	53.1 (± 8.8)
**FXR**	47.6 (± 8.9)	51.4 (± 8.4)
**LXR**α	45.2 (± 6.5)	48.2 (± 6.0)
**RAR**β	47.8 (± 9.3)	53.8 (± 10.3)
**RAR**α	46.9 (± 8.7)	51.3 (± 8.8)
**BTK**	54.2 (± 12.4)	52.5 (± 9.9)
**RXR**α	48.1 (± 10.2)	49.5 (± 7.5)
**PI3K**δ	50.8 (± 12.3)	48.9 (± 8.7)
**LXR**α	45.2 (± 6.5)	48.2 (± 6.0)
**LXR**β	45.4 (± 6.4)	48.7 (± 6.5)
**PPAR**α	55.3 (± 12.5)	54.8 (± 9.4)
**PPAR**δ	55.4 (± 12.0)	53.2 (± 7.2)
**PI3K**γ	55.6 (± 11.9)	53.8 (± 8.8)
**VDR**	47.5 (± 14.8)	48.5 (± 11.0)
**JAK1**	59.2 (± 16.1)	54.4 (± 10.6)
**JAK2**	57.5 (± 14.9)	54.0 (± 10.5)
**JAK3**	55.1 (± 13.8)	52.8 (± 8.9)

SI8 Amino Acid Sequence Identity

The sequence identity of the proteins in the training data set was analyzed with respect to the template of prospective study, *i.e.*, PPARγ. All PPAR proteins, including different sub-family members (*i.e.*, α, γ, and δ), species (*e.g.*, human, rat, mouse, bovine etc.) and isoforms have been removed from the training data. From the remaining proteins Thyroid hormone receptor β-1 and Liver X receptor (LXR)-β have been shown to yield the highest sequence identity, *i.e.*, 32.6% and 29.8%, respectively (Table S8). To calculate the identity values FASTA alignment was applied as implemented by the MBL-EBI search and sequence analysis tool [29].

Table S8: Sequence identity of the two most similar proteins in the training data set and PPARγ.

Sequence identity (%)	Target ID	Name	Species	ChEMBL ID	PDB IDs
32.6	271	Thyroid hormone receptor β-1	Homo sapiens	CHEMBL1947	1nq0, 1n46, 1nax, 1xzx, 1q4x, 1y0x, 1r6g, 2j4a, 3jzc, 3gws, 3imy
29.8	20113	Liver X receptor (LXR)-β	Homo sapiens	CHEMBL4093	1pqc, 1pq6, 3kfc, 3l0e, 4dk8, 4rak, 5jy3, 5kyj, 5i4v, 5kya, 6s4t, 6s4n, 6s4u, 6s5k

SI9 Biological Characterization

Figure S13 illustrates additional dose response curves for PPAR$\alpha/\gamma/\delta$.

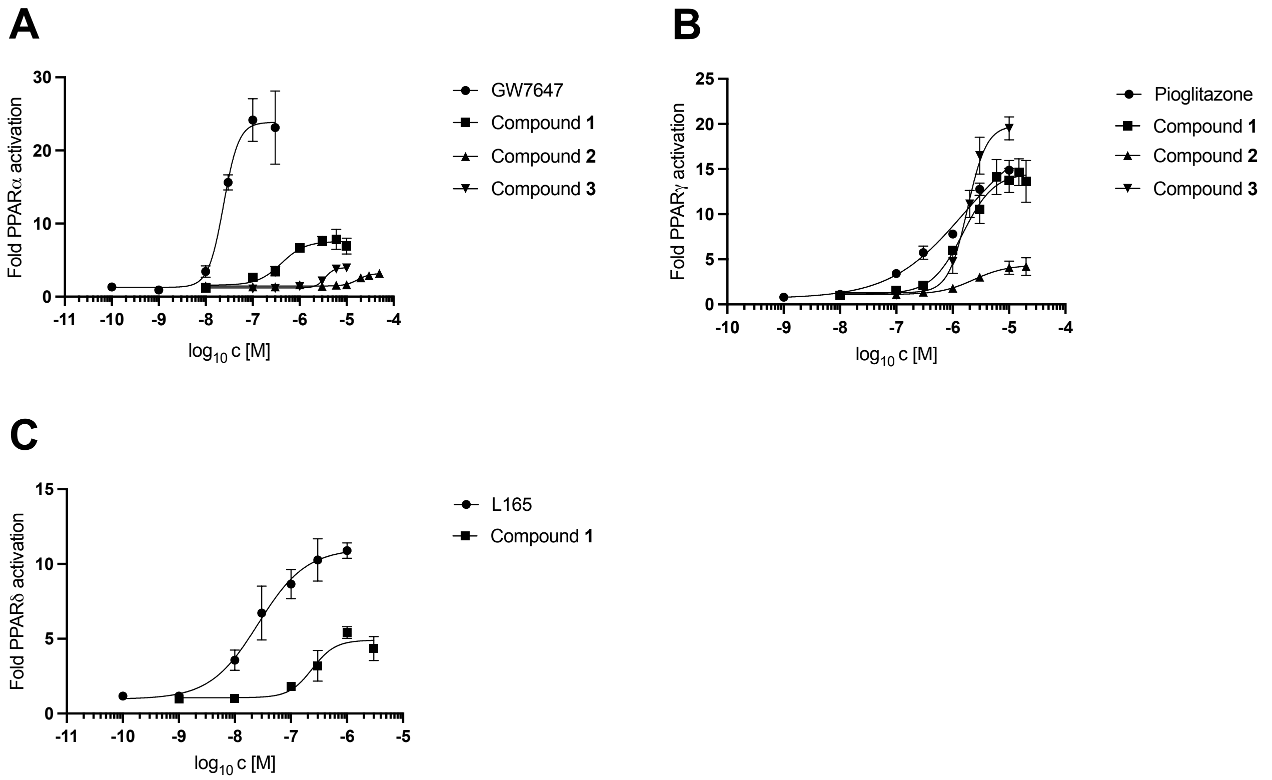

Figure S13: Dose response curves observed form hybrid gene reporter assays. EC_{50} values are illustrated for compounds **1** - **3** for PPAR$\alpha/\gamma/\delta$. For each plot the dose response curve of a positive control is added, *i.e.*, GW7647, Pioglitazone and L165. **A**: Dose response curves for PPARα. **B** Dose response curves for PPARγ. **C** Dose response curves for PPARδ. Source data are provided as a Source Data file.

Table S9-S12 list all targets, their species, inhibition (%) and assay type for the experiments conducted in the off-target screening.

Table S9: Inhibition or binding at a concentration of 10 µM for compound **1** in combination with safety-relevant off-targets. The targets are sorted in descending order by percentage inhibition / binding. The numbers for mean inhibition (%) represent single point measurements.

Species	Mean inhibition (%)	Assay type	Target
Human	95.0	Binding	PPARγ
Human	39.6	Enzymatic	GSK-3A
Human	20.2	Enzymatic	GSK-3B
Human	13.6	Binding	ESTROGEN RECEPTOR α
Human	13.3	Enzymatic	MONOAMINE OXIDASE-A
Rat	12.5	Binding	GABA-A (BENZODIAZAPINE BINDING SITE)
Human	10.9	Binding	κ OPIOID RECEPTOR
Human	10.9	Binding	HISTAMINE H2 RECEPTOR
Human	9.4	Binding	ADENOSINE A1 RECEPTOR
Human	9.3	Binding	CANNABINOID RECEPTOR CB1
Human	9.0	Binding	ADENOSINE A3 RECEPTOR
Human	8.4	Binding	MUSCARINIC RECEPTOR M1
HIV-1	8.2	Enzymatic	HIV-1 PROTEASE
Human	6.9	Binding	SEROTONIN TRANSPORTER
Human	6.6	Binding	5HT2B
Human	4.9	Binding	HISTAMINE H3 RECEPTOR
Human	4.4	Enzymatic	ABL1
Human	4.2	Binding	MUSCARINIC RECEPTOR M2
Human	4.0	Binding	β2-ADRENOCEPTOR
Human	3.6	Enzymatic	CDK2
Human	3.1	Enzymatic	PHOSPHODIESTERASE 4D2
Rat	2.3	Binding	GLYCINE RECEPTOR, STRYCHNINE INSENSITIVE
Human	1.7	Binding	alpha1A-ADRENOCEPTOR
Human	1.4	Enzymatic	PHOSPHODIESTERASE 3B
Human	1.2	Binding	CHOLECYSTOKININ 1 RECEPTOR

Table S10: Inhibition or binding at a concentration of 10 µM for compound **1** in combination with safety-relevant off-targets. The targets are sorted in descending order by percentage inhibition or binding. The numbers for mean inhibition (%) represent single point measurements.

Species	Mean inhibition (%)	Assay type	Target
Human	0.7	Binding	β1-ADRENOCEPTOR
Human	0.0	Binding	PROSTAGLANDIN F RECEPTOR
Human	0.0	Enzymatic	ACETYLCHOLINESTERASE
Human	-0.3	Enzymatic	ZAP70
Human	-1.6	Binding	ANDROGEN RECEPTOR
Human	-1.9	Binding	DOPAMINE D2 RECEPTOR (short)
Bovine	-3.1	Enzymatic	XANTHINE OXIDASE
Human	-3.2	Binding	α2A-ADRENOCEPTOR
Human	-3.3	Binding	DOPAMINE D1 RECEPTOR
Human	-3.3	Binding	NOREPINEPHRINE TRANSPORTER
Human	-3.3	Binding	5HT2A
Human	-3.7	Binding	NICOTINIC RECEPTOR, MUSCLE
Human	-3.8	Binding	GLUCOCORTICOID RECEPTOR
Rat	-4.7	Binding	CA2+ CHANNEL (DILTIAZEM SITE)
Human	-4.7	Binding	5HT3
Human	-6.9	Binding	NICOTINIC RECEPTOR, NEURONAL (α-BGTX insens.)
Human	-6.9	Binding	μ OPIOID RECEPTOR
Human	-7.0	Binding	HISTAMINE H1 RECEPTOR
Human	-7.5	Binding	ANGIOTENSIN II RECEPTOR 1
Human	-11.6	Enzymatic	ANGIOTENSIN CONVERTING ENZYME
Rat	-12.0	Binding	PCP RECEPTOR
Human	-20.6	Binding	5HT1A
Human	-21.7	Enzymatic	CYCLO OXYGENASE 2

Table S11: Inhibition or binding at a concentration of 10 µM for compound **2** in combination with safety-relevant off-targets. The targets are sorted in descending order by percentage inhibition / binding. The numbers for mean inhibition (%) represent single point measurements.

Species	Mean inhibition (%)	Assay type	Target
Human	89.6	Binding	PPARγ
Rat	57.8	Binding	GABA-A (BENZODIAZAPINE BINDING SITE)
Human	45.9	Enzymatic	CDK2
Human	22.7	Binding	5HT1A
Human	17.1	Binding	ADENOSINE A3 RECEPTOR
Rat	11.8	Binding	PCP RECEPTOR
Human	7.6	Binding	CHOLECYSTOKININ 1 RECEPTOR
Human	7.1	Binding	5HT2B
Human	7.0	Binding	β2-ADRENOCEPTOR
Human	6.9	Binding	β1-ADRENOCEPTOR
Human	6.9	Binding	MUSCARINIC RECEPTOR M2
Human	6.4	Binding	SEROTONIN TRANSPORTER
Human	6.2	Binding	μ OPIOID RECEPTOR
Human	4.9	Binding	α2A-ADRENOCEPTOR
Human	4.6	Binding	PROSTAGLANDIN F RECEPTOR
Human	4.4	Enzymatic	MONOAMINE OXIDASE-A
Human	3.5	Enzymatic	ZAP70
Human	3.1	Binding	ANDROGEN RECEPTOR
Human	2.9	Binding	α1A-ADRENOCEPTOR
Human	2.1	Binding	HISTAMINE H3 RECEPTOR
Human	2.0	Enzymatic	ACETYLCHOLINESTERASE
Human	0.9	Binding	NICOTINIC RECEPTOR, MUSCLE
Human	0.7	Binding	NOREPINEPHRINE TRANSPORTER
Bovine	0.4	Enzymatic	XANTHINE OXIDASE
Human	0.3	Binding	5HT3

Table S12: Inhibition or binding at a concentration of 10 µM for compound **2** in combination with safety-relevant off-targets. The targets are sorted in descending order by percentage inhibition / binding. The numbers for mean inhibition (%) represent single point measurements.

Species	Mean inhibition (%)	Assay type	Target
HIV-1	0.2	Enzymatic	HIV-1 PROTEASE
Human	-0.2	Binding	MUSCARINIC RECEPTOR M1
Human	-0.7	Binding	κ OPIOID RECEPTOR
Human	-1.8	Binding	ESTROGEN RECEPTOR alpha
Human	-2.1	Binding	GLUCOCORTICOID RECEPTOR
Human	-2.9	Binding	NICOTINIC RECEPTOR, NEURONAL (α-BGTX insens.)
Human	-3.1	Binding	HISTAMINE H2 RECEPTOR
Human	-3.2	Binding	CANNABINOID RECEPTOR CB1
Human	-3.6	Binding	ADENOSINE A1 RECEPTOR
Human	-3.8	Enzymatic	PHOSPHODIESTERASE 4D2
Human	-5.7	Binding	DOPAMINE D1 RECEPTOR
Human	-6.2	Binding	ANGIOTENSIN II RECEPTOR 1
Human	-6.7	Binding	HISTAMINE H1 RECEPTOR
Human	-6.8	Binding	DOPAMINE D2 RECEPTOR (short)
Rat	-8.3	Binding	GLYCINE RECEPTOR, STRYCHNINE INSENSITIVE
Human	-8.8	Binding	5HT2A
Human	-8.8	Enzymatic	GSK-3A
Human	-10.4	Enzymatic	CYCLO OXYGENASE 2
Human	-11.0	Enzymatic	ABL1
Rat	-11.7	Binding	CA2+ CHANNEL (DILTIAZEM SITE)
Human	-20.3	Enzymatic	GSK-3B
Human	-26.7	Enzymatic	PHOSPHODIESTERASE 3B
Human	-44.3	Enzymatic	ANGIOTENSIN CONVERTING ENZYME

SI10 Protein-Ligand Co-Crystallization

SI10.1 Constructs Details

The following construct was used for expression and co-crystallization. PPARγ (L204-Y477) (UniProt ID: P37231-2): MGSS-6His-SG-TEV-(L204-Y477). Molecular weight: 33465 Da.

SI10.2 Large-Scale Expression of PPARγ in *E. coli* BL-21(DE3) cells

Large-scale expression of human PPARγ was done in *E. coli* BL-21 (DE3) cells. Competent cells were transformed with plasmid encoding the PPARγ construct, following standard procedures. A single colony from the transformation plate was used to start an overnight preculture. On the next day the cells were used to inoculate 4 x 1 L of LB supplemented with appropriate antibiotics. The protein expression was induced with 0.1 mM IPTG when the cell density reached $OD_{600} = 0.6$. The expression was performed overnight, at 18 °C. The cells were harvested *via* centrifugation at 6000 g for 15 min, at 4 °C.

SI10.3 Purification of PPARγrotein

The cells from 4 L of expression culture were resuspended in IMAC A buffer (50 mM HEPES pH 7.9, 100 mM NaCl, 5 mM TCEP), supplemented with 1 mM PMSF, 0.25 mg·mL^{-1} lysozyme and Pierce Universal Nuclease. The sample was sonicated on ice for 15 min in total, using 5 s on/5 s off pulses. Cleared supernatant containing soluble material was isolated *via* centrifugation at 30'000 g for 45 min at 4 °C. The final sample was loaded overnight at 4 °C on a 10 mL TALON IMAC column (Takara Bio), equilibrated in IMAC A buffer supplemented with 5 mM imidazole. The next day the column was connected to an ÄKTA system and non-specifically bound proteins were washed out in a three-step gradient of IMAC B buffer (IMAC A buffer + 200 mM imidazole), resulting in concentrations of 5 mM, 10 mM, and 20 mM imidazole. Elution of the protein of interest was performed in a linear gradient of IMAC buffer B from 20–150 mM imidazole, followed by a linear gradient up to 200 mM imidazole. Fractions collected during the IMAC purification were analyzed on SDS-PAGE.

SI10.4 Crystallization

Co-crystals of PPARγ were grown using 6 mg·mL^{-1} protein in buffer: 20 mM Tris-HCl pH 8.0, 1 mM TCEP, 0.5 mM EDTA and 1 mM design **1** mixed with equal amounts of reservoir: 0.1 M Tris-HCl pH 7.5 and 1.6 M ammonium sulphate, similar to that described by Willems *et al* 2021. [30] 150 + 150 nl sitting drops were equilibrated over 40 µL of reservoir in a MRC 3x96-well plate at 20 °C. Crystals appeared overnight. Figure S14 depicts the observed crystals.

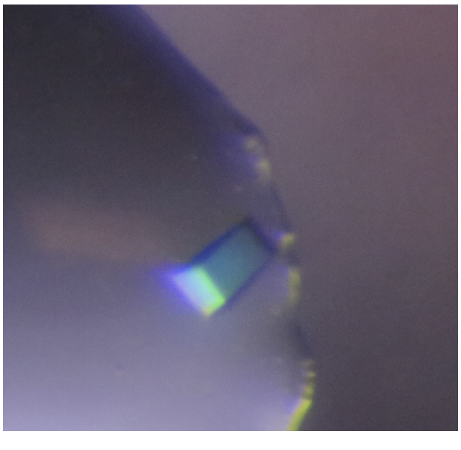

Figure S14: Crystal used for data collection growing at the border of the crystallization drop visualized under polarised light (about 80 x 150 µm).

SI10.5 Freezing

After four days, 1.5 µL of cryo-solution (0.1 M Tris-HCl pH 7.5, 1.6 M ammonium sulphate, 0.25 mM TCEP, 1 mM design **1** and 31.2% glycerol) was added to the drop. Thereafter the crystal was mounted in a nylon loop and flash-cooled in liquid nitrogen.

SI10.6 Data Collection

The best data sets of PPARγ in complex with design **1** were collected at station I03, Diamond Light Source, Didcot, UK at 100 K and $\lambda = 0.9763$ Å. 3600 images were collected with an oscillation range of 0.1° per image. The beamline is equipped with a Dectris EIGER XE 16M detector. Data were processed using the xia2 DIALS pipeline, [31] which includes the software XDS. [32] The data was finally merged, converted to structure factors and cut in resolution using the software Aimless [33]; see the Summary in Table S13.

Table S13: Key quality indicators for the PPARγ co-crystal structure with design **1**.

Resolution (Å)	44.24 - 1.85 (1.89 - 1.85)
Wavelength (Å)	0.9763
Space group	C2
Unit cell	a = 93.4, b = 60.6, c = 117.9 Å, β = 102.9°
Completeness (%)	100.0 (100.0)
Multiplicity	7.0 (7.2)
No. of observations / unique reflections	384 207 / 55 065 (24 153 / 3 367)
<I/σ(I)>	17.1 (1.0)
CC(1/2) (%)	99.9 (90.3)
Rmerge (I) (%)	5.9 (79.8)
Rpim (I) (%)	2.4 (32.0)b
Rmodel (F) (%)	17.1 (33.2)
Rfree (F) (%)	21.3 (33.1)
No. of non-hydrogen atoms	4 549
No. of water molecules, sulphates, glycerol	177 / 5 / 4
**rms deviations from ideal geometry**	
Bond lengths (Å)	0.014
Bond angles (°)	2.1
Mean B-factor protein chain A, chain B (Å2)	43.4 / 53.1
Mean B-factor water molecule/sulphates/ glycerol (Å2)	46.6 / 57.7 / 70.5
Mean B-factor ligand in chain A (Å2)	50.5
**Ramachandran plot quality**	
Favoured regions (%)	98.6
Allowed regions (%)	1.4
Outliers (%)	-

SI10.7 Structure Determination and Refinement

The structure was determined using molecular replacement and the software Phaser. [34] The search model was the coordinates of the complex between PPARγ and rosiglitazone determined to 1.74 Å with the compound removed. Two molecules were found in the asymmetric unit. The structure was refined using Refmac5 [35] and model building was carried out in Coot. [36] After rebuilding, difference density maps showed that one compound was clearly bound to chain A whereas only minor density was found in the binding site in chain B. Ligand coordinates and a restraints file for use in Refmac5 were generated using AceDRG. [37]

SI10.8 PPARγ Co-Crystal Structure Complex

The structure of PPARγ in complex with design **1** includes amino acid residues 203 - 477 in chain A and 207 - 477 in chain B, with the exception of residues 265 - 274 in chain A and residues 243 and 269 - 275 in chain B since these are not visible in the electron density. PPARγ in chain A binds design **1** whereas PPARγ in chain B is empty (Figure S15a). In chain B the site is partially blocked by the carboxyl terminus of Y477, which binds to the site at approximately the same position as the carboxyl group of design **1** (Figure S15b). With exception of the last 20 amino acids the overall structures of PPARγ in chain A and chain B are very similar. In addition to the compound the structure includes 177 water molecules, five sulphate ions and four glycerol molecules. Some density have been left unmodeled in the site of chain B and also in a loop in chain A (close to D243). The unmodeled density close to D243 may be an alternative conformation of the loop.

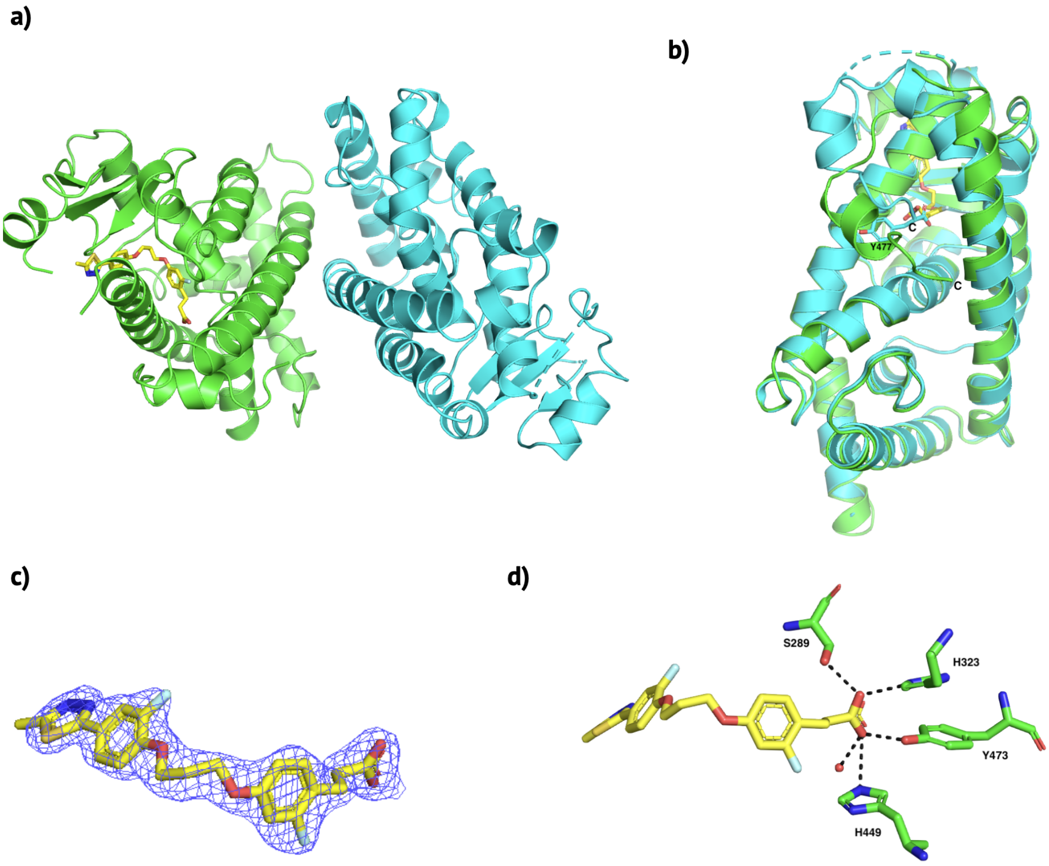

Figure S15: **A** Refined structure of the complex showing compound **1** bound to chain A and chain B empty (chain A in green and chain B in cyan). **B** Overlay of chain B onto chain A. The C-terminal residue Y477 in chain B (cyan sticks) has a position very close to the carboxyl group of compound **1** (yellow sticks) The C-terminus marked (C). **C** Close-up view of compound 1. The 2m|Fo|-D|Fc| electron density map contoured at 1σ is shown in blue around the compound. **D** Polar interactions (as calculated by PyMOL[38]) are shown in black broken lines. There are four hydrogen bonds to the side chains of S289, H323, H449 and Y473 and one hydrogen bond to a water molecule.

SI11 Absorption Distribution Metabolism and Excretion and Toxicity Data

To assess the drug-like characteristics of the newly designed PPAR modulators, a series of tests were conducted covering various aspects of absorption, distribution, metabolism, and excretion (ADME) properties. Specifically, these assessments included the parallel artificial membrane permeability assay (PAMPA) with permeation coefficients ($PAMPA_{PEFF}$) [39], Log$D$ measurements, microsomal clearance [40, 41], hepatocyte clearance [42], P-glycoprotein (Pgp) efflux ratio and binding [43] and the unbound free fraction [43]. Table S14 provides an overview of the observed ADME values for compounds **1** and **2**. Furthermore, the evaluation of inhibition potential against cytochrome P450 isoenzymes (CYP) proteins was investigated. For seven selected CYP proteins (*i.e.*, Cyp3A4, Cyp1A2, Cyp2B6, Cyp2C9, Cyp2D6, Cyp2C19, and Cyp2C8) the half maximal inhibitory concentration (IC50) was determined. [44] (Table S15). The two designs (**1** and **2**) did not echibit any relevant activity in the CYP assays was found.

Table S14: Absorption distribution metabolism and excretion (ADME) properties of compounds **1** and **2**. The values for $PAMPA_{PEFF}$, Log$D$, protein binding, P-glycoprotein (Pgp) efflux ratio and permeability, and hepatocyte clearance are presented as the mean and standard deviation, with a sample size of $N = 3$, *i.e.*, 3 technical replicates each. The values for microsomal clearance and CYP-inhibition represent single point measurements.

	**Aleglitazar**	**1**	**2**
Molecular weight (g·mol^{-1})	437.5	434.5	432.5
Lipophilicity (Log$D$ pH 7.4)	1.4 (±0.3)	1.5 (±0.3)	1.7 (±0.6)
$PAMPA_{PEFF}$ (cm·s^{-1}·10^{-6})	6.0 (±1.2)	14 (±3)	3.9 (±0.4)
Microsomal clearance, human (µL·min^{-1}·mg^{-1} protein)	≥10	≥10	≥10
Microsomal clearance, mouse (µL·min^{-1}·mg^{-1} protein)	≥10	≥10	≥10
Microsomal clearance, rat (µL·min^{-1}·mg^{-1} protein)	≥10	≥10	≥10
Protein binding human (% free fraction) (%)	-	0.21	0.42
PgP Transport mouse apical efflux ratio	-	1.6	1.2
PgP Transport mouse permeability (nm·sec^{-1})	-	15	60
Hepatocyte clearance human (µL·sec^{-1}·10^{6} Cells^{-1})	-	19	19

Table S15: Half maximal inhibitory concentration (IC50) in µM for compounds **1** and **2** for seven selected CYP proteins, *i.e.*, Cyp3A4, Cyp1A2, Cyp2B6, Cyp2C9, Cyp2D6, Cyp2C19, and Cyp2C8.

**Molecule**	**Enzyme**	**Substrate**	**IC50 (µM)**
**1**	CYP3A4	MIDAZOLAM	>20
	CYP1A2	PHENACETIN	>20
	CYP2B6	BUPROPION	>20
	CYP2D6	DEXTROMETHORPHAN	>20
	CYP2C9	DICLOFENAC	>20
	CYP2C19	S-MEPHENYTOIN	>20
	CYP2C8	AMODIAQUINE	>20
**2**	CYP3A4	MIDAZOLAM	>20
	CYP1A2	PHENACETIN	>20
	CYP2B6	BUPROPION	>20
	CYP2D6	DEXTROMETHORPHAN	>20
	CYP2C9	DICLOFENAC	>20
	CYP2C19	S-MEPHENYTOIN	>20
	CYP2C8	AMODIAQUINE	1.75

Compounds **1** and **2** were validated for cytotoxicity on HEK293T cells. Figure S16 depicts the measured cytotoxicity at two time points (*i.e.*, 16h and 24h), 10 different concentrations (*i.e.*, 0.05 - 20 µM), and 5 different numbers of cells per well (*i.e.*, 1000, 3000, 5000, 10000, and 20000).

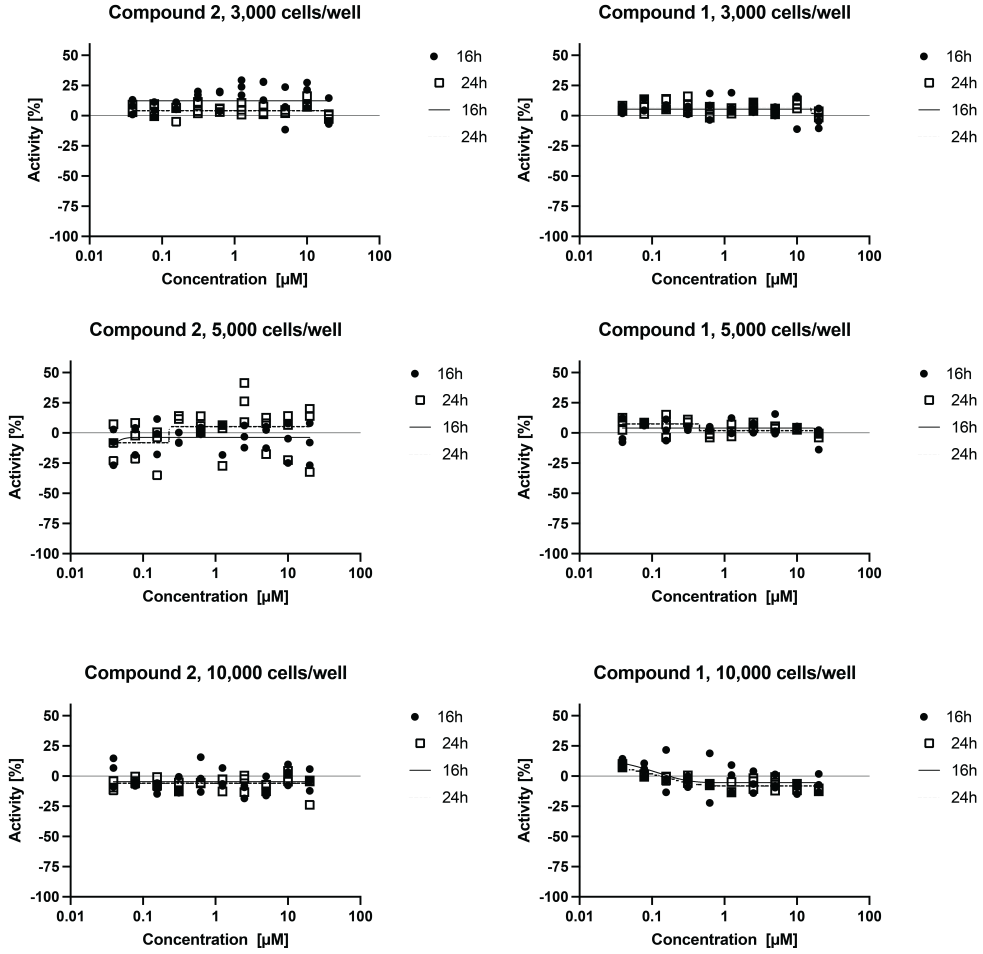

Figure S16: Cytotoxicity assay ($N = 3$) on HEK293T cells for compounds **1** and **2** for two time points (*i.e.*, 16h and 24h), 10 different concentrations (*i.e.*, 0.05 - 20 μM), and 3000 - 10000 cells per well. Scale reference: The axes are scaled through neutral control (*i.e.*, Dimethylsulfoxide [DMSO], set to 0) and inhibitor control wells (*i.e.*, 20μM Staurosporine [45], set to -100). Source data are provided as a Source Data file.

SI12 Absolute Free Binding Energy Calculations

Figure S17 illustrates the reference ligands employed for the absolute protein–ligand binding free-energy perturbation (ABFEP) calculations as well as their respective Gibbs free energy values (ΔG).

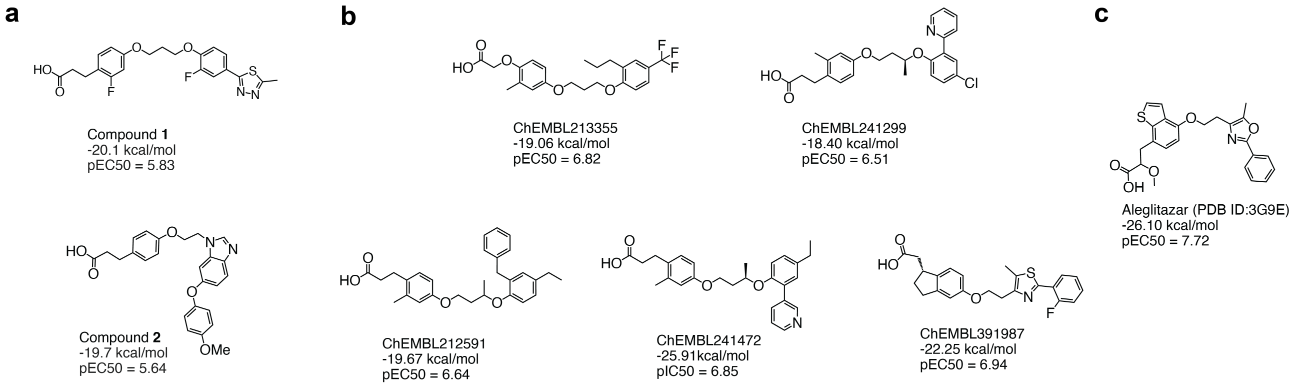

Figure S17: Illustration depicting the molecules subjected to absolute protein–ligand binding free-energy perturbation (ABFEP) calculations. **a**: Molecular structures of Compound **1** and **2**. **b**: Molecular structures of ChEMBL391987, ChEMBL241472, ChEMBL241299, ChEMBL213355, and ChEMBL212591. **c**: Aleglitazar [27].

SI13 Chemical Synthesis

SI13.1 Experimental Procedures and Analytical Data

Designs **1** and **2**, and derivative **3** were synthesized *via* multi-step batch synthesis in mg-g scale.

SI13.2 Reagent and purification information

Unless otherwise noted, all reactions were set up and conducted under an inert atmosphere of Argon. The atmosphere was introduced by applying several cycles of Argon/vacuum/Argon. Before conducting a work-up, heated reactions were cooled to room temperature without using any external device or ice baths. Reaction reflux conditions were obtained using DrySyn® heating blocks (Radnor, PA, US) equipped with a standard thermometer. Solvent evaporation was performed under reduced pressure on a Büchi (Flawil, CH) rotary evaporator. Microwave (µw) reactions were carried out in a Biotage (Uppsala, Sweden) initiator and reactor. All chemicals were purchased from Sigma Aldrich, Fluorochem Ltd, Apollo Chemicals Ltd, Fluka Cat. GmbH, and used as received. Purification by flash column chromatography was performed using Gel de silice 60 (0.063 - 0.200 mm) from Merck (Darmstadt, DE). All reactions were monitored by TLC using pre-coated silica gel aluminium plates from Macherey-Nagel (Oensingen, CH) and visualized by ultraviolet (UV) light at 254 and 366 nm. Eluent solvents, gradients and cartridge sizes for flash chromatography are described for each experiment.

SI13.3 Analytical information

All compounds were characterized by nuclear magnetic resonance (NMR) spectroscopy and high-resolution mass spectrometry (HRMS).
NMR spectra were recorded on a Bruker AV 400 or Bruker AV 500 spectrometer (Bruker Corporation, Billerica, MA, USA). NMR data are reported as follows: chemical shift in reference to the residual solvent peak (δ ppm), multiplicity (s = singlet, d = doublet, br d = broad doublet, dd = doublet of doublet, br dd = broad doublet of doublet, t = triplet, br t = broad triplet, m = multiplet), coupling constant (Hz), and integration. ^{1}H NMR and ^{13}C NMR residual solvent peaks in respective deuterated solvents. ^{1}H NMR residual solvent peaks in respective deuterated solvents for $CDCl_3$ at 7.26 ppm, MeOH at 3.31 ppm and THF-d_8 at 1.73 ppm and 3.58 ppm. ^{13}C NMR residual solvent peaks in respective deuterated solvents for $CDCl_3$ at 77.00 ppm, MeOH at 49.00 ppm, and THF-d_8 at 25.37 and 62.57 ppm.
LC-MS high-resolution (HRMS) spectra were recorded on a Bruker (Billerica, US) maXis – ESI-Qq-TOF-MS. The purity of all compounds was determined by reversed-phase high-performance liquid chromatography (HPLC)-MS with UV and ESI-MS detection on a Shimadzu (Kyoto, JP) liquid chromatography-mass spectrometer (LC-MS) 2020 system with a Nucleodur C18 HTec column (150 × 3 mm, 5 µm, 110 Å) from Macherey-Nagel (Düren, DE); and a linear 50–95% MeCN in water (MilliQ) gradient containing 0.1% formic acid over 16 minutes with a flow rate of 0.5 ml min^{-1} at 30 °C. No impurities were detected in MS or UV for all compounds submitted to biological testing.

SI13.4 Experimental procedures and analytical data

Synthesis of Compound 1

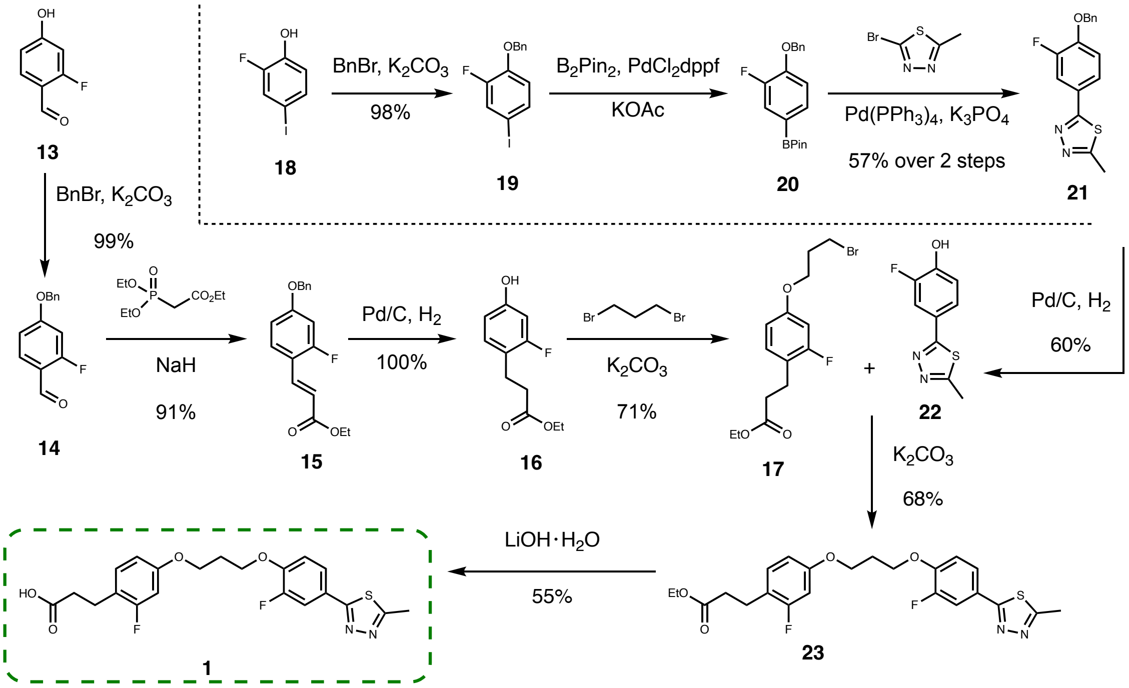

Figure S18: **Chemical synthesis of compound 1.** The synthesis of compound **1** employed a convergent approach, starting from commercially available building blocks **13** and **18**, and spanning a total of 10 steps. The overall yield achieved for the synthesis of **1** was 12%.

Synthesis of compound 14

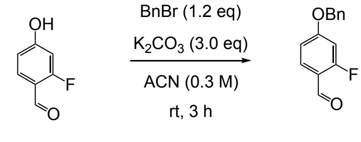

13 14

Figure S19: Synthesis scheme of **14**.

2-Fluoro-4-hydroxybenzaldehyde (**13**, 10.7 mmol, 1.0 eq.) was dissolved in MeCN (0.3 M) under vigorous stirring. Once a homogeneous solution formed, K_2CO_3 (32.1 mmol, 3.0 eq.) was added in one portion. After ten minutes, Benzyl bromide (16.1 mmol, 1.5 eq.) was added drop-wise to the solution and the resulting reaction mixture was stirred for three hours at room temperature. The heterogeneous mixture was separated by filtration and, after washing with DCM, the solid was discarded. Evaporation of solvents delivered the crude material, which was purified by flash chromatography. **System of elution**, from 95% Pentane-5% EtOAc to 90% Pentane-10% EtOAc. **Yield** 99%. **Rf**: 0.38 at 95% Pentane-5% EtOAc. **Physical appearance:** White solid. **Melting point**: 96-97 °C.

^{1}H NMR (400 MHz, $CDCl_3$) δ (ppm) 10.21 (s, 1H), 7.82 (t, J = 8.36 Hz, 1H), 7.42-7.34 (m, 5H), 6.86 (dd, J = 2.56, 8.88 Hz, 1H), 6.72 (dd, J = 2.28, 12.44 Hz, 1H), 5.13 (s, 2H).

^{13}C NMR (101 MHz, $CDCl_3$) δ (ppm) 185.85 (d, J = 9.6 Hz), 166.14 (d, J = 260.08 Hz), 165.17 (d, J = 11.97 Hz), 135.31, 130.13, 128.77, 128.49, 127.47, 118.00 (d, J: 8.77 Hz), 111.87, 102.31 (d, J = 23.94 Hz), 70.68.

^{19}F NMR (376 MHz, $CDCl_3$) δ (ppm)-119.02.

HRMS (ESI) $[M+Na]^+$ m/z: 253.0635 calculated for $C_{14}H_{11}FNaO_2$, found: 253.0634.

Synthesis of compound 16

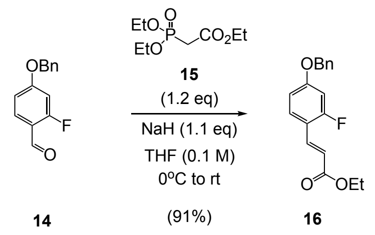

Figure S20: Synthesis scheme of **16**.

In a flamed dried three-neck round bottom flask, NaH (9.6 mmol, 1.1 eq.) was dissolved in THF (0.1 M). Once a homogeneous solution formed, the reaction mixture was cooled to 0 °C. After ten minutes, Triethyl phosphonate (**15**, 10.43 mmol, 1.2 eq.) was added drop-wise and the reaction mixture was stirred for 30 minutes at 0 °C. Then, **14** (8.7 mmol, 1.0 eq.) in THF (0.5 M) was added drop-wise at 0 °C. After 15 minutes, the reaction mixture was removed from the cooling bath and stirred overnight at room temperature. After evaporation of solvents, the crude was re-dissolved in EtOAc and a precipitate formed. The solid was separated by filtration and discarded. The liquid organic layer was washed with a saturated aqueous solution of NH_4Cl and water. The organic layers were combined and dried over $MgSO_4$. Evaporation of solvents delivered the crude material, which was purified by flash chromatography. **System of elution**: from 5% EtOAc-95% Pentane to 10% EtOAc-90% Pentane. **Yield**: 91%. **Rf**: 0.54 at 10% EtOAc-90% Pentane. **Physical appearance**: Brownish solid. **Melting point**: 58-59 °C.

^{1}H NMR (400 MHz, $CDCl_3$) δ (ppm) 7.75 (t, J = 16.2 Hz, 1H), 7.47-7.33 (m, 6H), 6.86 (dd, J = 2.54, 8.62 Hz, 1H), 6.71 (dd, J = 2.28, 12.44 Hz, 1H), 6.42 (t, J = 15.96 Hz, 1H), 5.07 (s, 2H), 4.26 (q, J = 17.09 Hz, 2H), 1.34 (t, J = 7.08 Hz, 3H).

^{13}C NMR (101 MHz, $CDCl_3$) δ (ppm) 167.14, 162.26 (d, J = 255.29 Hz), 161.47(d, J = 13.56 Hz), 137.04, 135.86, 129.89 (d, J = 8.78 Hz), 128.68, 128.29, 127.43, 118.20 (d, J = 9.56 Hz), 115.37 (d, J = 14.35 Hz), 111.47, 102.77 (d, J = 26.33 Hz), 70.40, 60.40, 14.29.

^{19}F NMR (376 MHz, $CDCl_3$) δ (ppm)-111.58.

HRMS (ESI) $[M+H]^+$ m/z: 301.1234 calculated for $C_{18}H_{18}FO_3$, found: 301.1236.

Synthesis of compound 17

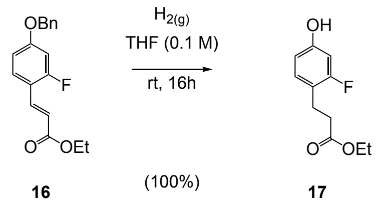

Figure S21: Synthesis scheme of **17**.

In a dried flamed three-necked round bottom flask, **16** (6.7 mmol, 1.0 eq.) was dissolved in dry THF (0.1 M) at room temperature. Then, Pd/C (10%) (0.7 mmol, 0.1 eq.) was added, before an atmosphere of Hydrogen was introduced. The resulting mixture was stirred overnight at room temperature. After the Hydrogen atmosphere was broken, the Palladium catalyst was removed by filtration with Celite and rinsing with EtOAc. Then the organic phase was washed with an aqueous saturated solution of NH_4Cl and water. The organic layers were combined and dried over $MgSO_4$. Evaporation of solvents delivered the crude material, which was used for the next step without further purification. **Yield**: quantitative. **Rf**: 0.59 at 10% EtOAc-90% Pentane. **Physical appearance**: Colorless oil.

^{1}H NMR (400 MHz, CDCl$_3$) δ (ppm) 7.05-7.00 (m, 1H), 6.55-6.50 (m, 2H), 5.78 (s, 1H), 4.13 (q, J = 7.09 Hz, 1H), 2.89 (t, J = 7.6 Hz, 2H), 2.59 (t, J = 7.6 Hz, 2H), 1.23 (t, J = 7.22 Hz, 3H).

^{13}C NMR (101 MHz, CDCl$_3$) δ (ppm) 173.99, 161.41 (d, J: = 246.52 Hz), 155.89 (d, J = 11.97 Hz), 130.85 (d, J = 8.77 Hz), 118.49 (d, J = 16.76 Hz), 111.08, 103.11 (d, J = 25.53 Hz), 60.89, 34.84, 23.99, 14.02.

^{19}F NMR (376 MHz, CDCl$_3$) δ (ppm) -116.55.

HRMS (ESI) $[M+Na]^+$ m/z: 235.0741 calculated for $C_{11}H_{13}FNaO_3$, found: 235.0742.

Synthesis of compound 19

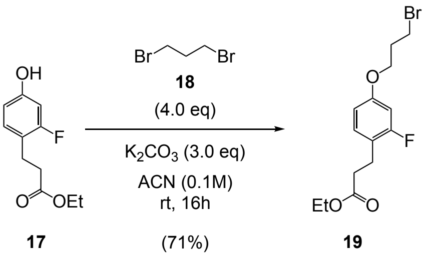

Figure S22: Synthesis scheme of **19**.

Compound **17** (6.5 mmol, 1.0 eq.) was dissolved in MeCN (0.1 M) under vigorous stirring at room temperature. Then, 1,3-di-bromopentane (**18**, 25.8 mmol, 4.0 eq.) was added drop-wise under vigorous stirring to the mixture. After ten minutes, K_2CO_3 (19.4 mmol, 3.0 eq.) was added under vigorous stirring at room temperature. After an additional ten minutes, the reaction mixture was heated and refluxed overnight. The solid from the heterogeneous system was removed by filtration, and rinsing with DCM. Solvent was removed under reduced pressure to obtain the dried crude of the reaction which was purified by flash chromatography. **System of elution**: 5% EtOAc-95% Pentane. **Yield**: 71%. **Rf**: 0.59 at 10% EtOAc-90% Pentane. **Physical appearance**: Colorless Oil.

^{1}H NMR (400 MHz, CDCl$_3$) δ (ppm) 7.12-7.07 (m, 1H), 6.63-6.58 (m, 2H), 4.12 (q, J = 7.09 Hz, 2H), 4.06 (t, J = 5.7 Hz, 2H), 3.59 (t, J = 6.46 Hz, 2H), 2.90 (t, J = 7.6 Hz, 2H), 2.58 (t, J = 7.8 Hz, 2H), 2.33-2.27 (m, 2H), 1.23 (t, J = 7.08 Hz, 3H).

^{13}C NMR (101 MHz, CDCl$_3$) δ (ppm) 172.72, 161.44 (d, J = 246.52 Hz), 158.45 (d, J = 11.97 Hz), 130.84 (d, J = 8.78 Hz), 119.45 (d, J = 16.76 Hz), 110.06, 102.21 (d, J = 25.53 Hz), 65.53, 60.36, 34.65, 32.15, 29.79, 23.96, 14.16.

^{19}F NMR (376 MHz, CDCl$_3$) δ (ppm) -116.21.

HRMS (ESI) $[M+H]^+$ m/z: 333.0496 calculated for $C_{14}H_{19}BrFO_3$, found: 333.0495.

Synthesis of Compound 21

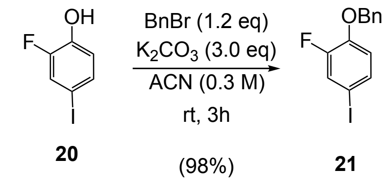

Figure S23: Synthesis scheme of **21**.

Compound **20** (6.3 mmol, 1.0 eq.) was dissolved in MeCN (0.3 M) under vigorous stirring at room temperature. Once a homogeneous solution was formed, K_2CO_3 (18.9 mmol, 3.0 eq.) was added under vigorous stirring in one portion. After ten minutes, Benzyl bromide (1.2 eq, 7.56 mmol) was added in one portion, and the resulting mixture was stirred for three hours at room temperature. After that, the solid from the heterogeneous system was discarded by filtration rinsing with EtOAc. The solvent was removed by reduced pressure and the dried crude material and redissolved in EtOAc. The organic phase was washed with an aqueous solution of HCl (1.0 M) and water. The organic layers were combined and dried over $MgSO_4$. Evaporation of solvents delivered the crude material, which was purified by flash chromatography. **System of elution**: from 1% EtOAc 99% Pentane to 5% EtOAc 95% Pentane. **Yield**: 98%. **Rf**: 0.54 at 5% EtOAc 95% Pentane. **Physical appearance**: White solid. **Melting point**: 72-73 °C.

^{1}H NMR (400 MHz, $CDCl_3$) δ (ppm) 7.42-7.34 (m, 5H), 7.35-7.31 (m, 2H), 6.74 (t, J = 8.6 Hz, 1H), 5.12 (s, 2H).

^{13}C NMR (101 MHz, $CDCl_3$) δ (ppm) 152.76,(d, J = 252.90 Hz), 146.84 (d, J = 11.17 Hz), 136.00, 133.27 (d, J = 3.19 Hz), 128.65, 128.24, 127.37, 125.35 (d, J = 21.53 Hz), 117.46, 81.69 (d, J = 8.78 Hz), 71.36.

^{19}F NMR (376 MHz, $CDCl_3$) δ (ppm) -130.69.

HRMS (ESI) $[M+Na]^+$ m/z: 350.9653 calculated for $C_{13}H_{10}FINaO$, found: 350.9656.

Synthesis of compound 24

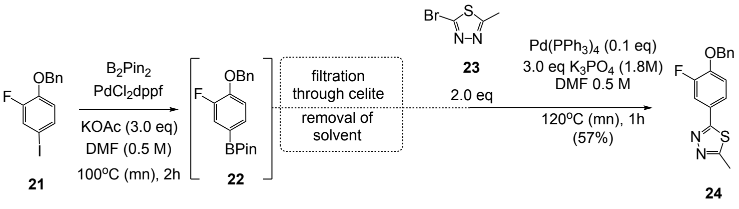

Figure S24: Synthesis scheme of **24**.

In a flame-dried microwave reactor, **21** (3.1 mmol, 1.0 eq.), Bispinacolatodiboron (5.5 mmol, 1.8 eq.), KOAc (9.2 mmol, 3.0 eq.) and $PdCl_2dppf$ (0.2 mmol, 0.05 eq.) were added. Next, degassed DMF (6 mL) was added through a syringe, under vigorous stirring to achieve a final reaction concentration of 0.5 M. The resulting mixture of the reaction was stirred at room temperature. Then, the vessel was placed in a microwave reactor and heated to 100 °C (30 watts) for 90 minutes. Then, the solid from the resulting heterogeneous system was discarded by filtration with Celite and rinsing with EtOAc. The solvent was evaporated and the crude material **22** redissolved under vigorous stirring and room temperature in DMF (0.5 M). Next, 2-bromo-5-methyl-1,3,4-thiadiazole (6.1 mmol, 2.0 eq.) and $Pd(PPh_3)_4$ (0.3 mmol, 0.1 eq.) were added, followed by the addition of an aqueous solution of K_3PO_4 (9.2 mmol, 1.8 M, 3.0 eq.) under vigorous stirring at room temperature. The reaction vessel was subjected to the microwave reactor, where it was heated for 60 minutes at 120 °C (30 watts). The solid from the heterogeneous system was discarded by filtration through Celite and rinsed with EtOAc. The solvent was evaporated and the crude material redissolved in EtOAc. The organic phase was washed with 1.0 M HCl solution and water. The organic phases were combined and dried over $MgSO_4$. Evaporation of the solvents gave the crude material, which was purified by flash chromatography. **System of elution**: from 10% EtOAc-90% Pentane to 30% EtOAc-70% Pentane. **Yield**: 57%. **Rf**: 0.18 at 30% EtOAc-70% Pentane. **Physical appearance**: Yellow pale solid. **Melting point**: 120-121 °C.

^{1}H NMR (400 MHz, $CDCl_3$) δ (ppm) 7.72 (dd, J = 2.28, 11.68 Hz, 1H), 7.59-7.56 (m, 1H), 7.46-7.43 (m, 2H), 7.41-7.32 (m, 3H), 7.05 (t, J = 8.6 Hz, 1H), 5.20 (s, 2H), 2.79 (s, 3H).

^{13}C NMR (101 MHz, $CDCl_3$) δ (ppm) 167.40, 164.47, 152.26 (d, J = 248.50 Hz), 148.89 (d, J = 11.49 Hz), 135.80, 126.68, 128.31, 127.36, 124.18, 123.63 (d, J: 7.42 Hz), 115.53 (d, J = 22.80 Hz), 115.42, 71.21, 15.20.

^{19}F NMR (376 MHz, $CDCl_3$) δ (ppm) -132.23.

HRMS (ESI) $[M+H]^+$ m/z: 301.0805 calculated for $C_{16}H_{14}FN_2OS$, found: 301.0803.

Synthesis of Compound 22

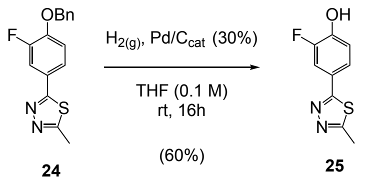

Figure S25: Synthesis scheme of **22**.

In a flamed dried three-necked round bottom flask, **24** (1.7 mmol, 1.0 eq.) was added at room temperature, followed by dry THF (17 mL) under vigorous stirring to give a reaction concentration of 0.1 M. Once a homogeneous solution was formed, the Palladium catalyst Pd/C (0.5 mmol, 0.3 eq.) was added in one portion. Next, a Hydrogen atmosphere through cycles of Hydrogen/vacuum/Hydrogen was introduced. The reaction mixture was stirred overnight at room temperature. Then, the Hydrogen atmosphere was broken and the Palladium catalyst was removed through filtration with Celite and rinsing with acetone. The solvent was evaporated and Et_2O was added to promote the formation of a precipitate, which corresponded to the desired product (**25**). The precipitate was washed with Et_2O several times. Subsequent evaporation of solvents delivered the desired product (**22**) in 60% yield, which was used for the next step without further purification.

^{1}H NMR (400 MHz, $CDCl_3$) δ (ppm) 7.64 (dd, J = 2.28, 11.64 Hz, 1H), 7.00 (t, J = 8.62 Hz, 1H), 2.76 (s, 3H).

^{19}F NMR (376 MHz, CD_3OD) δ (ppm) -137.74.

HRMS (ESI) $[M+H]^+$ m/z: 211.0336 calculated for $C_9H_8FN_2OS$, found: 211.0338.

Synthesis of compound 26

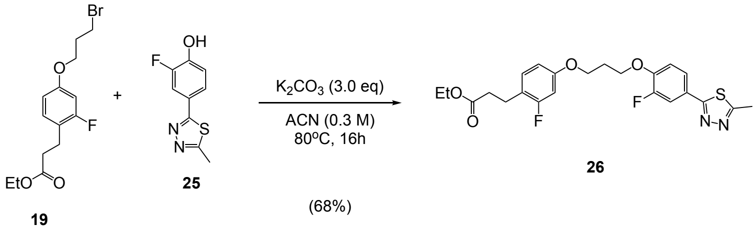

Figure S26: Synthesis scheme of **26**.

Compound **25**, (0.6 mmol, 1.0 eq.) was dissolved in MeCN (0.3 M). Next, Bromoalkane derivate **19** (0.7 mmol, 1.2 eq.) was added under vigorous stirring. Once a homogeneous solution formed, K_2CO_3 (1.8 mmol, 3.0 eq.) was added under vigorous stirring at room temperature. After ten minutes, the reaction mixture was refluxed during overnight. The solid from the heterogeneous mixture was discarded by filtration rinsing with EtOAc. Solvent was removed by reduced pressure, which was purified by flash chromatography. **System of elution**: from 30% EtOAc-70% Pentane up to 50% EtOAc-50% Pentane. **Yield**: 68%. **Rf**: 0.32 at 50% EtOAc-50% Pentane. **Physical appearance**: White solid. **Melting point**: 97-98 °C.

^{1}H NMR (400 MHz, CDCl$_3$) δ (ppm) 7.68 (dd, J = 2.28, 11.64 Hz, 1H), 7.59 (dd, J = 1.66, 8.42 Hz, 1H), 7.10-7.06 (m, 1H), 7.02 (t, J = 8.48 Hz, 1H), 6.63-6.58 (m, 2H), 4.26 (t, J = 6.1 Hz, 2H), 4.15-4.07 (m, 4H), 2.88 (t, J = 7.72 Hz, 2H), 2.78 (s, 3H), 2.56 (t, J = 7.74 Hz, 2H), 2.32-2.26 (m, 2H), 1.21 (t, J = 7.22 Hz, 3H).

^{13}C NMR (101 MHz, CDCl$_3$) δ (ppm) 172.71, 167.41, 164.41, 161.44 (d, J = 245.72 Hz), 158.52 (d, J = 12.76 Hz), 152.37 (d, J = 248.11 Hz), 149.12 (d, J = 11.71 Hz), 130.83 (d, J = 8.78 Hz), 124.22, 123.39 (d, J = 9.57 Hz), 119.38 (d, J = 16.76 Hz), 115.42 (d, J = 21.53 Hz), 114.56, 110.05, 102.19 (d, J = 25.53 Hz), 65.72, 64.26, 60.35, 34.65, 29.01, 23.96, 15.68, 14.15.

^{19}F NMR (376 MHz, CDCl$_3$) δ (ppm) -116.23, -132.97.

HRMS (ESI) $[M+H]^+$ m/z: 463.1498 calculated for $C_{23}H_{25}F_2N_2O_4S$, found: 463.1496.

Synthesis of compound 1

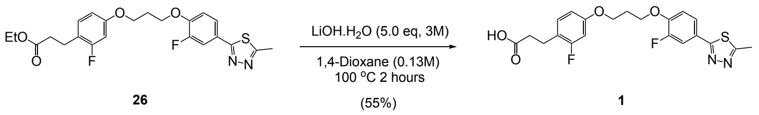

Figure S27: Synthesis scheme of **1**.

Compound **26**, (0.3 mmol, 1.0 eq.) was dissolved in 1,4-Dioxane (0.13 M) under vigorous stirring at room temperature. Then, LiOH x H2O (1.55 mmol, 5.0 eq.) was dissolved in water (3.0 M) and the resulting aqueous solution was added drop-wise to the previous one under vigorous stirring. After 15 minutes, the reaction mixture was heated to 100 °C for 2 h. Then, EtOAc and an aqueous solution of HCl (1.0 M) were added until phase separation was observed. The organic layers were washed with water, then combined and dried over $MgSO_4$. After evaporation of solvents, Et_2O was added at room temperature leading to the formation of a precipitate. The resulting solid was washed several times with Et_2O to obtain the pure product **1**. **Yield**: 55%. **Physical appearance**: White solid. **Melting point**: 155-156 °C.

^{1}H NMR (400 MHz, CD_3OD) δ (ppm) 7.72 (dd, J = 2.16, 11.8 Hz, 1H), 7.67-7.64 (m, 1H), 7.25 (t, J = 8.50 Hz, 1H), 7.17-7.13 (m, 1H), 6.71-6.75 (m, 2H), 4.32 (t, J = 6.2 Hz, 2H), 4.17 (t, J = 6.08 Hz, 2H), 2.85 (t, J = 7.60 Hz, 2H), 2.79 (s, 3H), 2.53 (t, J = 7.60 Hz, 2H), 2.32-2.26 (m, 2H).

^{13}C NMR (101 MHz, CD_3OD) δ (ppm) 176.62, 169.60, 167.43, 162.84 (d, J = 241.2 Hz), 160.27 (d, J = 10.96 Hz), 153.80 (d, J = 244.86 Hz), 150 (d, J = 10.05 Hz), 132.05 (d, J = 6.4 Hz), 125.78 (d, J = 2.74 Hz), 124.12 (d, J = 7.31 Hz), 120.68 (d, J = 16.45 Hz), 116.21, 115.89 (d, J = 20.1 Hz), 111.35 (d, J = 3.65 Hz), 103.06 (d, J = 25.58 Hz), 67.09, 65.65, 35.58, 30.16, 24.94, 15.33.

^{19}F NMR (376 MHz, CD_3OD) δ (ppm) -118.76, -135.13.

HRMS (ESI) $[M+H]^+$ m/z: 435.1185 calculated for $C_{21}H_{21}F_2N_2O_4S$, found: 435.1182.

Synthesis of compounds 2 and 3

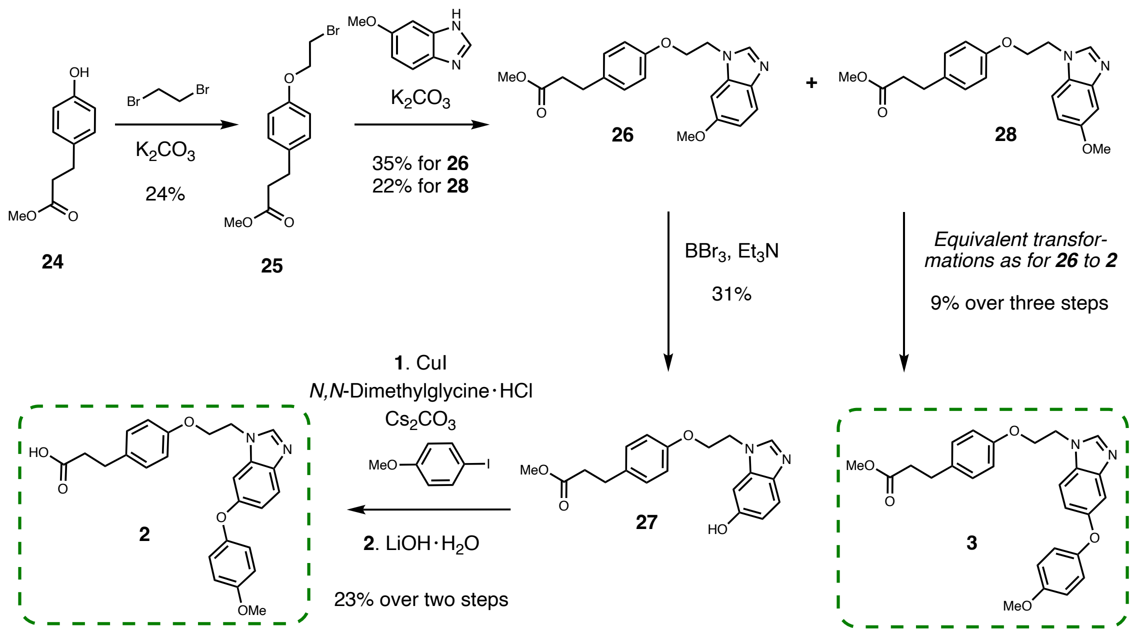

Figure S28: **Chemical synthesis of compounds 2 and 3**. For the synthesis of compounds **2** as well as the corresponding derivative **3**, the starting material used was commercial building block **24** and **33**. These compounds were synthesized through a sequential synthesis process involving five steps. The overall yield obtained for compound **2** was 0.6%, while for compound **3** and its derivative, the yield was 0.5%.

Synthesis of compound 29

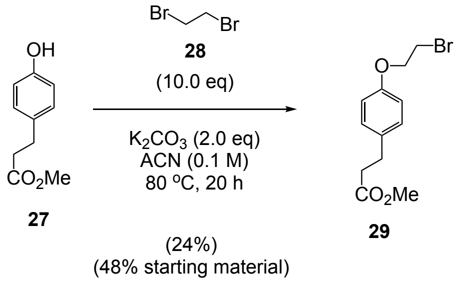

Figure S29: Synthesis scheme of **29**.

Compound **24**(11.1 mmol, 1.0 eq.) was dissolved in MeCN (0.1 M) under vigorous stirring at room temperature. Then, 1,2-Di-bromoethane (**28**, 111 mmol, 10 eq.) was added under vigorous stirring over ten minutes. Next, K_2CO_3 (22.2 mmol, 2.0 eq.) was added. The reaction mixture was stirred at room temperature for ten minutes before being heated at 80 °C for 20 h. The solid from the heterogeneous reaction mixture was discarded by filtration and rinsing with EtOAc. The solvent was evaporated and the crude material redissolved in EtOAc. The organic phase was washed with HCl 1.0 M, Brine, and water. The organic layers were combined and dried over $MgSO_4$. Solid discarded, by filtration, and the excess of the solvent was removed under reduced pressure to obtain the dried crude of the reaction, which was purified by flash chromatography. **System of elution**: from 100% Pentane to 20% EtOAc- 80% Pentane. **Yield**: 24% , 48% starting material remaining. **Physical appearance**: Pale yellow solid. **Melting point**: 55-56°C.

^{1}H NMR (400 MHz, $CDCl_3$) δ (ppm) 7.14-7.10 (m, 2H), 6.86-6.82 (m, 2H), 4.27 (t, J = 6.34 Hz, 2H), 3.66 (s, 3H), 3.62 (t, J = 6.34 Hz, 2H), 2.90 (t, J = 7.72 Hz, 2H), 2.60 (t, J = 7.72 Hz, 2H).

^{13}C NMR (101 MHz, $CDCl_3$) δ (ppm) 173.34, 156.58, 133.46, 129.36, 114.85, 67.94, 51.59, 35.91, 30.08, 29.14.

HRMS (ESI) $[M+Na]^+$ m/z: 309.0097 calculated for $C_{12}H_{15}BrNaO_3$, found: 309.0094.

Synthesis of compounds 31 and 32

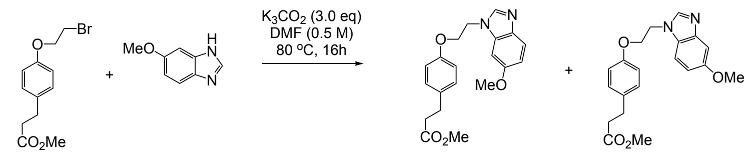

Figure S30: Synthesis scheme of **31** and **32**.

5-Methoxy-1H-benzimidazole (**30**) (5.3 mmol, 1.0 eq.) was dissolved in DMF (0.5 M) at room temperature. Once a homogeneous system was achieved through vigorous stirring, K_2CO_3 (16.0 mmol, 3.0 eq.) was added in one portion and the resulting heterogenic mixture was continuously stirred for 15 minutes at room temperature. Then, compound **29** (5.9 mmol, 1.1 eq.) was added in one portion and the reaction mixture was heated at 80 °C during overnight. The solid from the heterogeneous mixture was removed by filtration and solvents were evaporated. The dried crude material was redissolved in EtOAc. The organic phase was treated with HCl (1.0 M) and NaOH (1.0 M), followed by washing with Brine and water. The organic layers were combined and dried over $MgSO_4$. Evaporation of solvents delivered the crude material, which was purified by flash chromatography. **System of elution**: 1% MeOH 50% EtOAc 49% Pentane, 1% MeOH 80% EtOAc 19% Pentane, 1% MeOH 99% EtOAc. **Yield**: 35% compound **31**, **Yield**: 22%, compound **32**.

Regioisomer I (**26**): **Rf**: 0.18 at 1% MeOH 99% EtOAc **Physical appearance**: Brown solid. **Melting point**: 69-70 °C.
Regioisomer II (**28**): **Rf**: 0.18 at 1% MeOH 99% EtOAc. **Physical appearance**: White solid. **Melting point**: 124-125 °C.

Analytical data for regioisomer I (**26**):
^{1}H NMR (400 MHz, CDCl$_3$) δ (ppm) 7.93 (s, 1H), 7.69-7.67 (m, 1H), 7.10-7.07 (m, 2H), 6.94-6.91 (m, 2H), 6.79-6.75 (m, 2H), 4.50 (t, J = 5.32 Hz, 2H), 4.27 (t, J = 5.32 Hz, 2H), 3.88 (s, 3H), 3.65 (s, 3H), 2.87 (t, J = 7.72 Hz, 2H), 2.57 (t, J = 7.72 Hz, 2H).

^{13}C NMR (101 MHz, CDCl$_3$) δ (ppm) 173.21, 156.80, 156.34, 142.66, 138.16, 134.42, 133.52, 129.31, 120.80, 114.46, 111.38, 93.15, 66.18, 55.83, 51.50, 44.30, 35.79, 29.96.

HRMS (ESI) $[M+H]^+$ m/z: 355.1652 calculated for $C_{20}H_{23}N_2O_4$, found: 355.1655.

Synthesis of compound 33

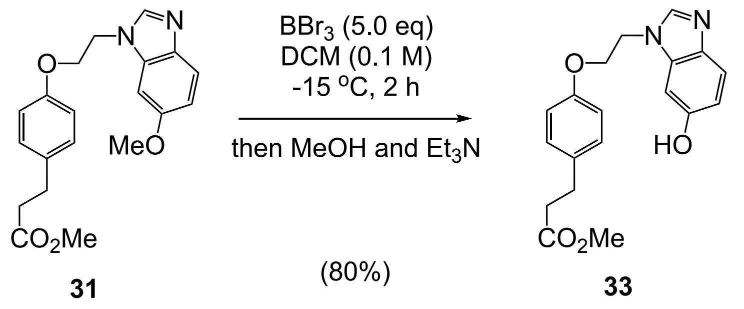

Figure S31: Synthesis scheme of **33**.

In a flamed dried round bottom flask, **25** (1.9 mmol, 1.0 eq.), was dissolved in dry DCM (0.1 M). The resulting solution was cooled to -15 °C and after 20 minutes, BBr3 (5.0 eq.) was added drop-wise as a 1.0 M solution in DCM to the mixture. The reaction mixture was stirred for two hours, from -15 °C up to -11°C. Then, the system was quenched with Methanol (5 mL), and the resulting mixture and warmed to room temperature., without external agent. Then, Et_3N was added until a precipitate was formed. Evaporation of solvents gave the crude material, which was redissolved in EtOAc. The precipitate was separated by filtration, rinsed with EtOAc and discarded. Solvents were removed from the organic layers, dried and subjected to flash chromatography for purification. **System of Elution**: 94% EtOAc-4% MeOH-2% Et3N. **Yield**: 80.**Rf**: 0.28 at 94% EtOAc-4% MeOH-2% Et3N. **Physical appereance**: White Solid. **Melting point**: 178-179 °C.
^{1}H NMR (400 MHz, CD_3OD) δ (ppm) 8.02 (s, 1H), 7.45 (d, J = 8.64 Hz, 1H), 7.05 (d, J = 8.64 Hz, 2H), 6.98 (d, J = 2.28 Hz, 1H), 6.80 (dd, J = 2.28, 8.64 Hz, 1H), 6.76 (d, J = 8.64 Hz, 2H), 4.53 (t, J = 5.06 Hz, 2H), 4.26 (t, J = 5.06 Hz, 2H), 3.60 (s, 3H), 2.80 (t, J = 7.6 Hz, 2H), 2.54 (t, J = 7.60 Hz, 2H).

^{13}C NMR (101 MHz, CD_3OD) δ (ppm) 175.15, 158.21, 155.65, 143.98, 137.62, 135.99, 134.69, 130.35, 120.41, 115.63, 113.37, 96.77, 67.62, 52.00, 45.52, 36.78, 31.06.

HRMS (ESI) $[M+H]^+$ m/z: 341.1496 calculated for $C_{19}H_{21}N_2O_4$, found: 341.1492.

Synthesis of compound 35

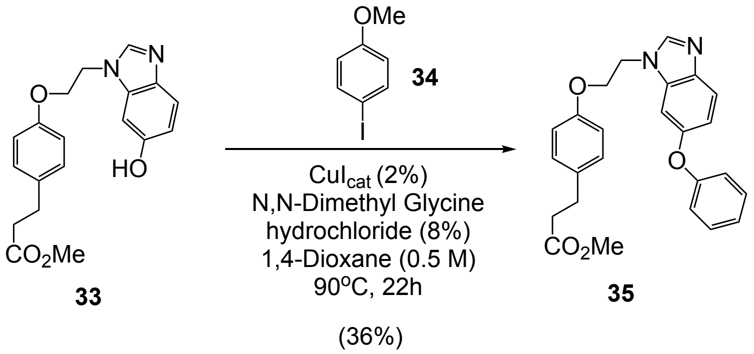

Figure S32: Synthesis scheme of **35**.

Compound (**29**) (1.5 mmol, 1.2 eq.), 4-Iodoanisole (1.1 mmol, 1.0 eq.), Cs_2CO_3 (2.2 mmol, 2.0 eq.), N,N-Dimethyl-Glycine hydrochloride (0.1 mmol, 0.08 eq.) and CuI (0.02 mmol, 0.02 eq.) were added to a flamed dried Schlenk tube. Then, dried 1,4-Dioxane (0.5 M) was added. The reaction mixture was heated up to 90 °C for 22 hours. Then, the solid from the resulting heterogeneous reaction mixture was separated by filtration, rinsed with EtOAc and discarded. The organic layers were treated with HCl (1.0 M) and NaOH (1.0 M), and washed with brine and water. The organic layers were combined and dried over $MgSO_4$. Evaporation of solvents delivered the crude material, which was purified by flash chromatography. **System of elution**: 4% MeOH, 2% Et3N, 94% MeOH. **Yield**: 36%. **Rf**: 0.48 at 4% MeOH, 2% Et_3N, 94% EtOAc. **Physical appereance**: Yellow solid. Reference for the conducted Ulmann reaction [46]

^{1}H NMR (400 MHz, CDCl$_3$) δ (ppm) 7.98 (s, 1H), 7.71 (d, J = 8.84 Hz, 1H), 7.09-7.05 (m, 2H), 7.02 (d, J = 2.28 Hz, 1H), 7.00-6.97 (m, 3H),6.90- 6.86 (m, 2H), 6.73-6.70 (m, 2H), 4.44 (t, J = 5.2 Hz, 2H), 4.21 (t, J = 5.08 Hz, 2H), 3.81 (s, 3H), 3.65 (s, 3H), 2.86 (t, J = 7.72 Hz, 2H), 2.57 (t, J = 7.72 Hz, 2H).

^{13}C NMR (101 MHz, CDCl$_3$) δ (ppm) 173.23, 156.33, 154.89, 151.27, 143.54, 139.65, 134.40, 133.58, 129.33, 121.07, 120.12, 114.86, 114.48, 114.37, 98.91, 66.81, 55.66, 51.58, 44.47, 35.86, 30.03.

HRMS (ESI) $[M+H]^+$ m/z: 447.1914 calculated for $C_{26}H_{27}N_2O_5$, found: 447.1916.

Syntheis of Compound 2

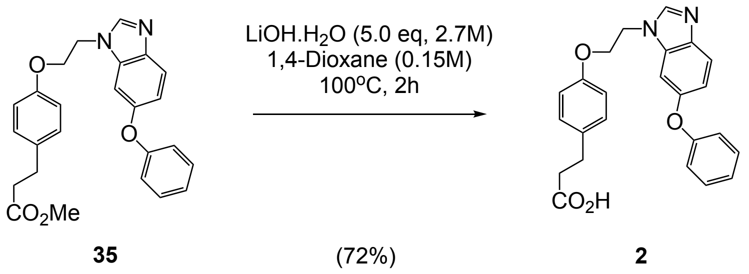

Figure S33: Synthesis scheme of **2**.

Compound (**30**), 0.5 mmol, 1.0 eq.) was dissolved in 1,4-Dioxane (0.15 M) at room temperature. Then, LiOH x H_2O (2.5 mmol, 5.0 eq.) was added as an aqueous solution (2.7 M) under vigorous stirring at room temperature for five minutes. Next, the reaction mixture was heated up to 100 °C for two hours. The system of the reaction was cold down up to room temperature, and To quench the reaction, an aqueous solution (pH4) was added carefully under vigorous stirring. Then, EtOAc was added until two heterogeneous phases were separated. Several extractions were performed using an aqueous solution (pH4), and the organic layers were washed with brine and water. The organic layers were combined and dried over $MgSO_4$. Evaporation of the solvents gave the crude material, which was purified by flash chromatography. **System of Elution**: 98% EtOAc - 1% MeOH - 1% AcOH, **Rf**: 0.52 at 98% EtOAc - 1% MeOH - 1% AcOH. **Yield**: 72%. **Physical appearance**: White solid. **Melting point**: 157-158 °C.

^{1}H NMR (400 MHz, THF-d8) δ (ppm) 7.95 (s, 1H), 7.56 (d, J = 8.88 Hz, 1H), 7.17 (d, J = 2.28 Hz, 1H), 7.09-7.05 (m, 2H), 6.95-6.91 (m, 2H), 6.88-6.84 (m, 3H), 6.76-6.72 (m, 2H), 4.51 (t, J = 5.2 Hz, 2H), 4.22 (t, J = 5.08 Hz, 2H), 3.75 (s, 3H), 2.78 (t, J = 7.72 Hz, 2H), 2.68 (t, J = 7.86 Hz, 2H).

^{13}C NMR (125 MHz, THF-d8) δ (ppm) 174.04, 157.97, 156.91, 155.57, 153.04, 144.83, 141.60, 136.07, 135.07, 130.15, 121.58, 120.66, 115.73, 115.43, 114.71, 100.62, 55.96, 45.17, 36.50, 31.15. One signal is overlapped with the deuterated solvent THF-d8 67.7 ppm (quintet).

HRMS (ESI) $[M+H]^+$ m/z: 433.1758 calculated for $C_{25}H_{25}N_2O_5$, found: 433.1766.

Synthesis of Compound 32

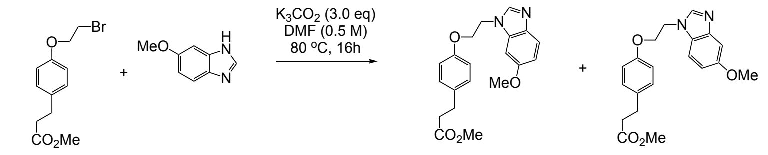

Figure S34: Synthesis scheme of **32**.

5-Methoxy-1H-benzimidazole (**30**) (5.3 mmol, 1.0 eq.) was dissolved in DMF (0.5 M) at room temperature. Vigorous stirring resulted in a homogeneous mixture. Next, K_2CO_3 (16.0 mmol, 3.0 eq.) was added in one portion and the resulting mixture was continuously stirred for 15 minutes at room temperature. At that point, Bromoalkane derivate **29** (5.9 mmol, 1.1 eq.) was added in one portion and the reaction mixture was heated at 80 °C during overnight. The solid from the heterogeneous reaction mixture was removed through filtration, rinsing with Ethyl acetate. Evaporation of the solvents of the organic layer delivered the crude material, which was redissolved in EtOAc, then trated with aqueous Solutions of HCl (1.0 M) and NaOH (1.0 M) were added, followed by the washing with Brine and water. The organic layers were combined, dried over $MgSO_4$. Evaporation of solvents delivered the crude material, which was purified by flash chromatography. **System of elution**: 1% MeOH 50% EtOAc 49% Pentane, 1% MeOH 80% EtOAc 19% Pentane, 1% MeOH 99% EtOAc. **Yield**: 35% compound **31**, **Yield**: 22%, compound **32**. **Melting point**: 124-125 °C, **Rf**: 0.18. 1% MeOH 99% EtOAc. **Physical appearance**: White solid.

^{1}H NMR (400 MHz, $CDCl_3$) δ (ppm) 7.95 (s, 1H), 7.33 (d, J = 8.6 Hz, 1H), 7.27 (d, J = 2.28 Hz, 1H), 7.09-7.05 (m, 2H), 6.96 (dd, J = 2.28, 8.6 Hz, 1H), 6.76-6.73 (m, 2H), 4.49 (t, J = 5.20 Hz, 2H), 4.24 (t, J = 5.18 Hz, 2H), 3.85 (s, 3H), 3.64 (s, 3H), 2.86 (t, J = 7.74 Hz, 2H), 2.56 (t, J = 7.74 Hz, 2H).

^{13}C NMR (101 MHz, $CDCl_3$) δ (ppm) 173.25, 156.37, 156.14, 144.54, 143.62, 133.53, 129.31, 128.39, 114.49, 113.17, 109.85, 102.39, 66.24, 55.76, 51.54, 44.55, 35.82, 29.99.

HRMS (ESI) $[M+H]^+$ m/z: 355.1652 calculated for $C_{20}H_{23}N_2O_4$, found: 355.1656.

Synthesis of compound 36

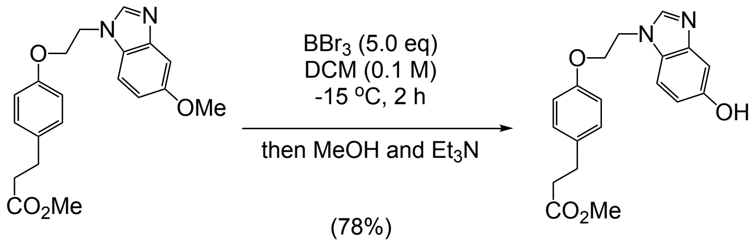

32 **36**

Figure S35: Synthesis scheme of **36**.

Compound **32** (1.1 mmol, 1.0 eq.) was dissolved in dry DCM (0.1 M). The solution was cooled to -15 °C over 20 minutes. Then, BBr_3 (5.0 eq.) was added drop-wise as a DCM solution (1.0 M). The reaction mixture was stirred at -15 °C up to -11 C during 2 h. The reaction was quenched with Methanol (5 mL), and the resulting mixture was removed from the cooling bath and warmed to room temperature, without external agent. Then, Et_3N was added until a precipitate was formed. Following for the evaporation of the solvents, EtOAc was added to redissolve the crude material and a precipitate is formed and removed by filtration, rinsed with EtOAc, Evaporation of the solvents of the organic layer delivered the crude material, which was was purified by flash chromatography. **System of Elution**: 94% EtOAc-4% MeOH-2% Et3N. **Yield**: 78%. **Rf**: 0.28 at 94% EtOAc-4% MeOH-2% Et3N. **Physical appereance**: White Solid. **Melting point**: 153-154°C.

^{1}H NMR (400 MHz, CD_3OD) δ (ppm) 8.08 (s, 1H), 7.43 (d, J = 8.60 Hz, 1H), 7.06-7.02 (m, 3H), 6.87 (dd, J = 2.28, 8.60 Hz, 1H), 6.76-6.72 (m, 2H), 4.55 (t, J = 4.94 Hz, 2H), 4.25 (t, J = 5.06 Hz, 2H), 3.60 (s, 3H), 2.79 (t, J = 7.60 Hz, 2H), 2.54 (t, J = 7.60 Hz, 2H).

^{13}C NMR (101 MHz, CD_3OD) δ (ppm) 175.13, 158.15, 154.88, 145.05, 144.72, 134.67, 130.35, 129.13, 115.60, 114.16, 111.84, 104.53, 67.63, 51.99, 45.71, 36.77, 31.04.

HRMS (ESI) $[M+H]^+$ m/z: 341.1496, calculated for $C_{19}H_{21}N_2O_4$, found: 341.1498.

Synthesis of compound 38

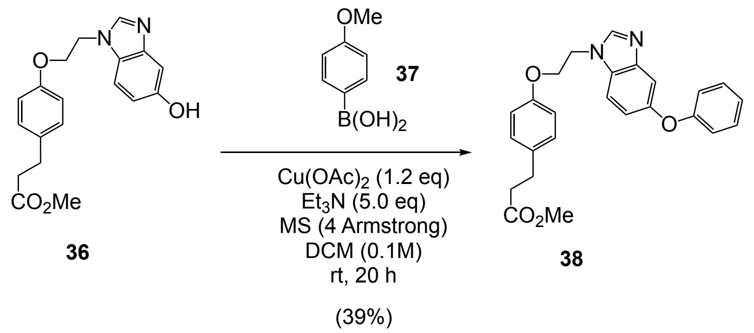

Figure S36: Synthesis scheme of **38**.

Under normal atmosphere, compound **36** (0.9 mmol, 1.0 eq.) was dissolved in DCM (0.1 M) under vigorous stirring. Then, 4-Methoxyphenylboronic acid (1.7 mmol, 2.0 eq.) and $Cu(OAc)_2$ (1.0 mmol, 1.2 eq.) were added to the reaction mixture. Then, activated MS 4 Armstrong (1.0 g) and NEt_3 (4.3 mmol, 5.0 eq.) were added sequentially. The resulting reaction mixture was stirred at room temperature for 20 hours. The reaction mixture was filtered through Celite and rinsed with DCM. The excess of the solvent was removed and was purified by flash chromatography. **System of elution**: 4% MeOH, 2% Et3N, 94% EtOAc. **Yield**: 39%. **Physical appereance**: Yellow solid. **Rf**: 0.48 at 4% MeOH, 2% Et3N, 94% EtOAc. **Melting point**: 100-101 °C. Reference for the conducted Chan-Lam reaction [47].

^{1}H NMR (400 MHz, $CDCl_3$) δ (ppm) 8.02 (s, 1H), 7.41-7.37 (m, 2H), 7.10- 7.03 (m, 3H), 6.98-6.94 (m, 2H), 6.88-6.83 (m, 2H), 6.78-6.74 (m, 2H), 4.52 (t, J = 5.2 Hz, 2H), 4.26 (t, J = 5.06 Hz, 2H), 3.79 (s, 3H), 3.64 (s, 3H), 2.87 (t, J = 7.72 Hz, 2H), 2.57 (t, J = 7.72 Hz, 2H).

^{13}C NMR (101 MHz, $CDCl_3$) δ (ppm) 173.25, 156.33, 155.41, 153.96, 151.54, 144.33, 133.59, 129.35, 119.80, 115.43, 114.76, 114.48, 109.97, 109.39, 66.15, 55.65, 51.57, 44.64, 35.82, 29.99.

HRMS (ESI) $[M+H]^+$ m/z: 447.1914 calculated for $C_{26}H_{27}N_2O_5$, found: 447.1919.

Synthesis of compound 3

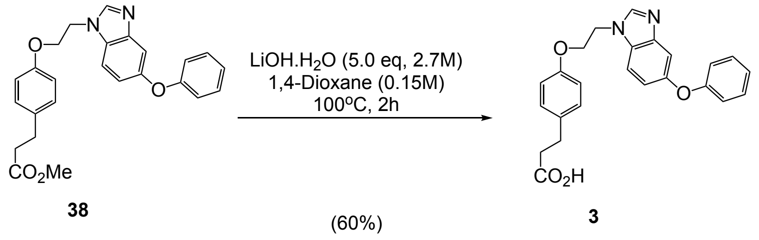

Figure S37: Synthesis scheme of **3**.

Compound **38** (0.3 mmol, 1.0 eq.) was dissolved in 1,4-Dioxane (0.15 M) at room temperature. Under vigorous stirring, LiOH x H_2O (1.5 mmol, 5.0 eq.) was added as an aqueous solution (2.7 M). The resulting reaction mixture was stirred at room temperature for five minutes, before being heated to 100 °C for two hours. The system was removed from teh heating source, and To quench the reaction, an aqueous solution (pH4) was added carefully under vigorous stirring, at room temperature. Then, EtOAc was added until two heterogeneous phases were separated. Several extractions were performed using an aqueous solution (pH4), and the organic layers were washed with brine and water. The organic layers were combined and dried over $MgSO_4$. Evaporation of the solvents gave the crude material, which was purified by flash chromatography. **System of Elution**: 98% EtOAc - 1% MeOH - 1% AcOH. **Yield**: 60%. **Physical appearance**: white solid. **Rf**: 0.52 at 98% EtOAc - 1% MeOH - 1% AcOH. **Melting point**: 126-127 °C.

^{1}H NMR (400 MHz, CD_3OD) δ (ppm) 8.28 (s, 1H), 7.64 (d, J = 8.84 Hz, 1H), 7.14 (d, J = 2.28 Hz, 1H), 7.10-7.05 (m, 3H), 6.96-6.89 (m, 4H), 6.80-6.76 (m, 2H), 4.66 (t, J = 4.94 Hz, 2H), 4.32 (t, J = 5.06 Hz, 2H), 3.78 (s, 3H), 2.81 (t, J = 7.60 Hz, 2H), 2.52 (t, J = 7.60 Hz, 2H).

^{13}C NMR (101 MHz, CD_3OD) δ (ppm)176.75, 158.09, 157.32, 156.29, 152.57, 145.80, 143.80, 135.02, 131.00, 130.38, 121.17, 116.70, 115.96, 115.59, 112.61, 108.03, 67.61, 56.08, 46.03, 36.93, 31.14.

HRMS (ESI) $[M+H]^+$ m/z: 433.1758 calculated for $C_{25}H_{25}N_2O_5$, found: 433.1766.

SI13.5 NMR Spectra

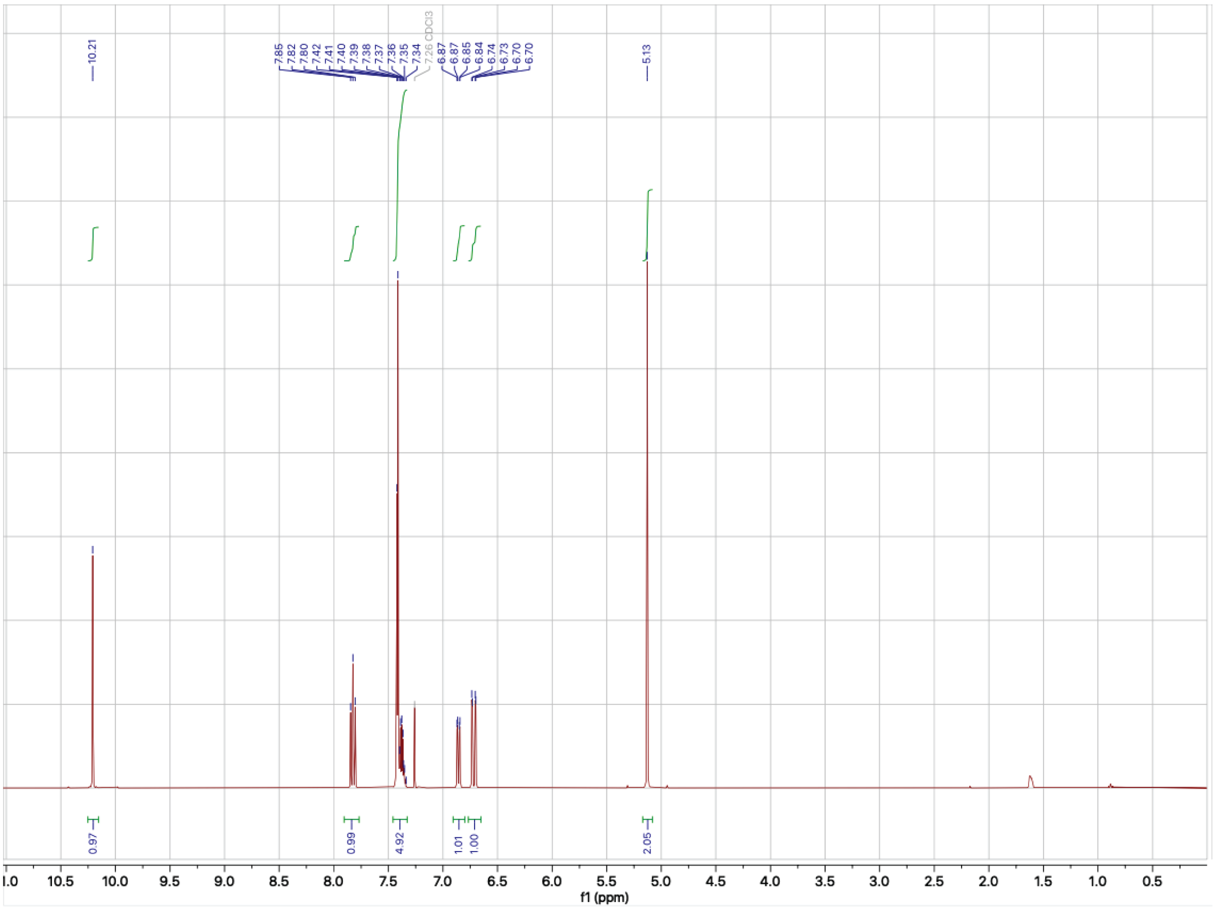

Figure S38: **14**, ^{1}H-NMR spectra.

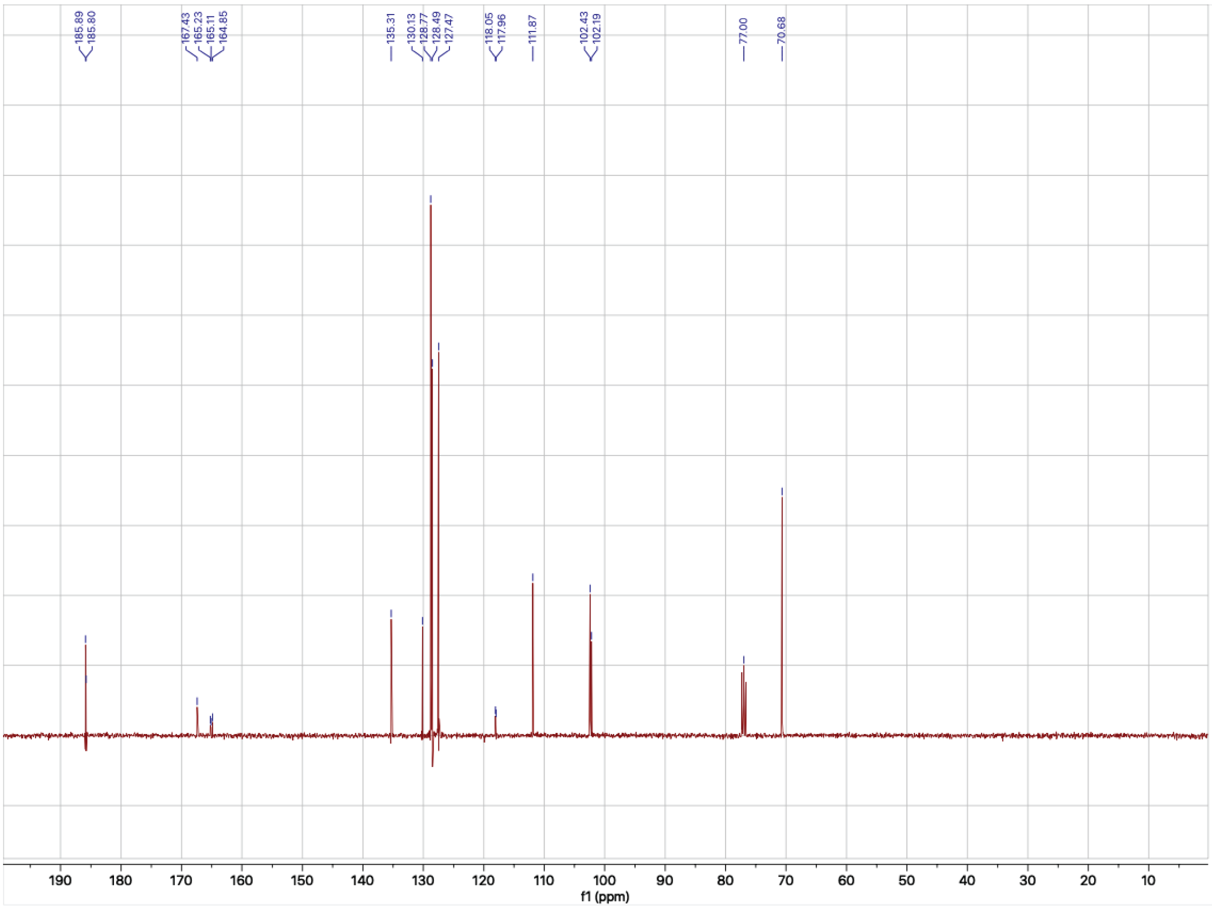

Figure S39: **14**, ^{13}C-NMR spectra.

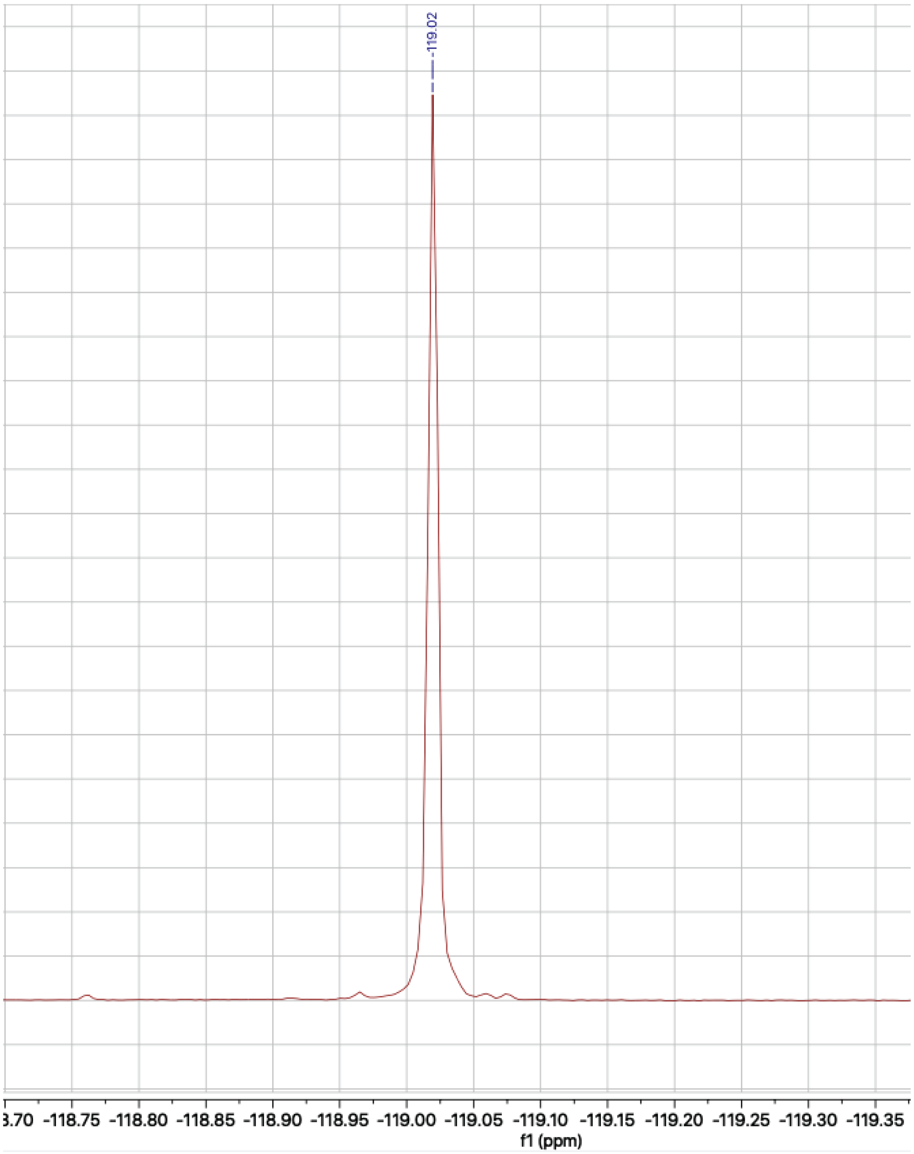

Figure S40: **14**, ^{19}F-NMR spectra.

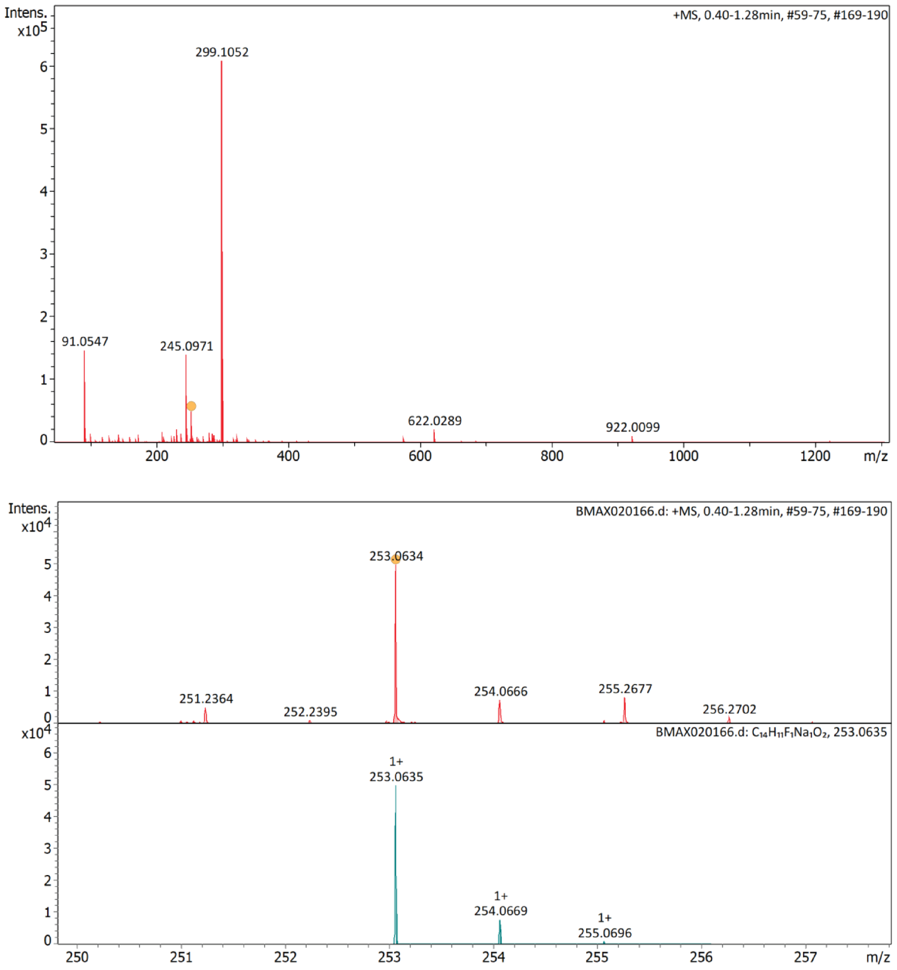

#	Ion Formula	Adduct	m/z	z	Meas. m/z	mSigma	N-Rule	err [mDa]	err [ppm]
1	C14H11FNaO2	M+Na	253.0635	1+	253.0634	3.6	ok	0.1	0.6

Figure S41: **14**, HRMS.

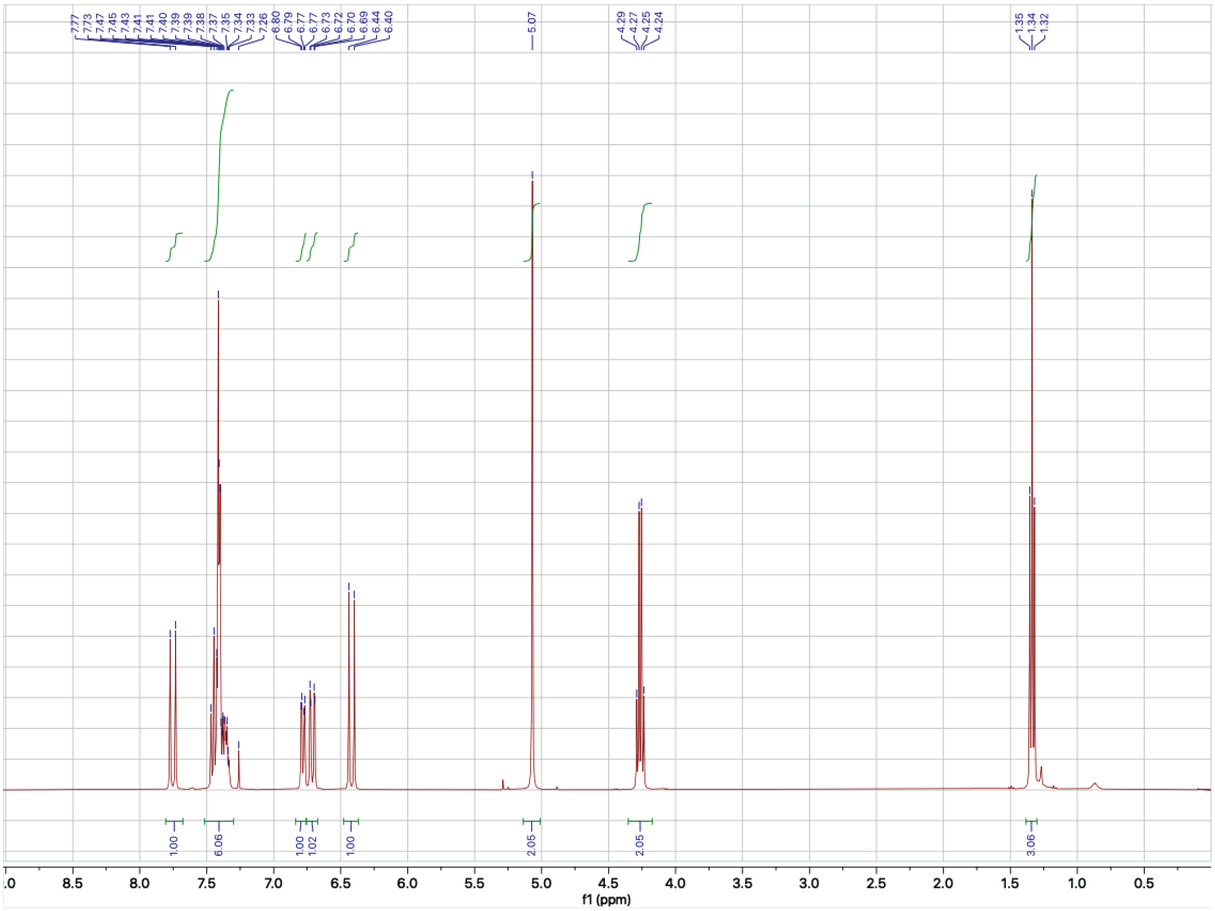

Figure S42: **16**, ^{1}H-NMR spectra.

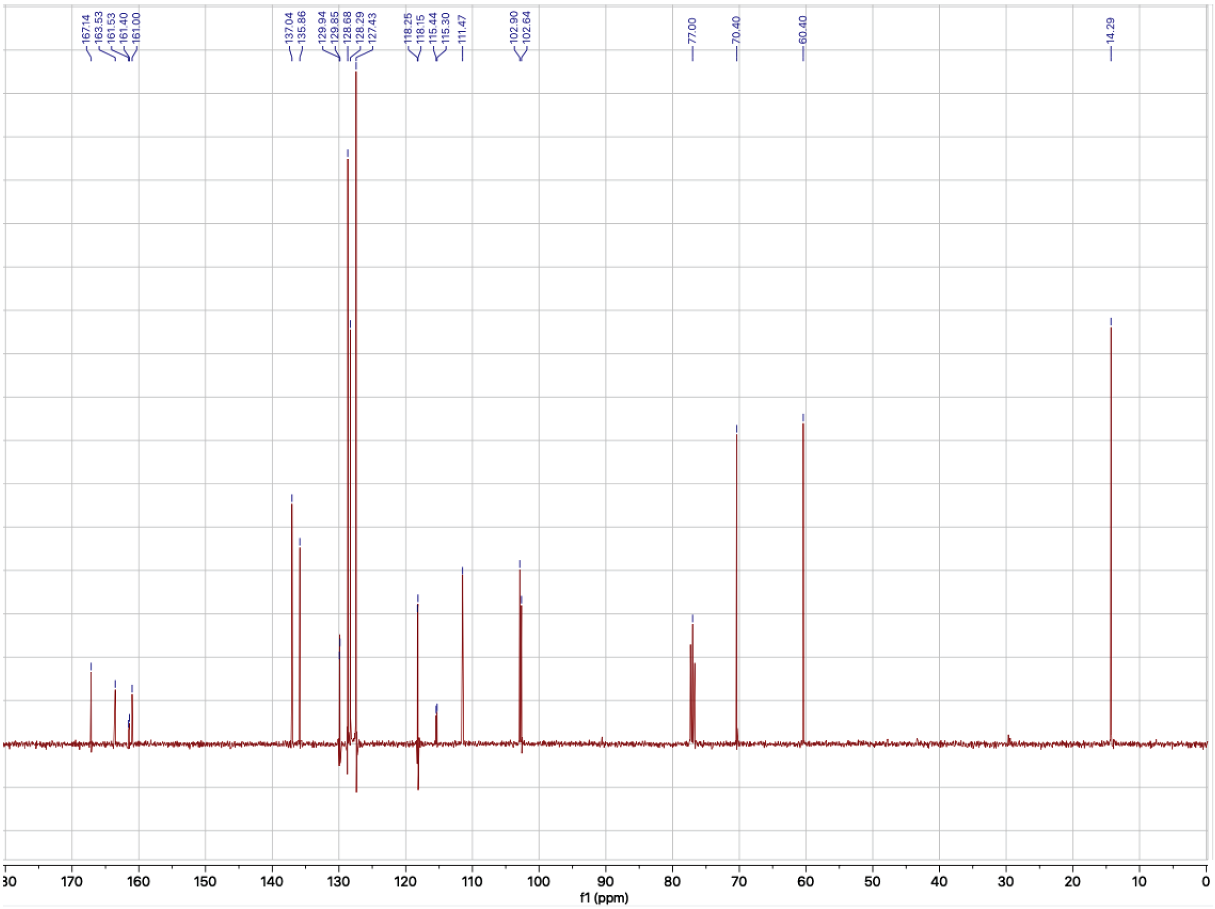

Figure S43: **16**, ^{13}C-NMR spectra.

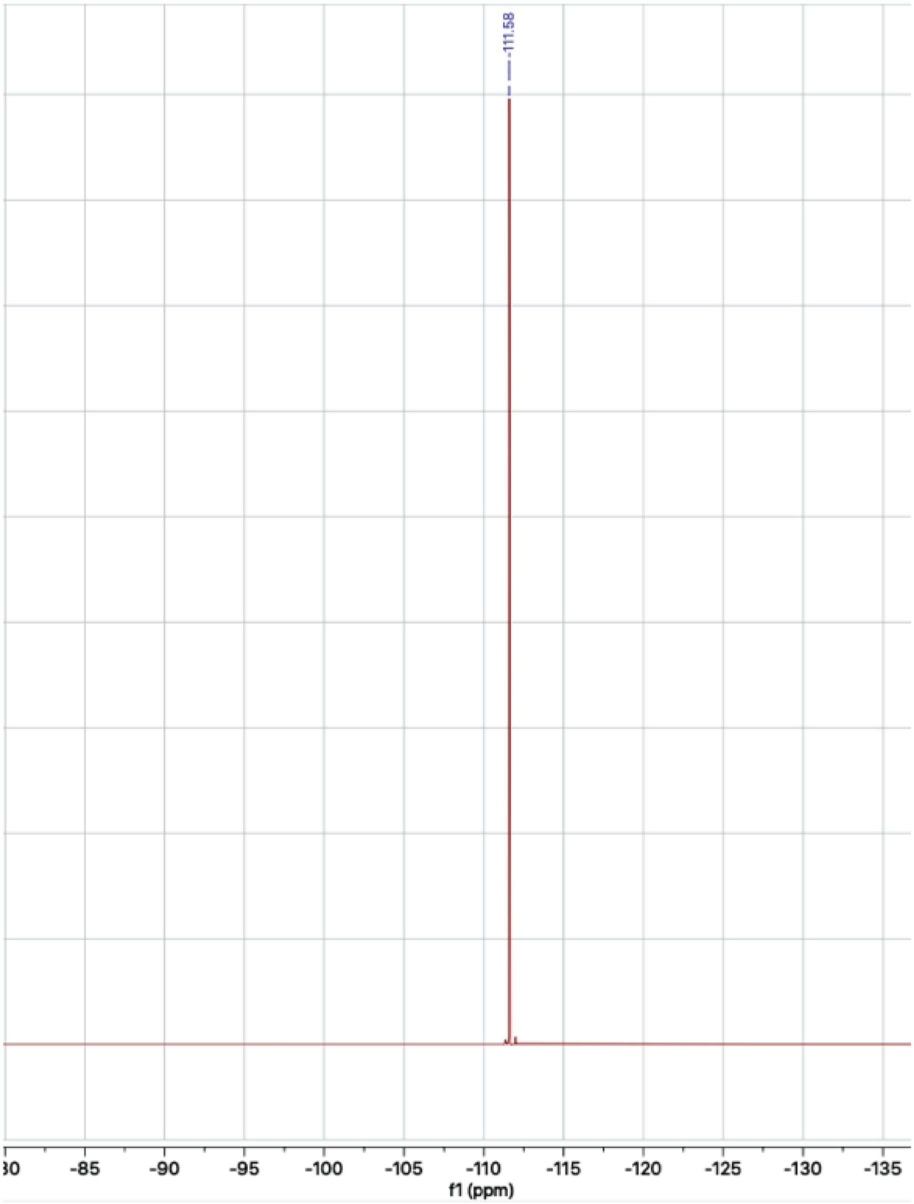

Figure S44: **16**, ^{19}F-NMR spectra.

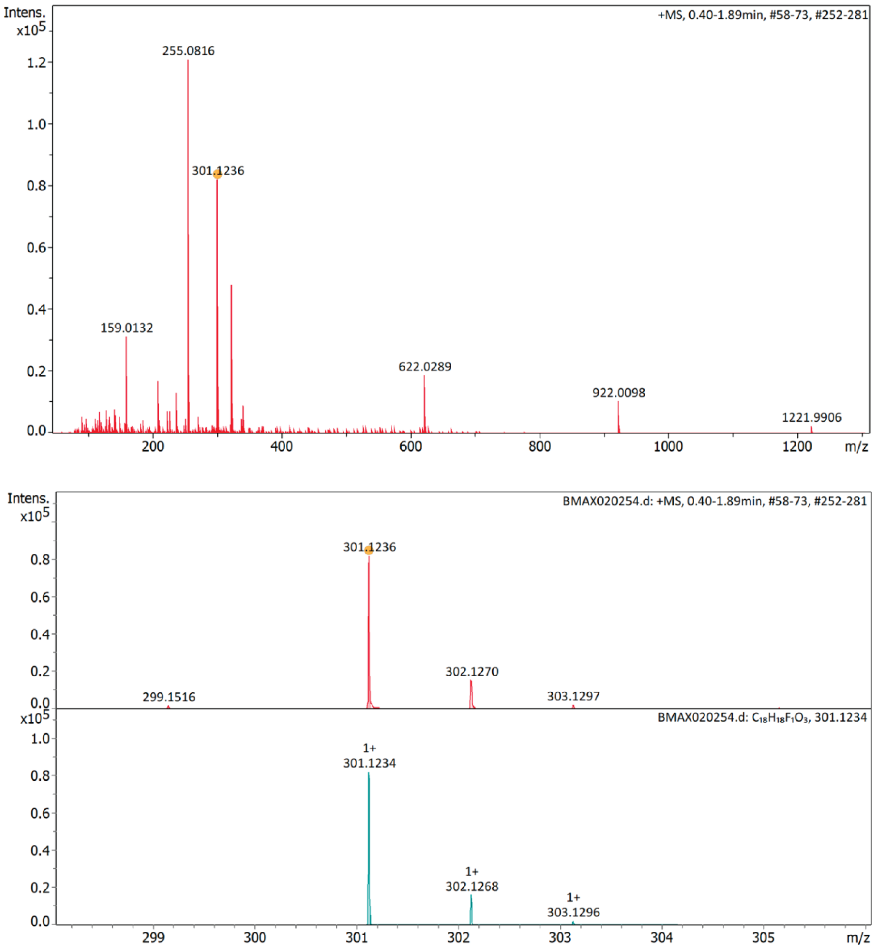

#	Ion Formula	Adduct	m/z	z	Meas. m/z	mSigma	N-Rule	err [mDa]	err [ppm]
1	C18H18FO3	M+H	301.1234	1+	301.1236	6.8	ok	-0.2	-0.5

Figure S45: **16**, HRMS.

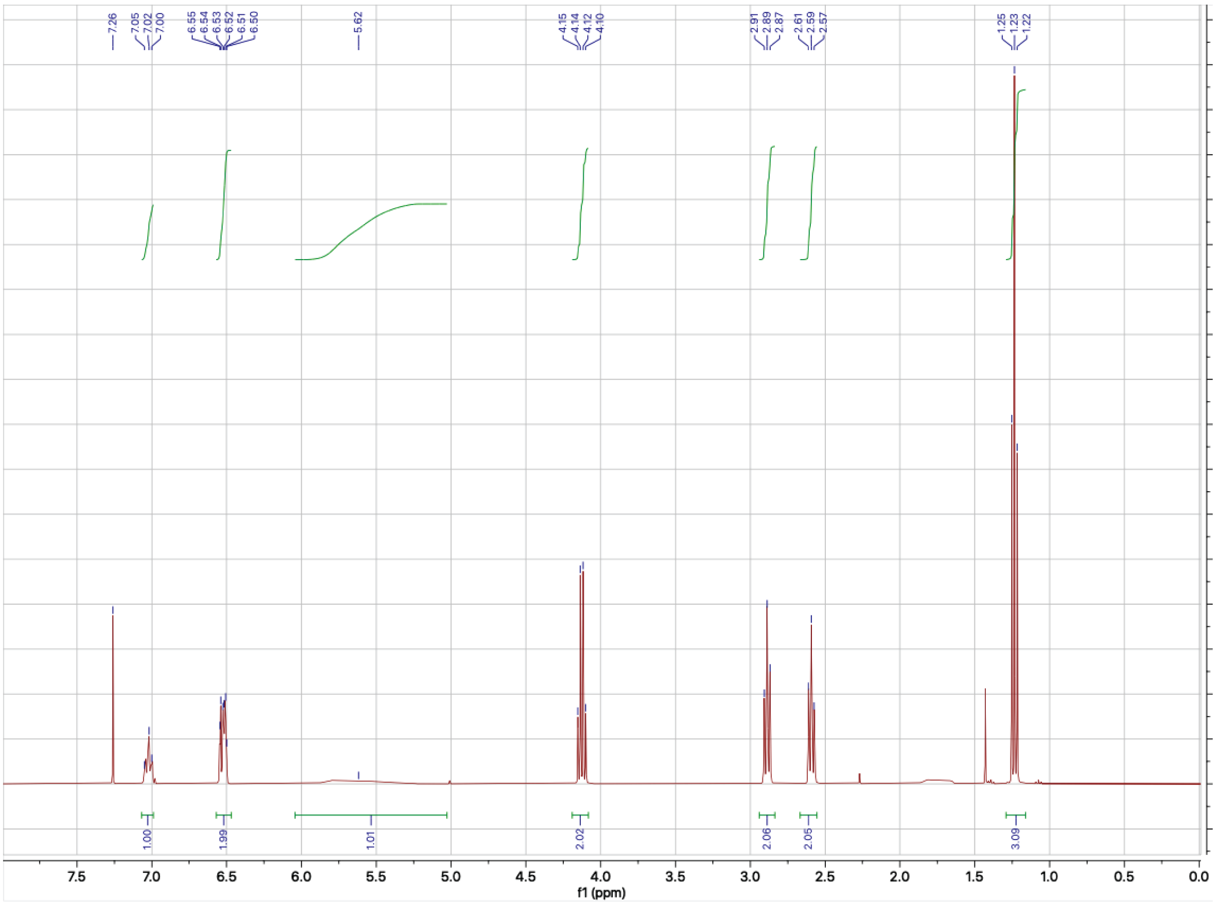

Figure S46: **17**, ^{1}H-NMR spectra.

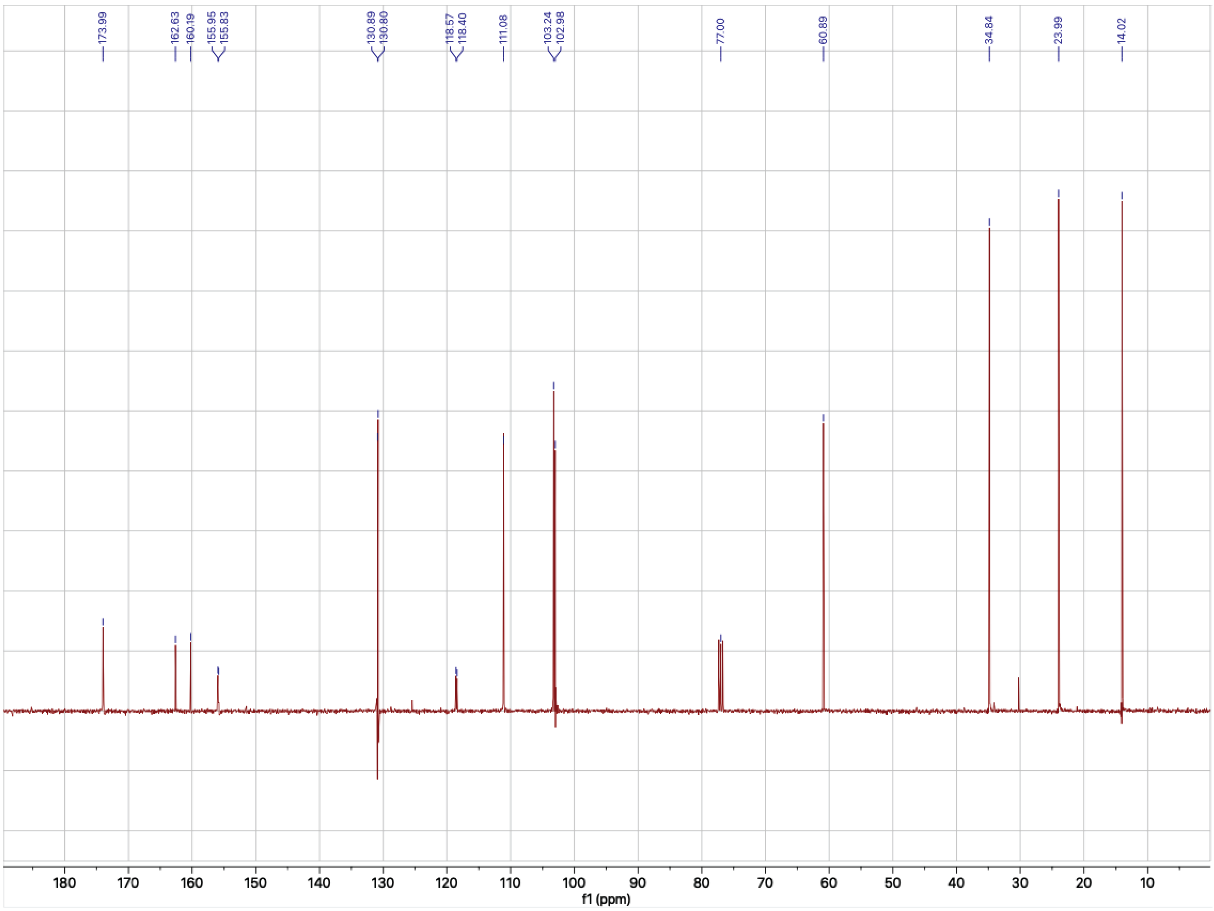

Figure S47: **17**, ^{13}C-NMR spectra.

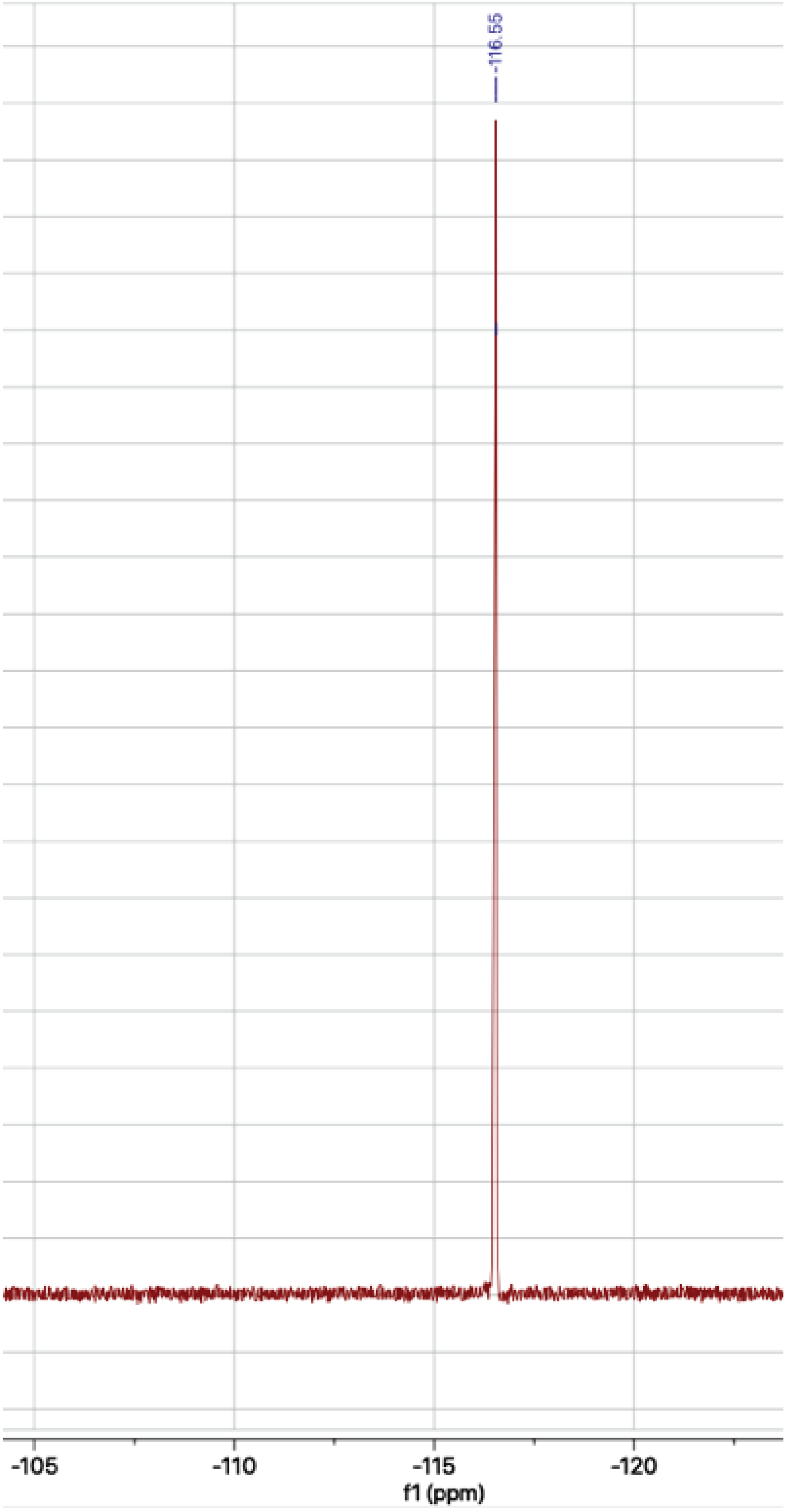

Figure S48: **17**, ^{19}F-NMR spectra.

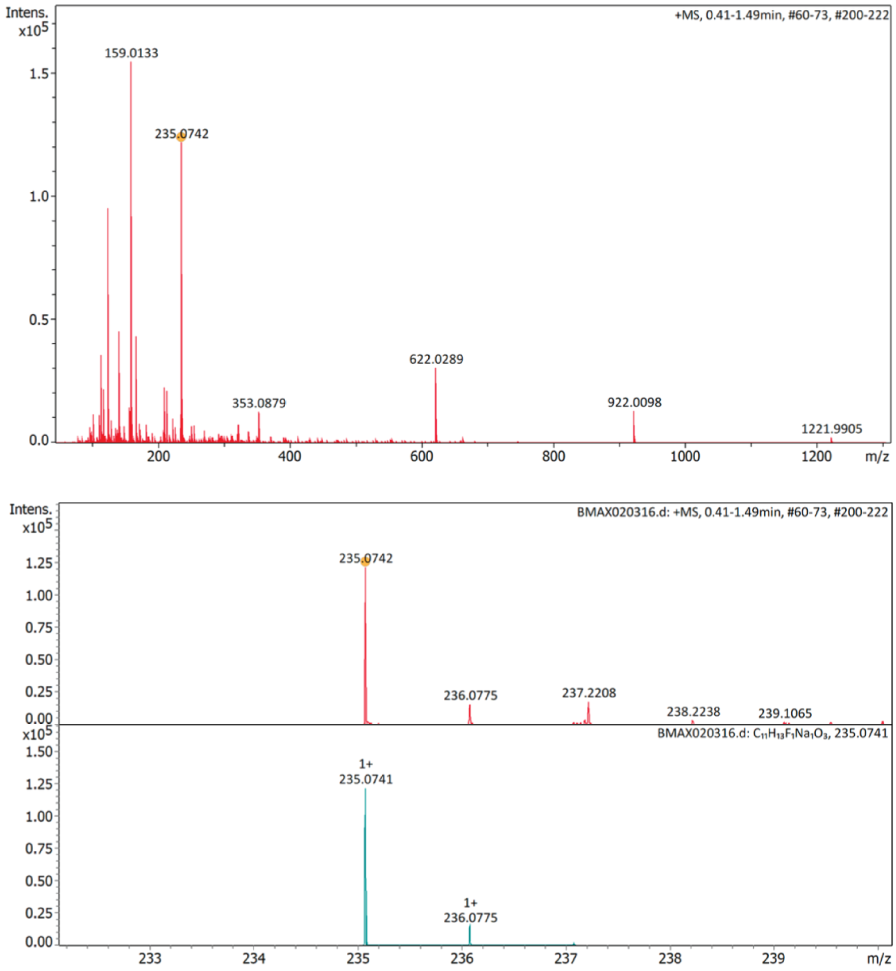

#	Ion Formula	Adduct	m/z	z	Meas. m/z	mSigma	N-Rule	err [mDa]	err [ppm]
1	C11H13FNaO3	M+Na	235.0741	1+	235.0742	3.2	ok	-0.2	-0.7

Figure S49: **17**, HRMS.

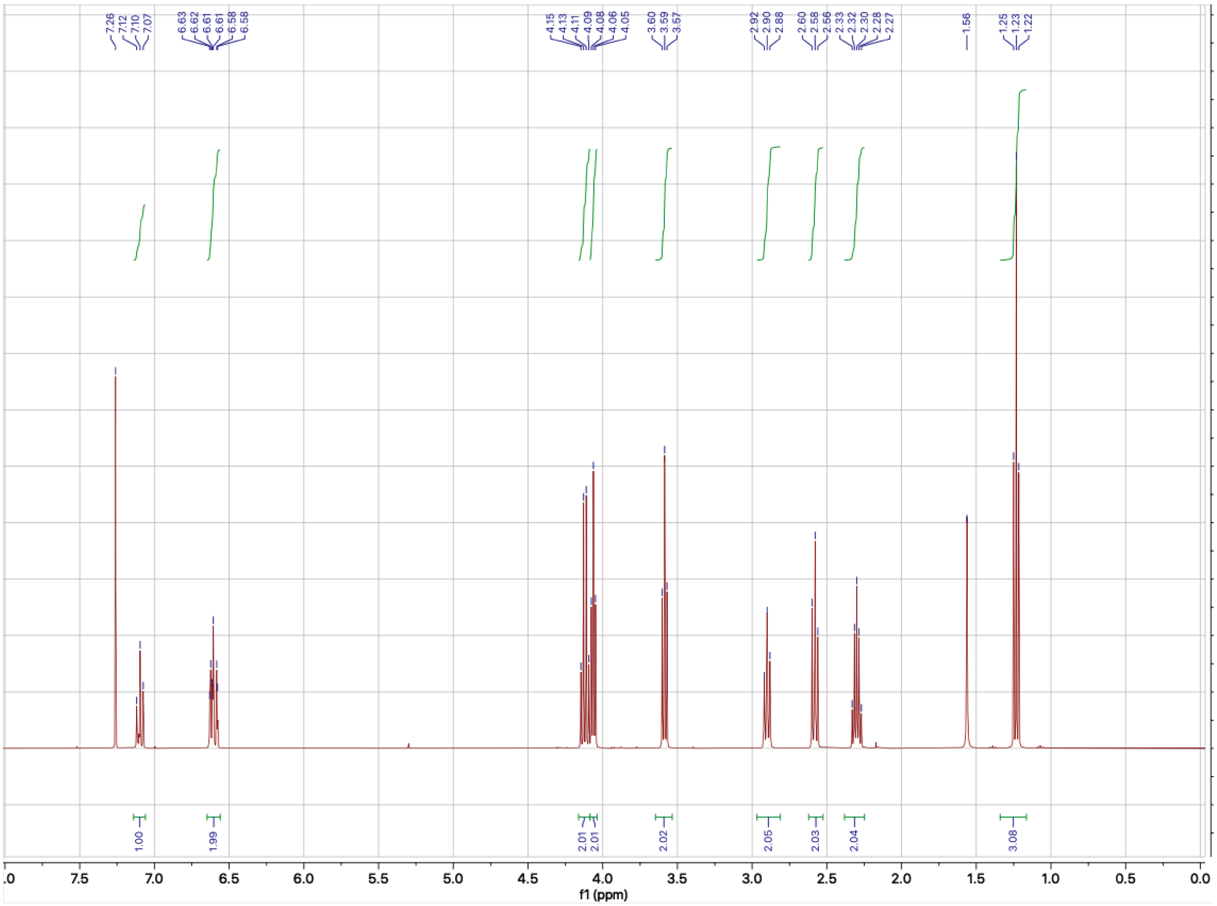

Figure S50: **19**, ^{1}H-NMR spectra.

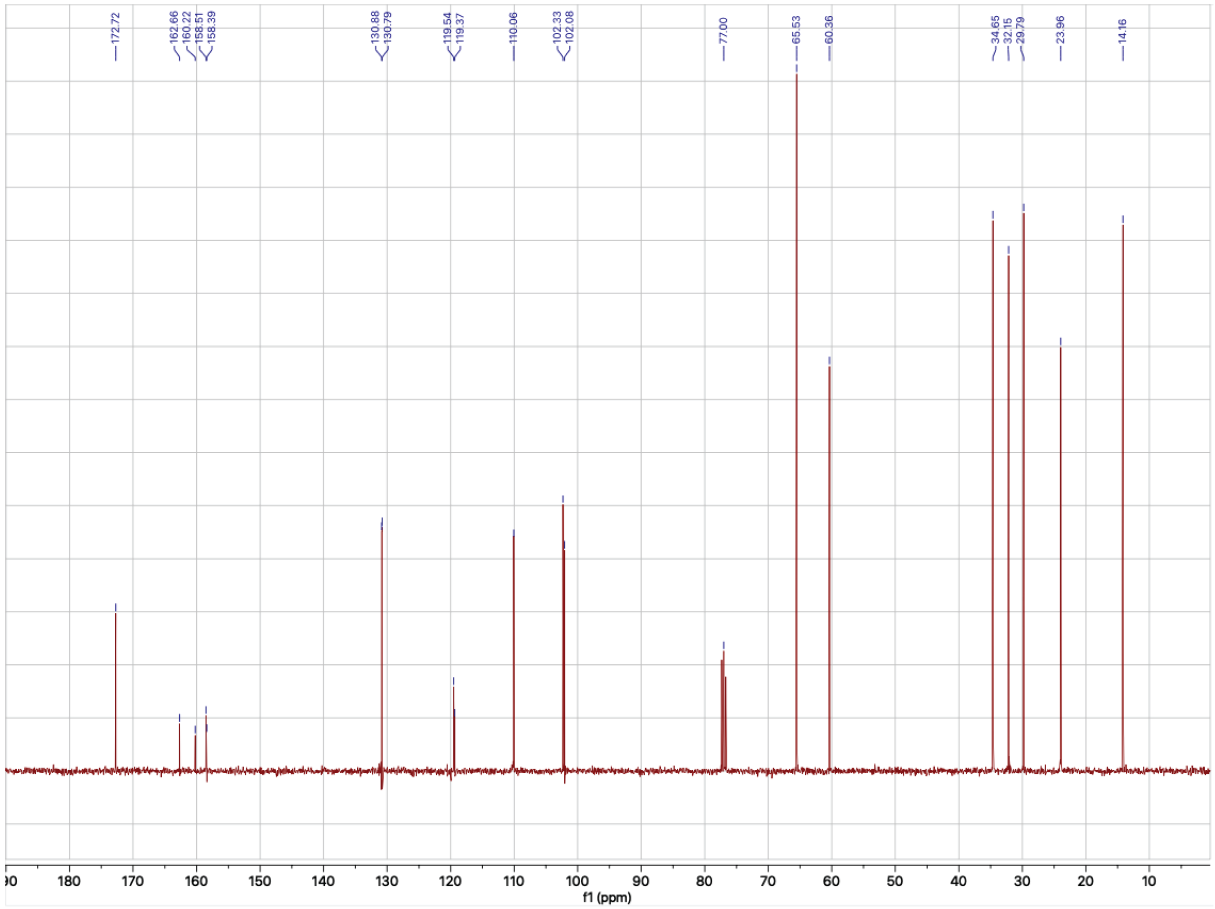

Figure S51: **19**, ^{13}C-NMR spectra.

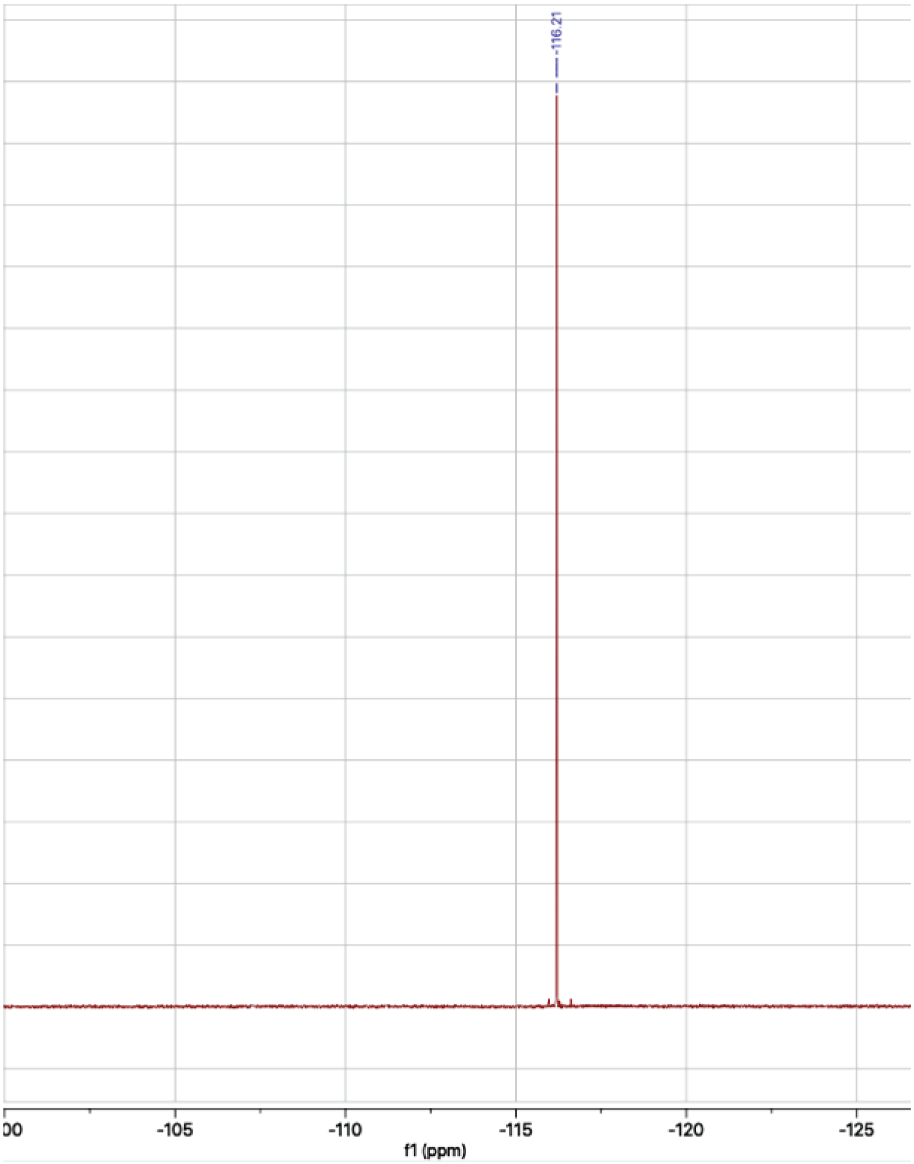

Figure S52: **19**, ^{19}F-NMR spectra.

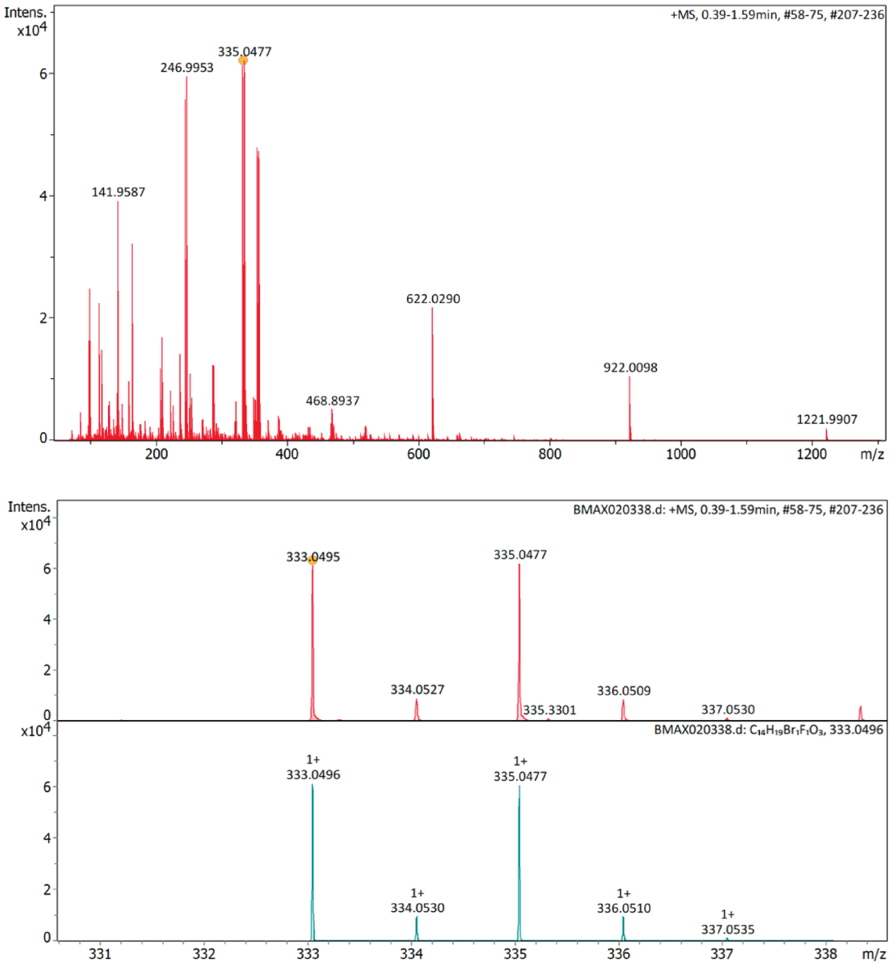

#	Ion Formula	Adduct	m/z	z	Meas. m/z	mSigma	N-Rule	err [mDa]	err [ppm]
1	C14H19BrFO3	M+H	333.0496	1+	333.0495	11.5	ok	0.1	0.4

Figure S53: **19**, HRMS.

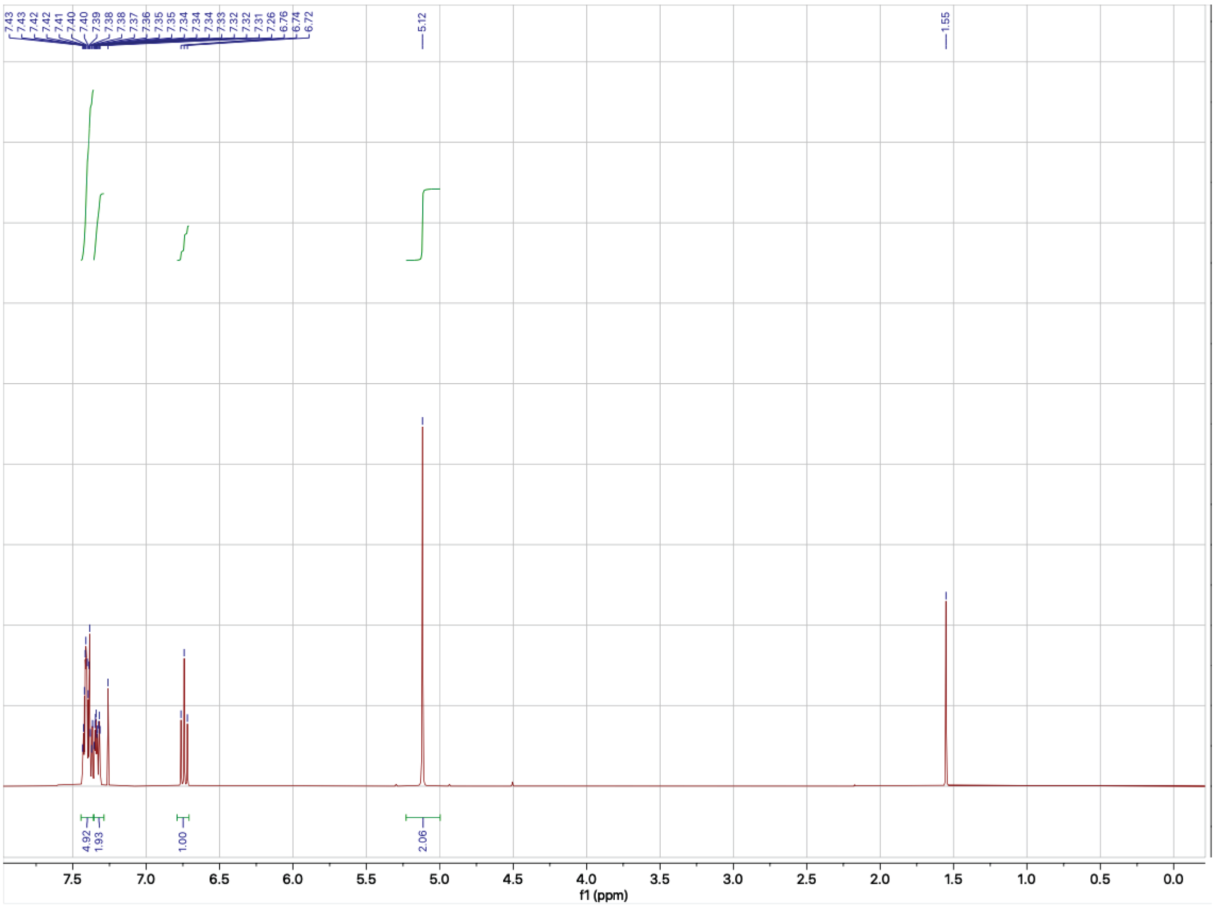

Figure S54: **21**, ^{1}H-NMR spectra.

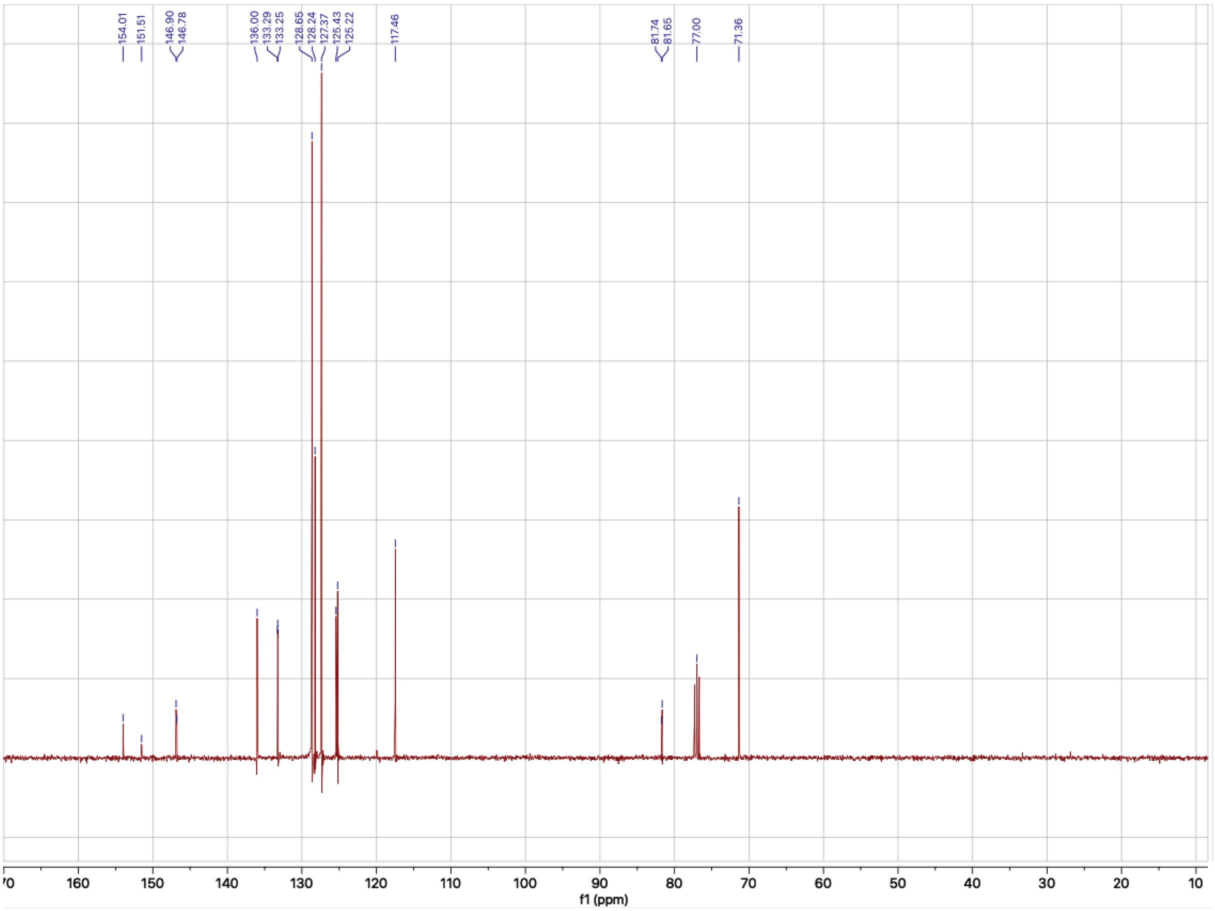

Figure S55: **21**, ^{13}C-NMR spectra.

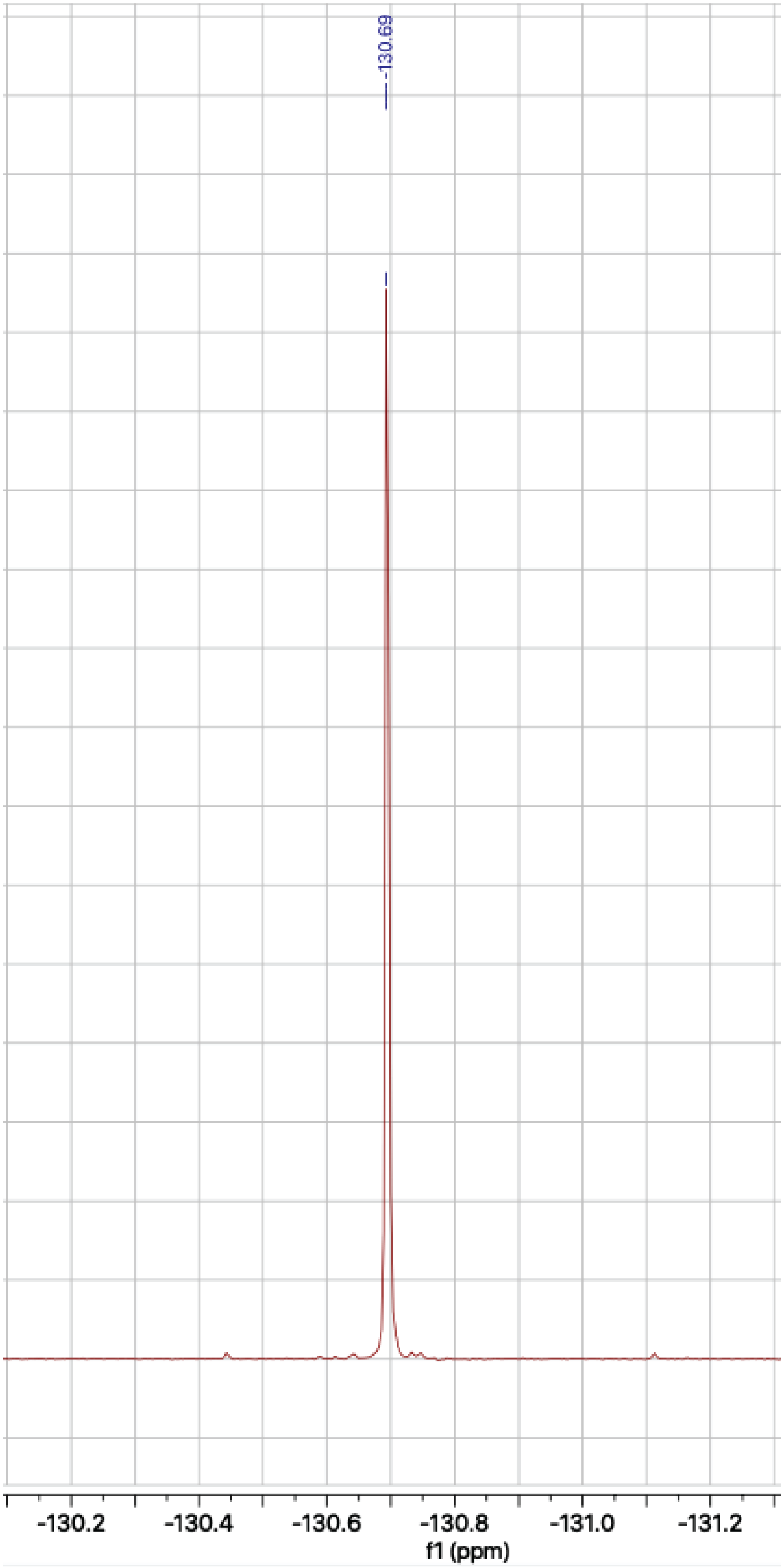

Figure S56: **21**, ^{19}F-NMR spectra.

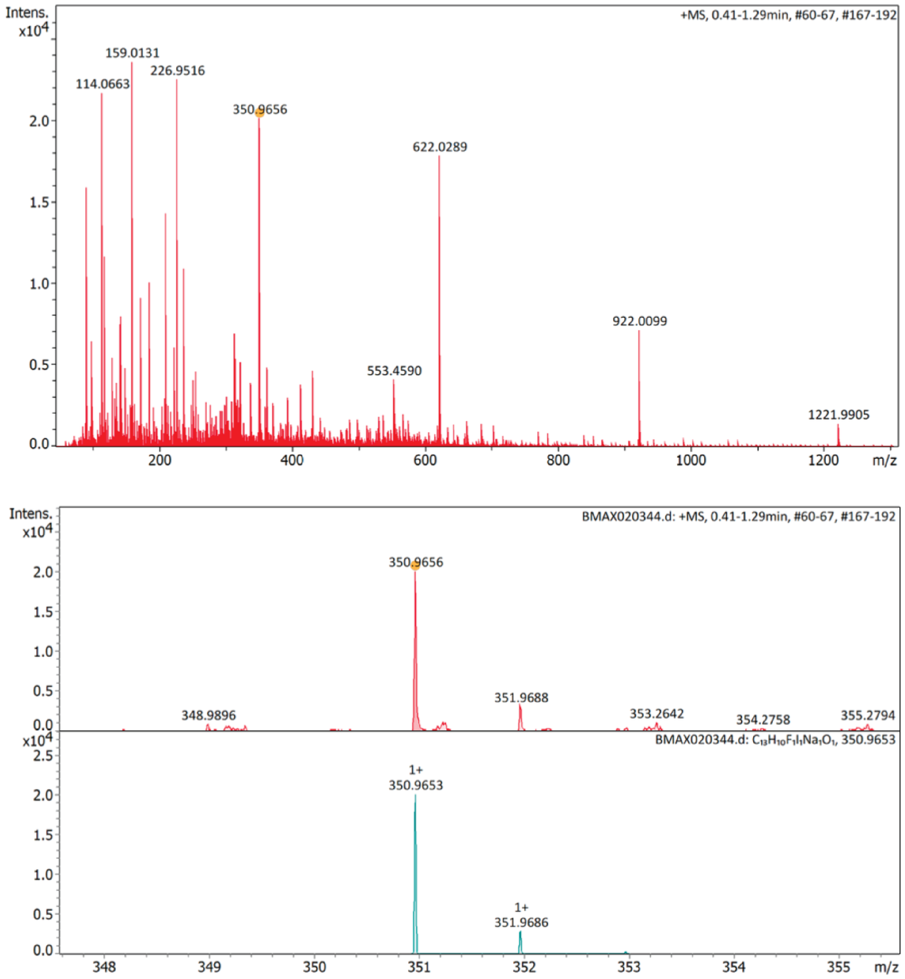

#	Ion Formula	Adduct	m/z	z	Meas. m/z	mSigma	N-Rule	err [mDa]	err [ppm]
1	C13H10FINaO	M+Na	350.9653	1+	350.9656	7.1	ok	-0.4	-1.0

Figure S57: **21**, HRMS.

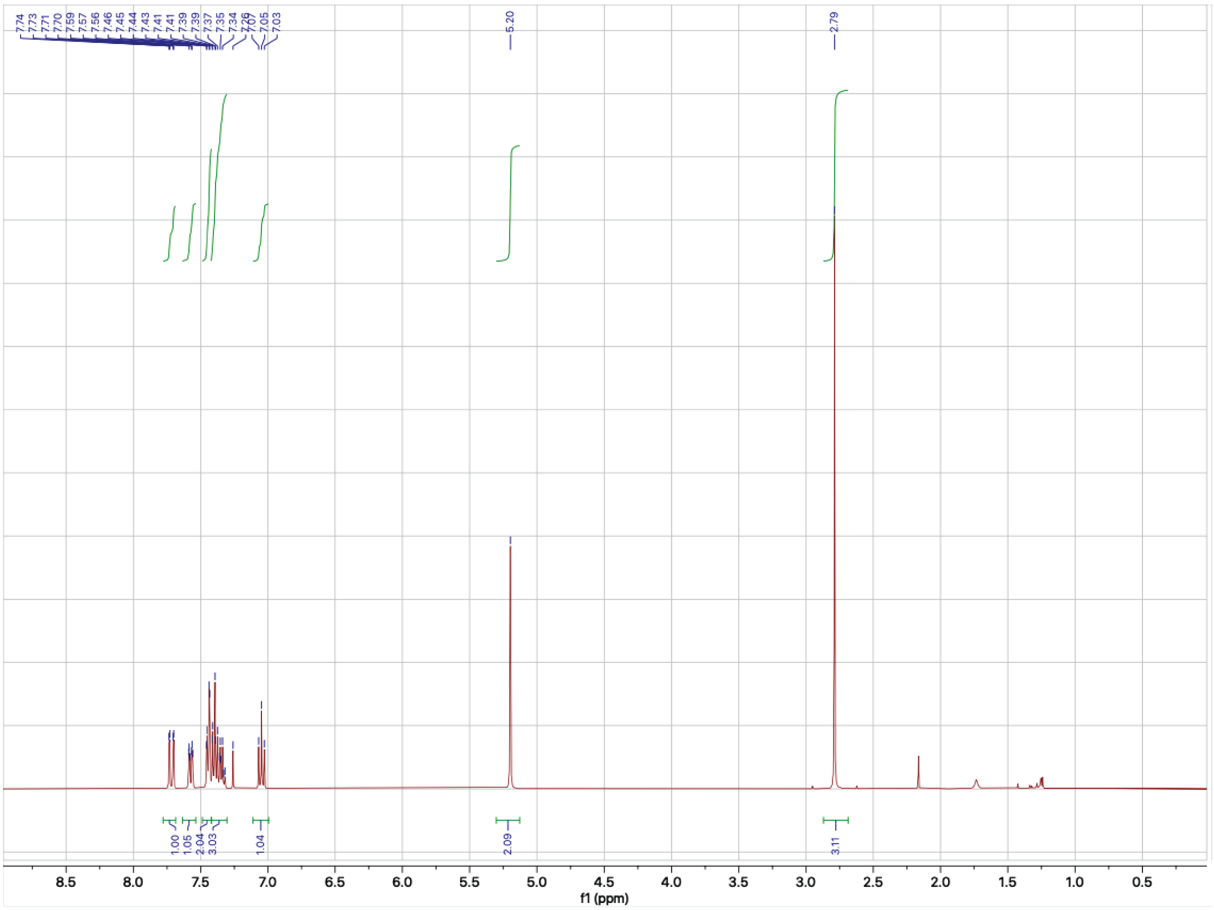

Figure S58: **24**, ^{1}H-NMR spectra.

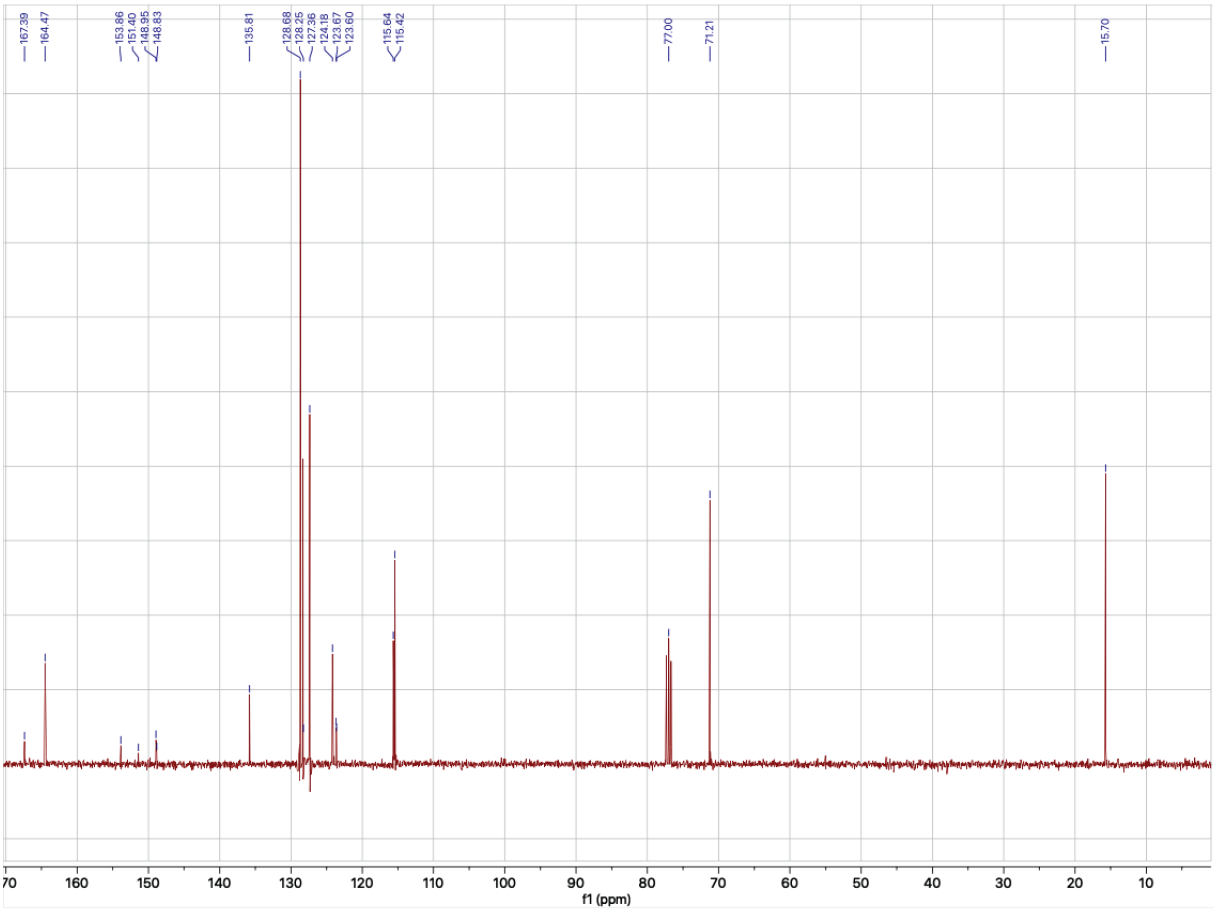

Figure S59: **24**, ^{13}C-NMR spectra.

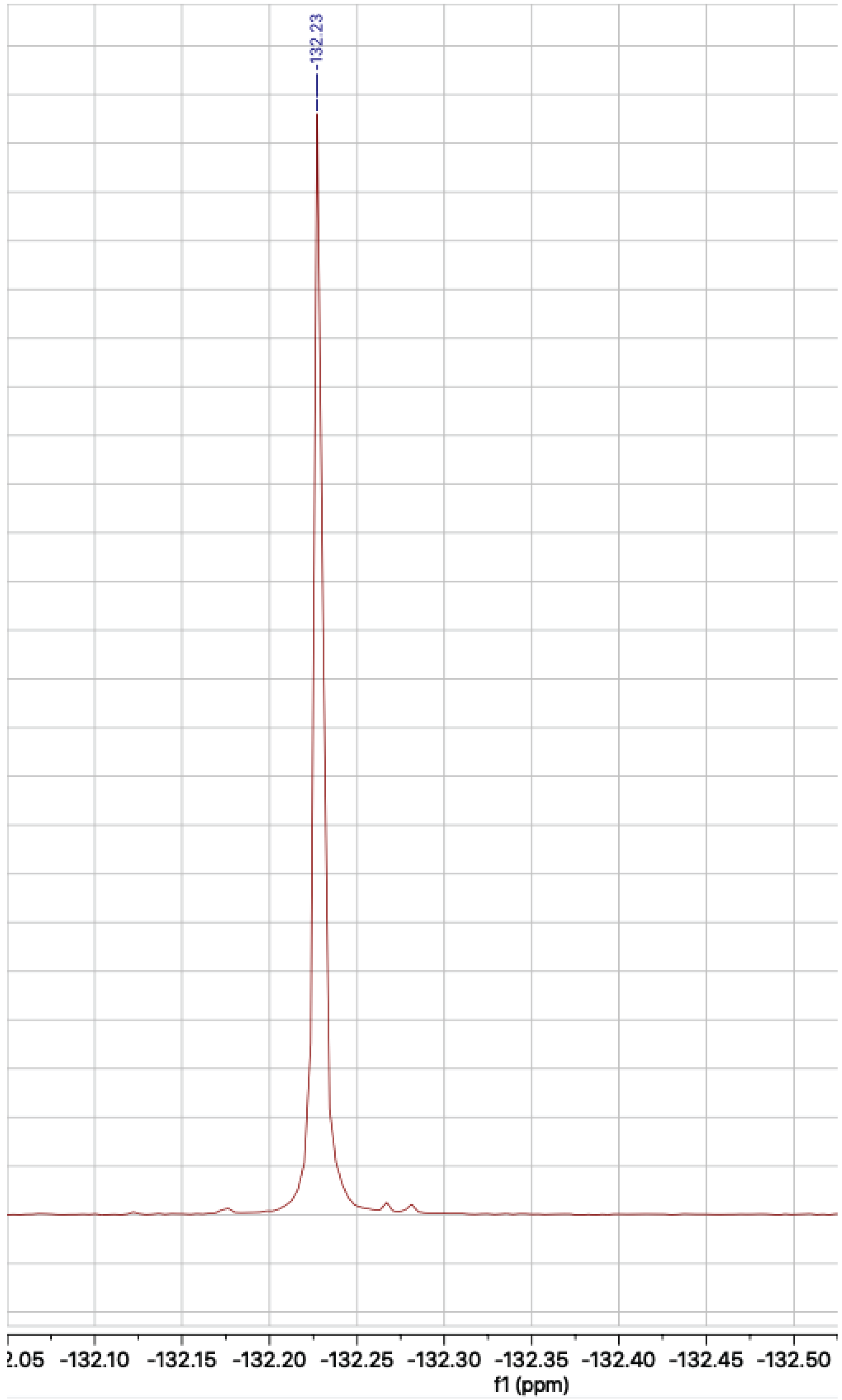

Figure S60: **24**, ^{19}F-NMR spectra.

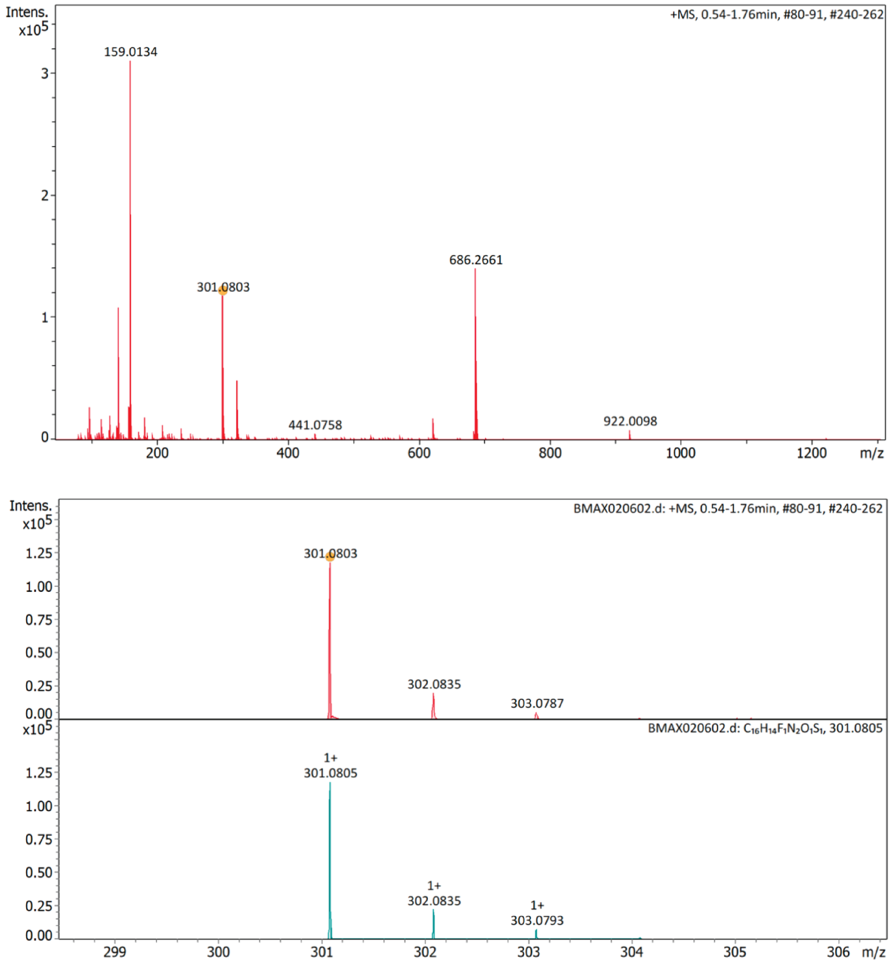

#	Ion Formula	Adduct	m/z	z	Meas. m/z	mSigma	N-Rule	err [mDa]	err [ppm]
1	C16H14FN2OS	M+H	301.0805	1+	301.0803	13.9	ok	0.2	0.7

Figure S61: **24**, HRMS.

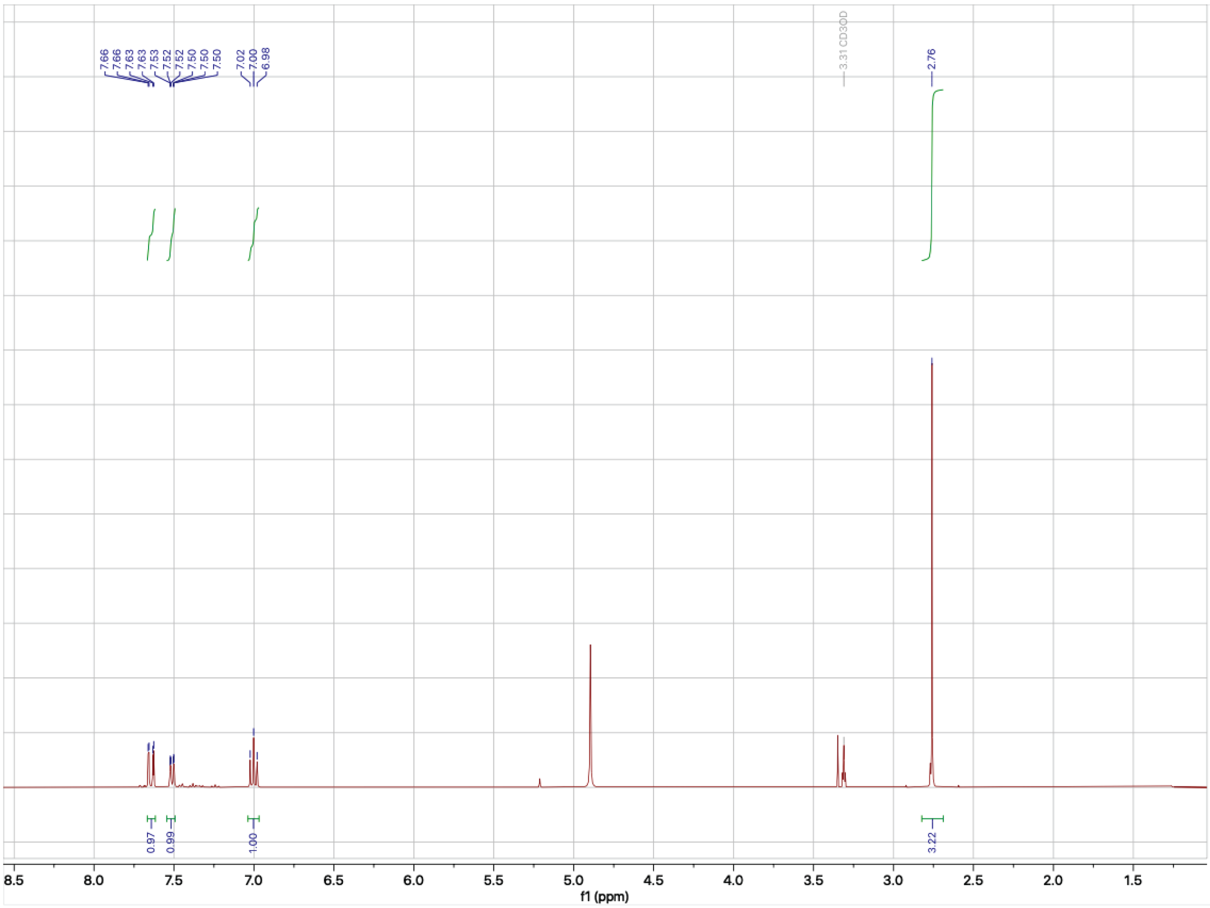

Figure S62: **25**, ^{1}H-NMR spectra.

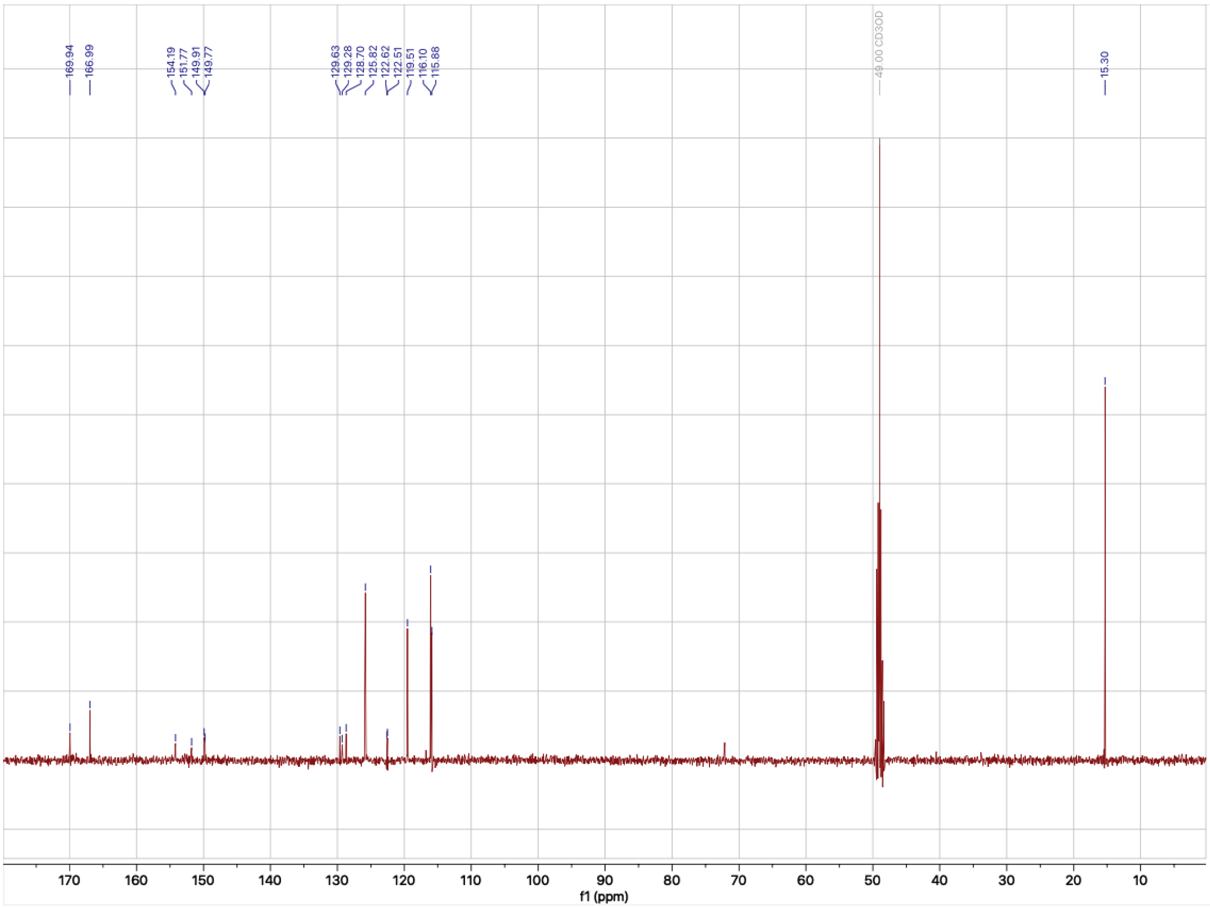

Figure S63: **25**, ^{13}C-NMR spectra.

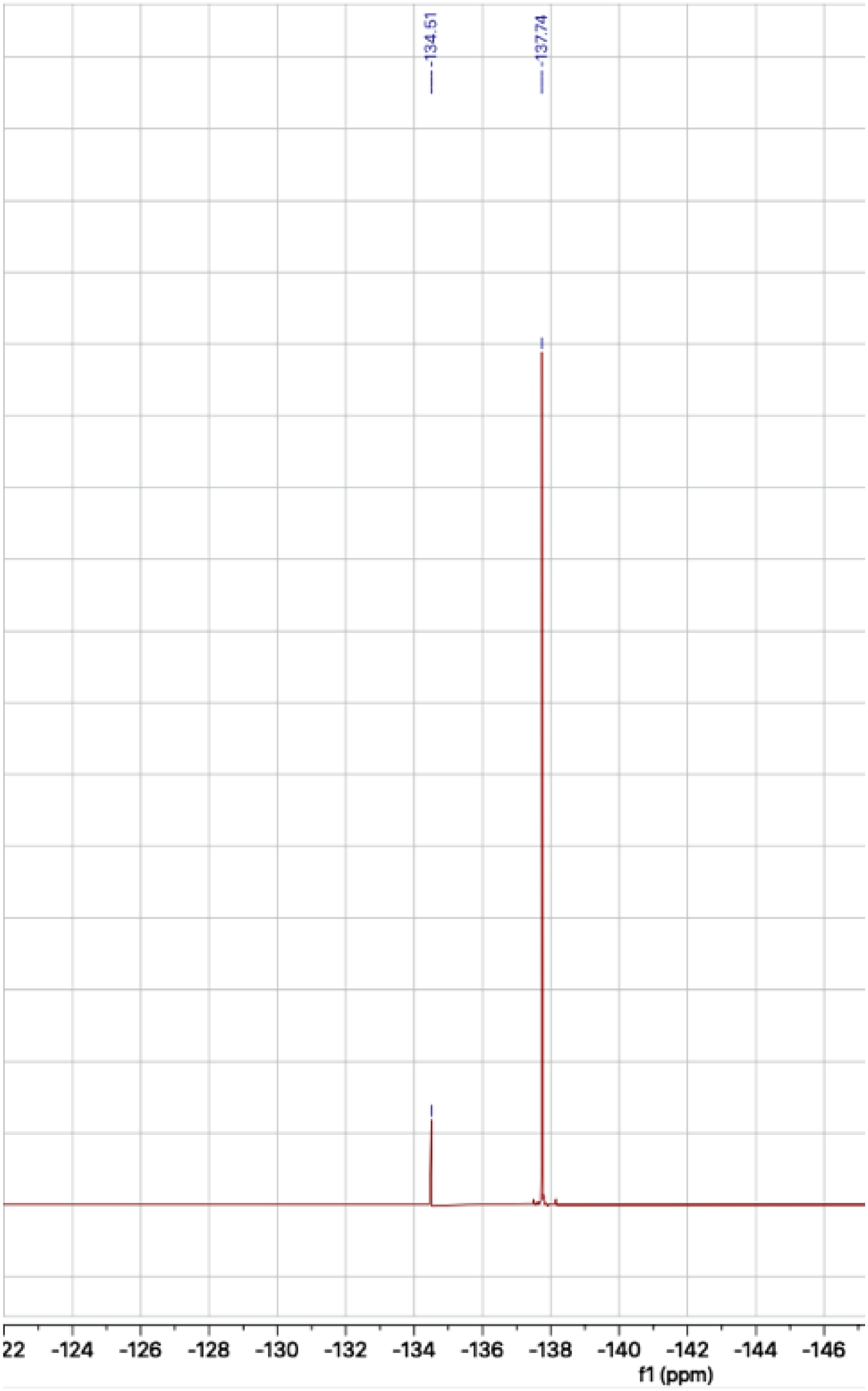

Figure S64: **25**, ^{19}F-NMR spectra.

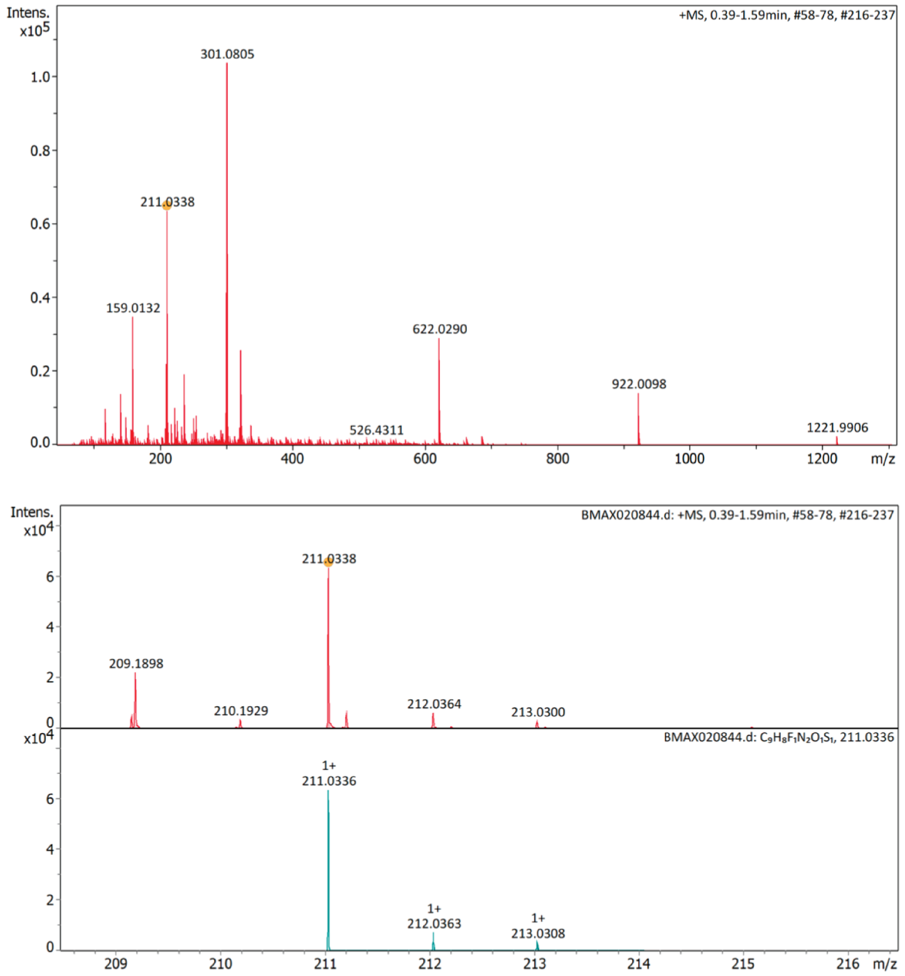

#	Ion Formula	Adduct	m/z	z	Meas. m/z	mSigma	N-Rule	err [mDa]	err [ppm]
1	C9H8FN2OS	M+H	211.0336	1+	211.0338	9.9	ok	-0.2	-0.9

Figure S65: **25**, HRMS.

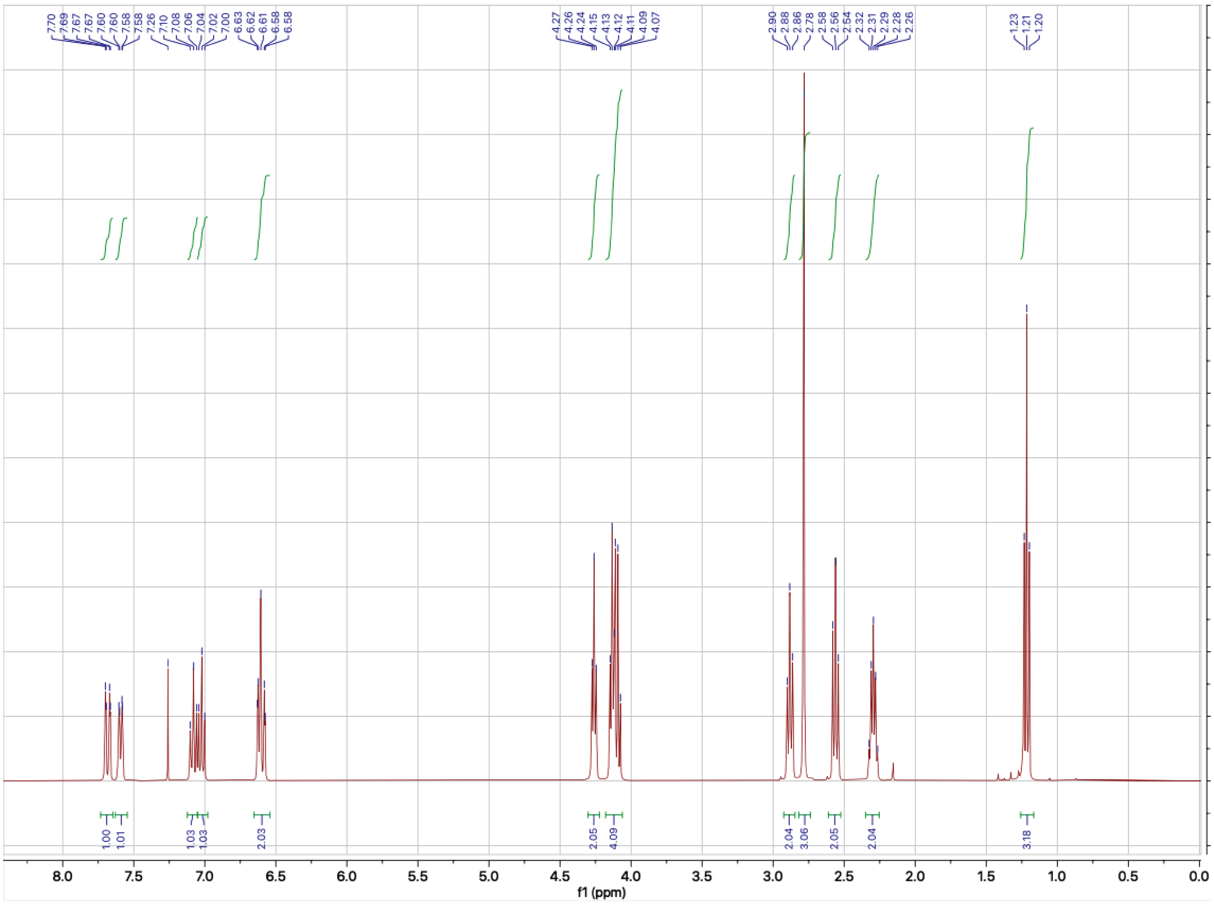

Figure S66: **26**, ^{1}H-NMR spectra.

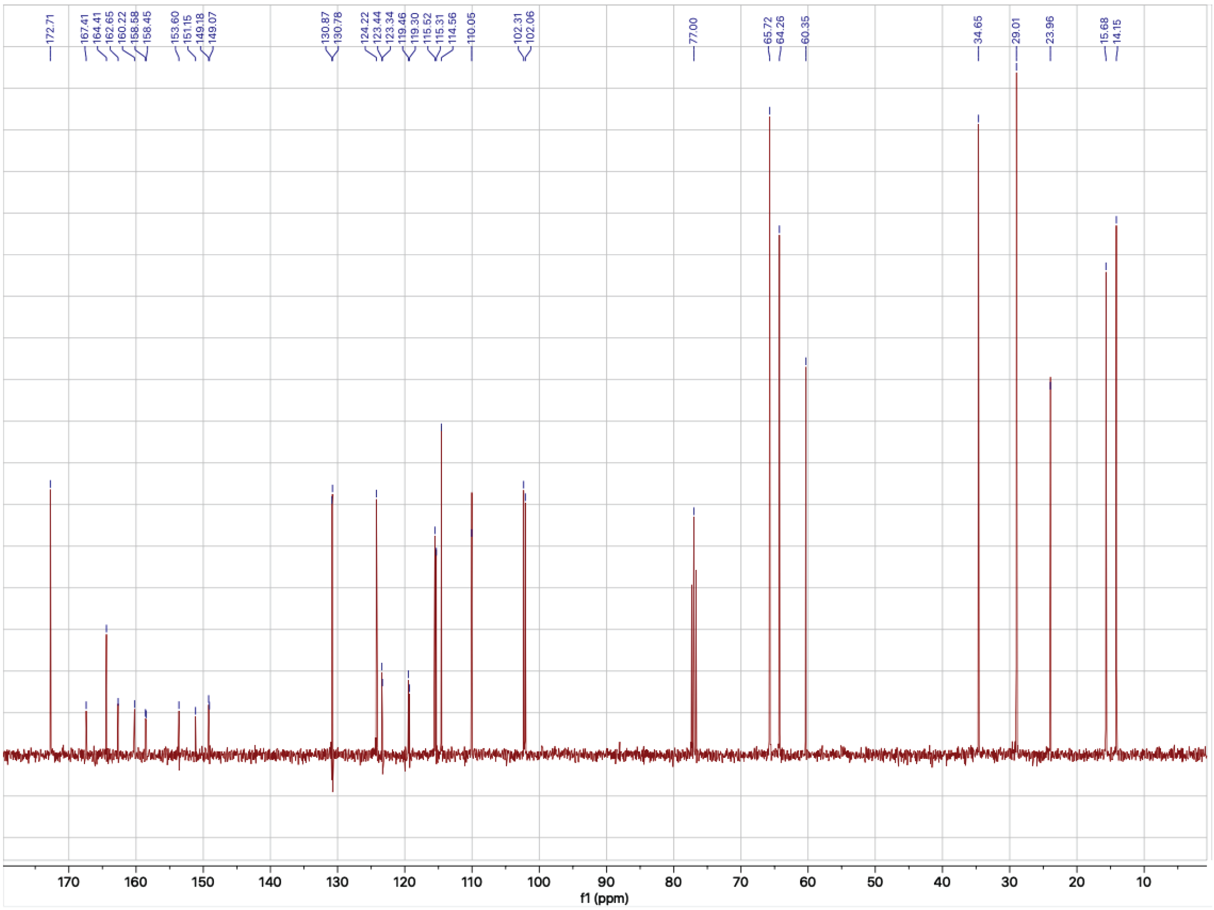

Figure S67: **26**, ^{13}C-NMR spectra.

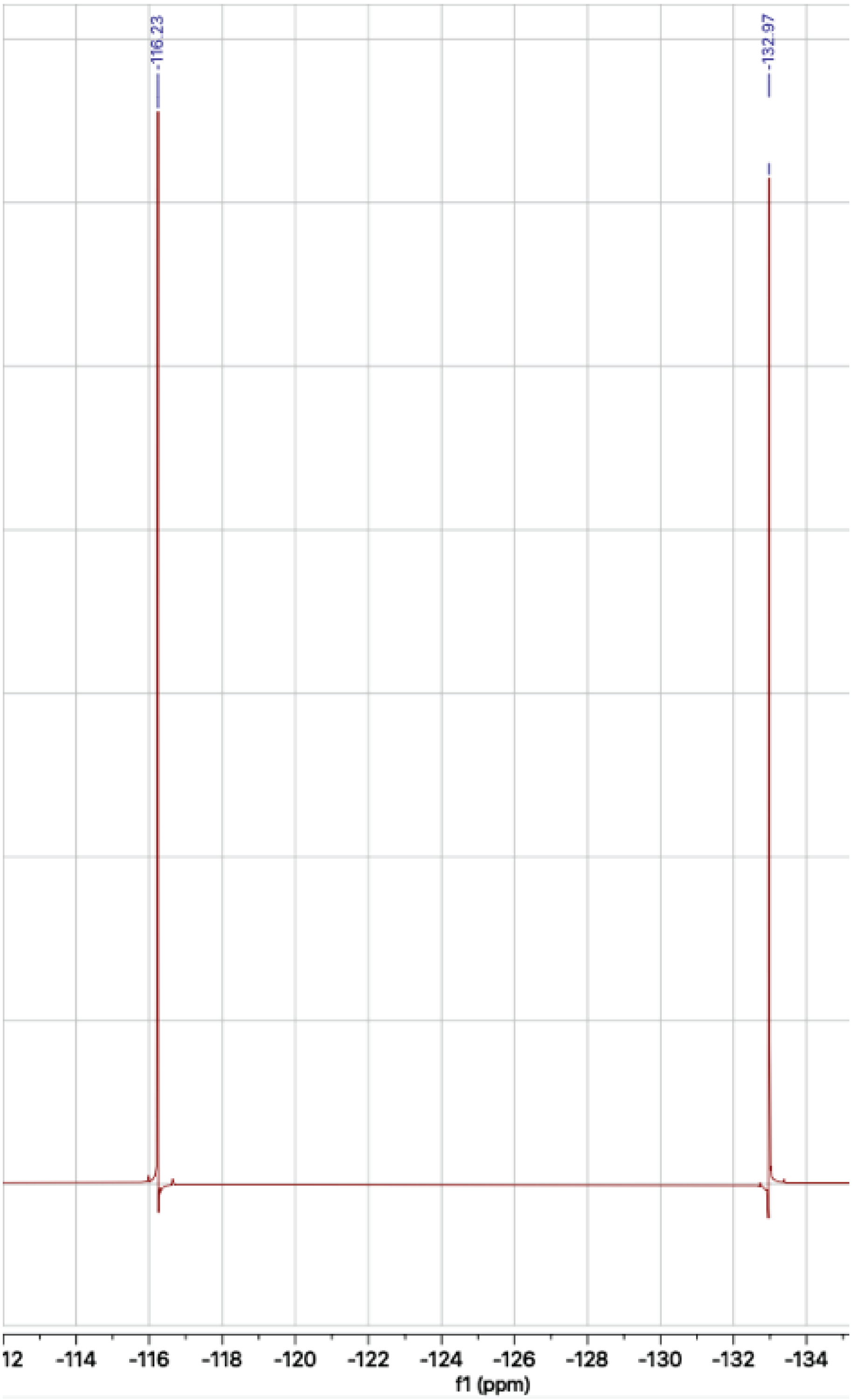

Figure S68: **26**, ^{19}F-NMR spectra.

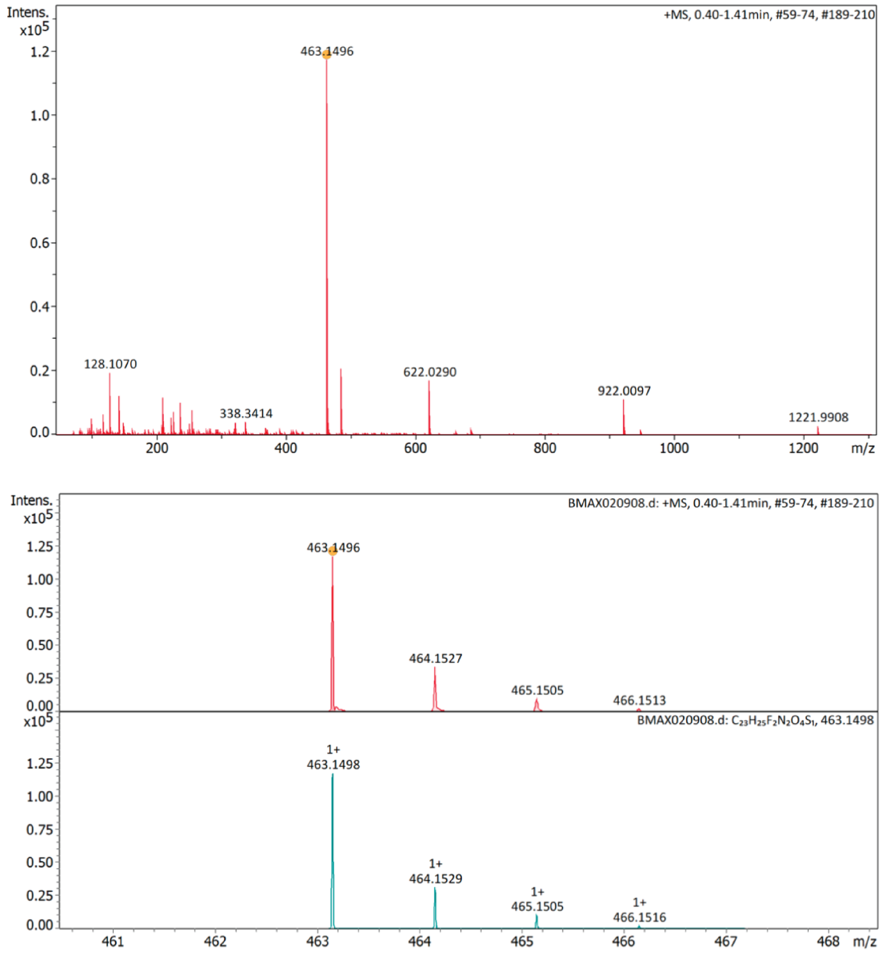

#	Ion Formula	Adduct	m/z	z	Meas. m/z	mSigma	N-Rule	err [mDa]	err [ppm]
1	C23H25F2N2O4S	M+H	463.1498	1+	463.1496	9.3	ok	0.1	0.3

Figure S69: **26**, HRMS.

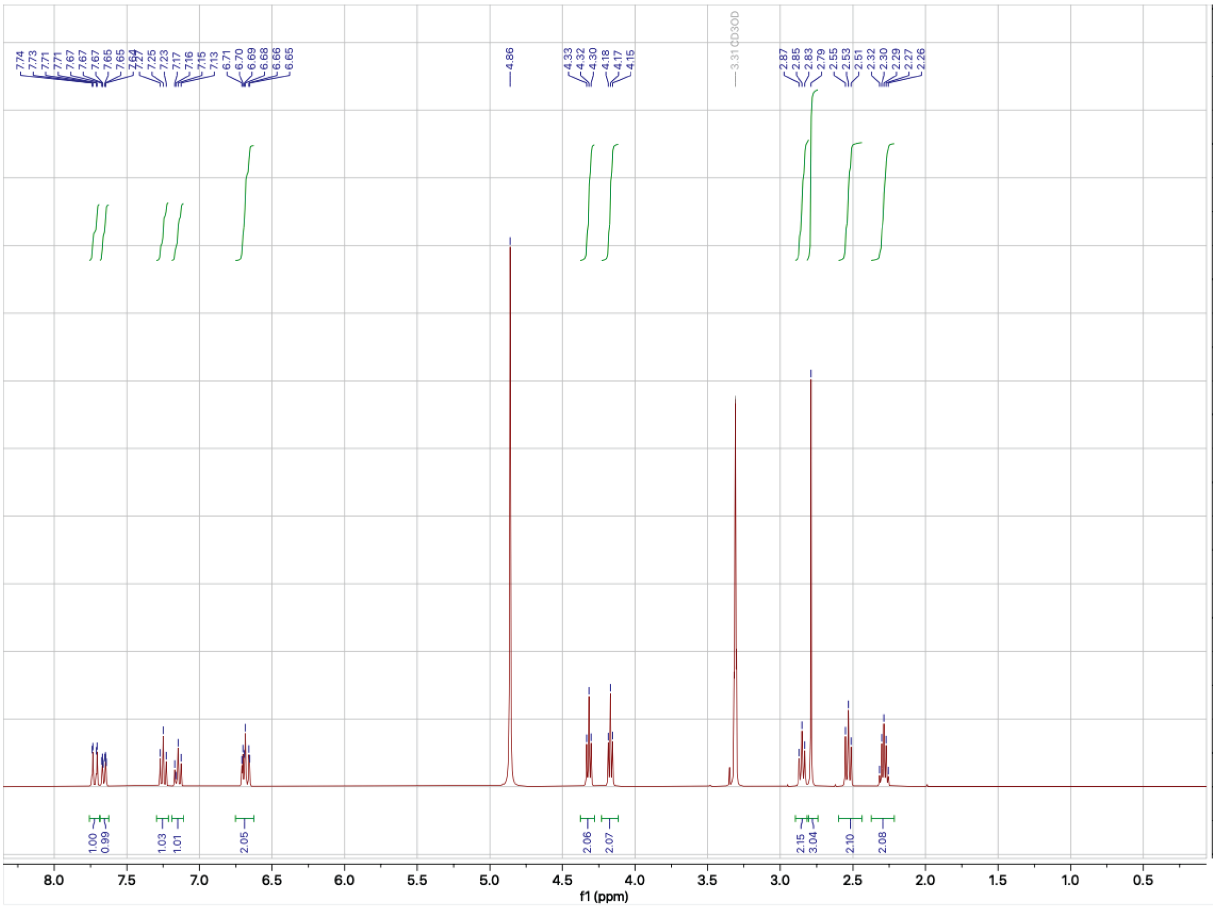

Figure S70: **1**, ^{1}H-NMR spectra.

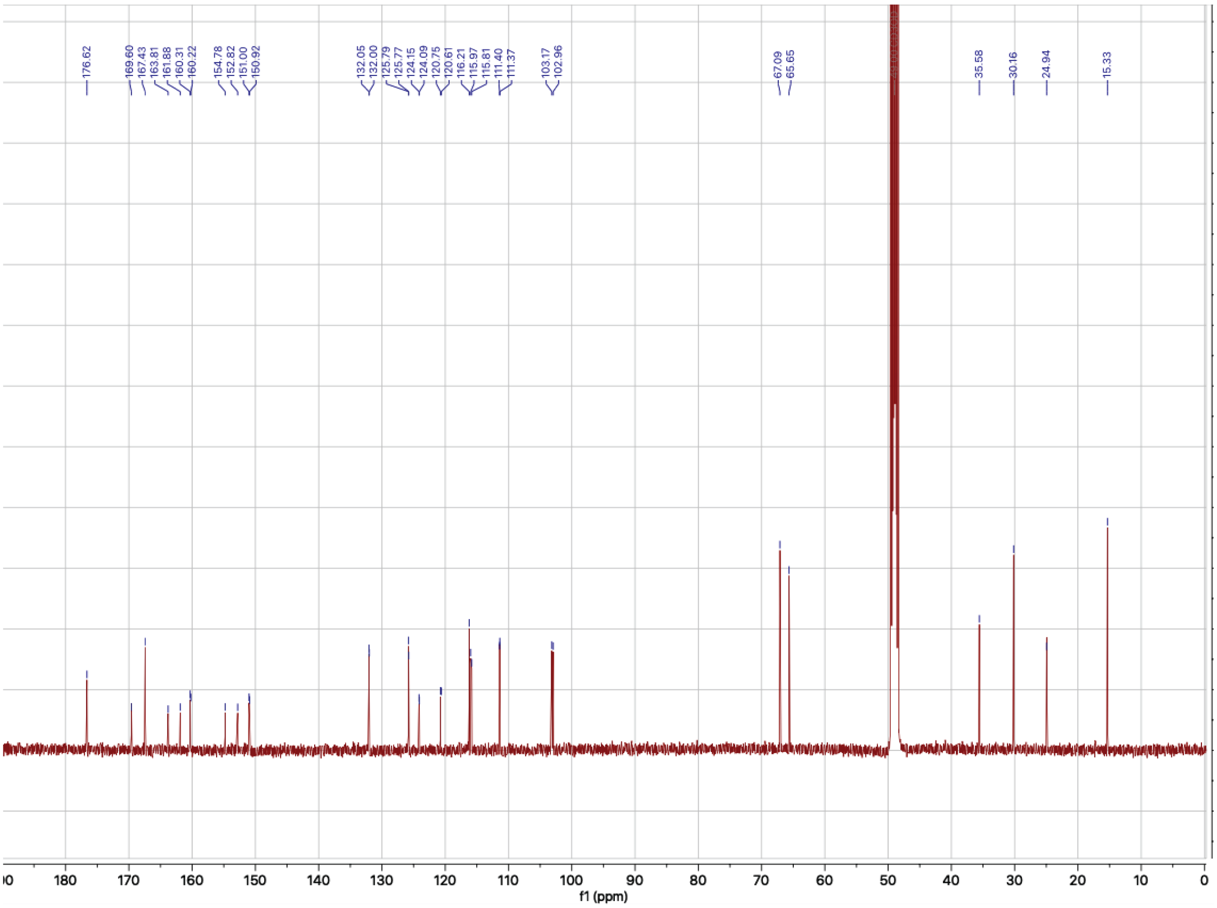

Figure S71: **1**, ^{13}C-NMR spectra.

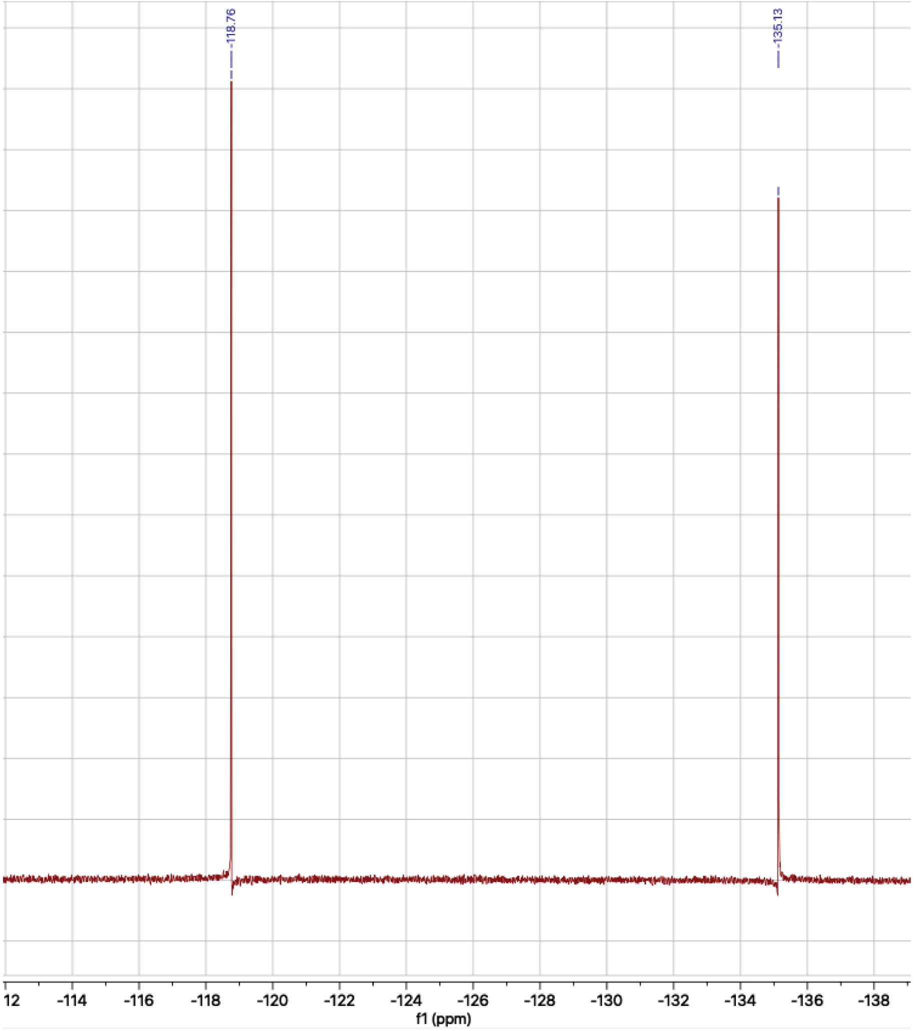

Figure S72: **1**, ^{19}F-NMR spectra.

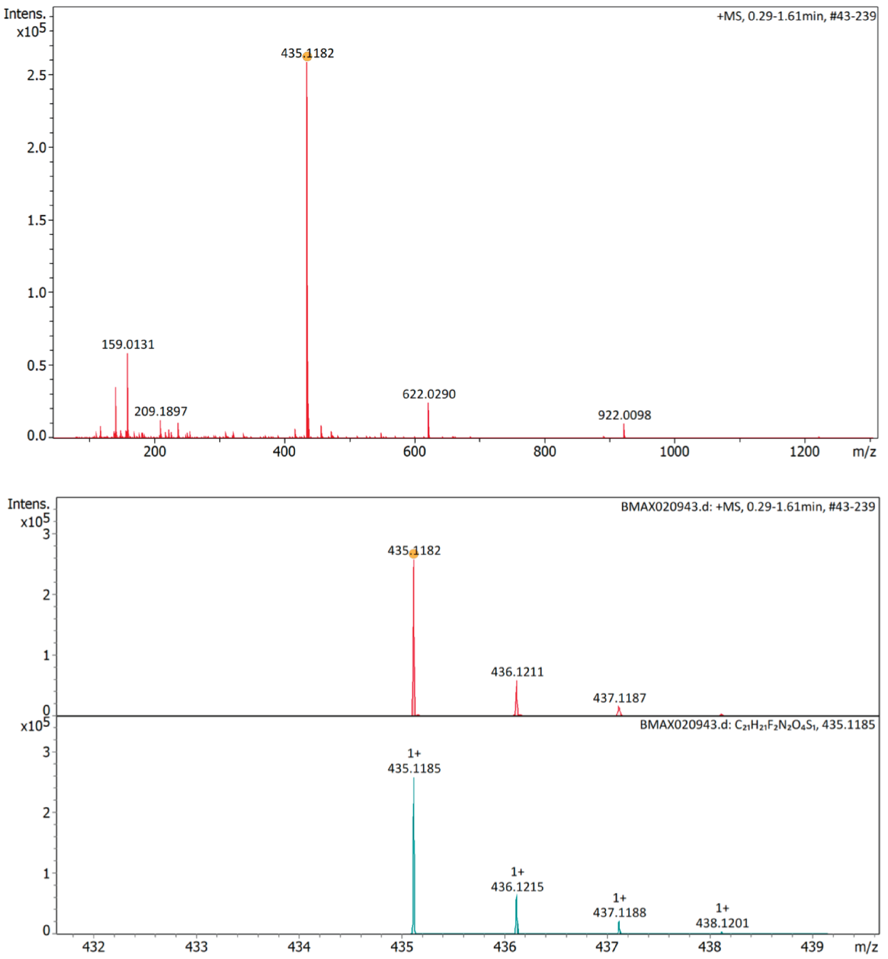

#	Ion Formula	Adduct	m/z	z	Meas. m/z	mSigma	N-Rule	err [mDa]	err [ppm]
1	C21H21F2N2O4S	M+H	435.1185	1+	435.1182	15.2	ok	0.2	0.5

Figure S73: **1**, HRMS.

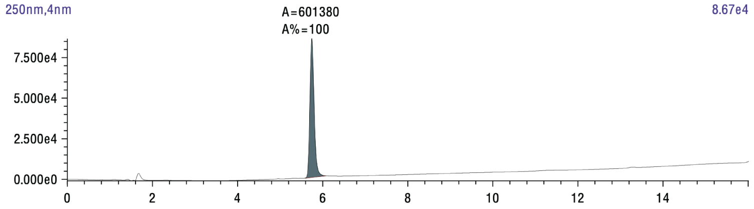

Pk#	RT	Area	Area %	Height	Height %
8	5.744	601'380	100.000	85'460	100.000
**Totals:**		**601'380**	**100.000**	**85'460**	**100.000**

Figure S74: **1**, HPLC.

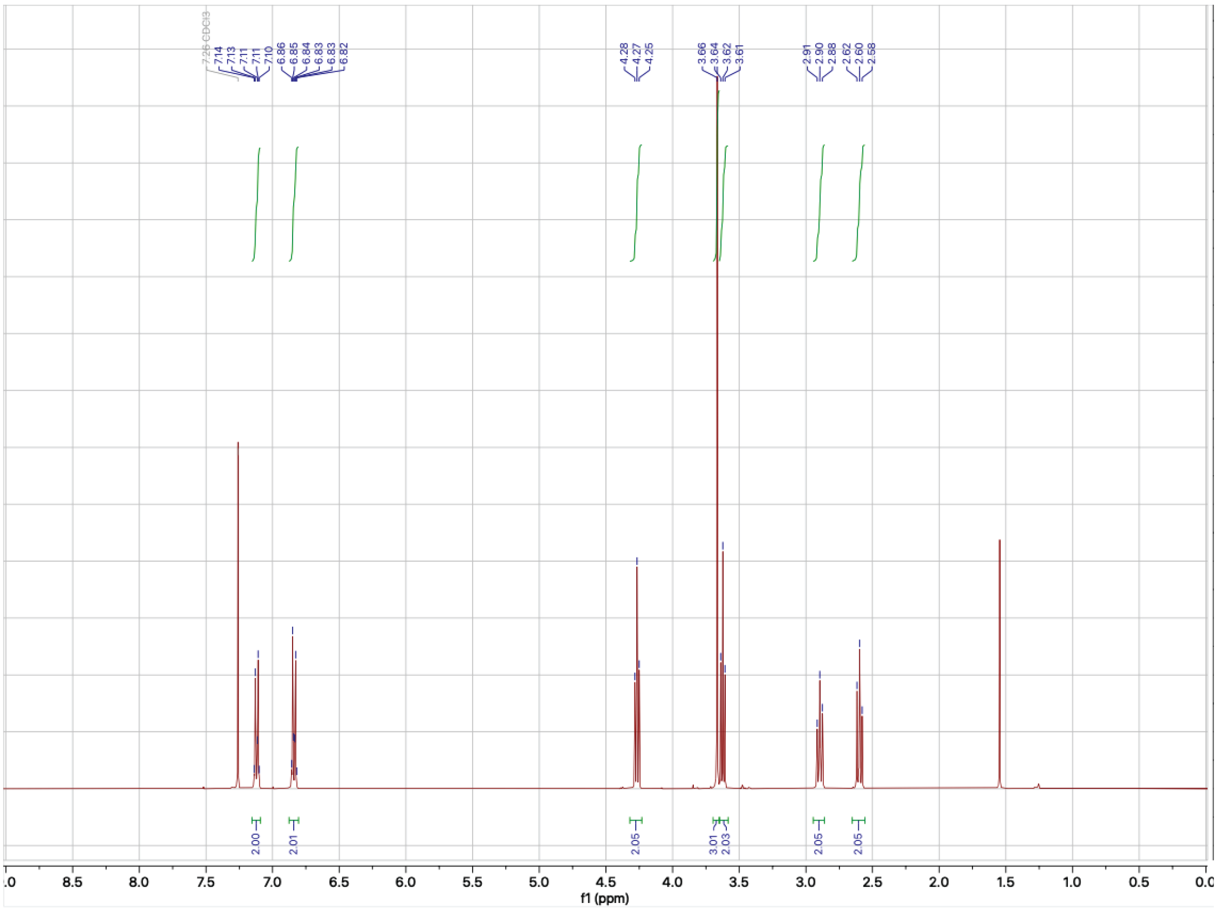

Figure S75: **29**, ^{1}H-NMR spectra.

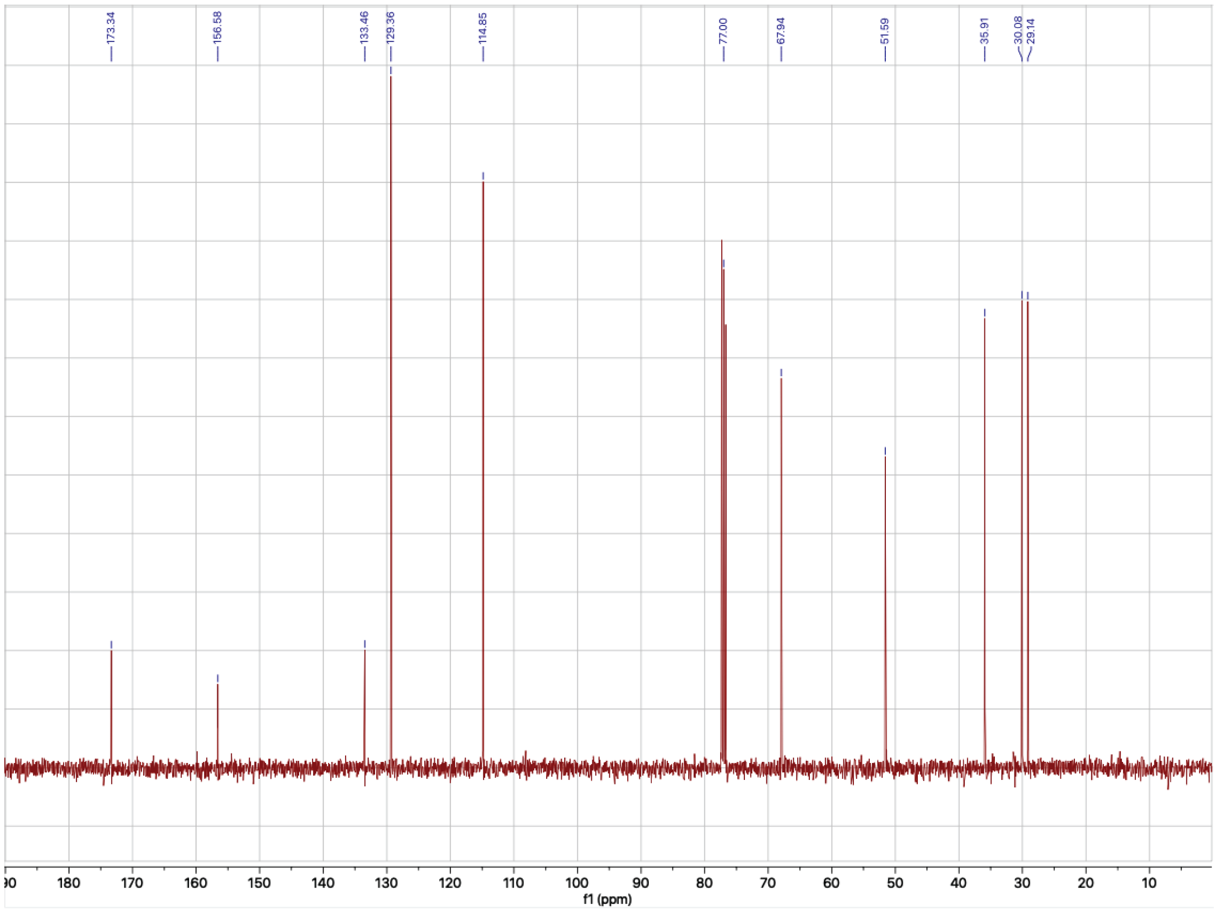

Figure S76: **29**, ^{13}C-NMR spectra.

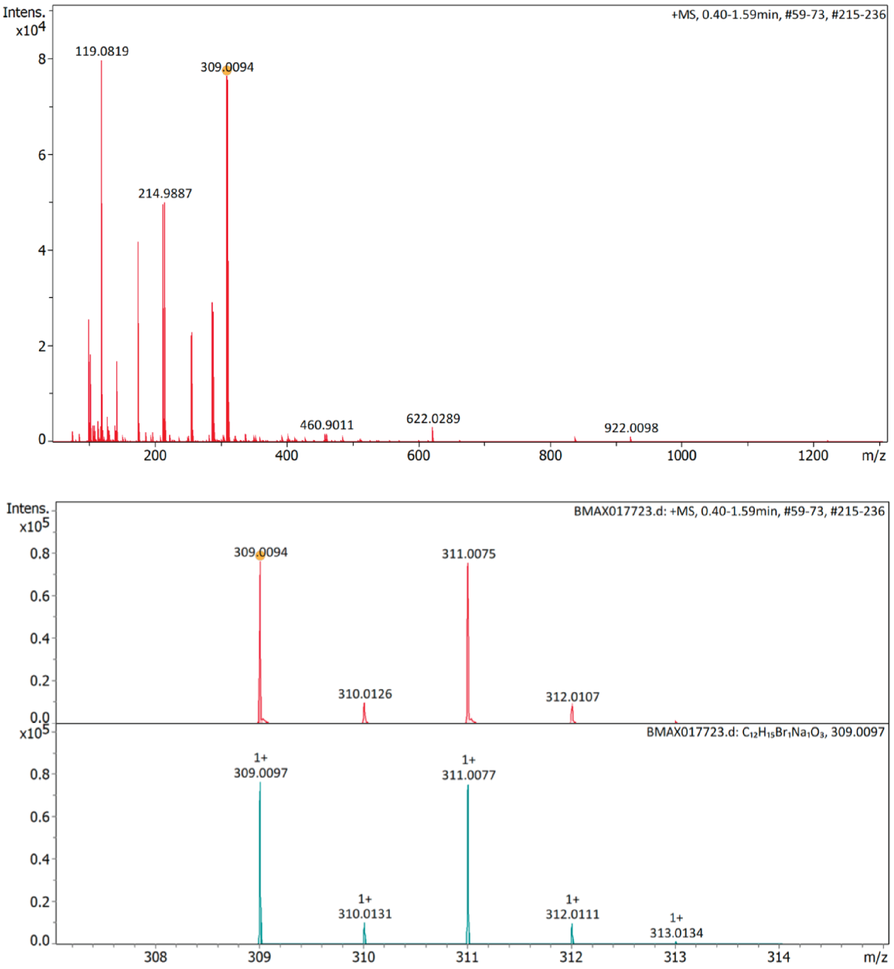

#	Ion Formula	Adduct	m/z	z	Meas. m/z	mSigma	N-Rule	err [mDa]	err [ppm]
1	C12H15BrNaO3	M+Na	309.0097	1+	309.0094	9.2	ok	0.3	0.8

Figure S77: **29**, HRMS.

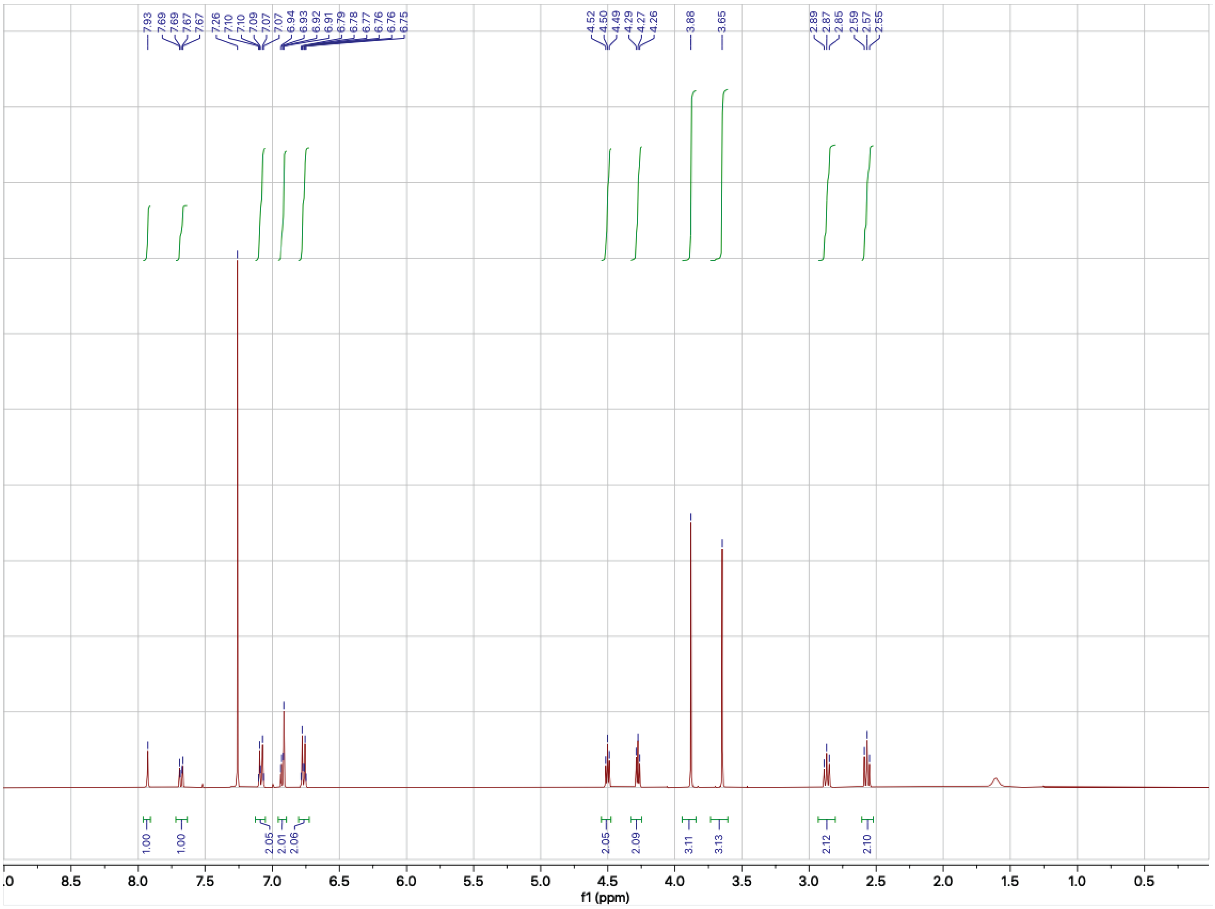

Figure S78: **31**, ^{1}H-NMR spectra.

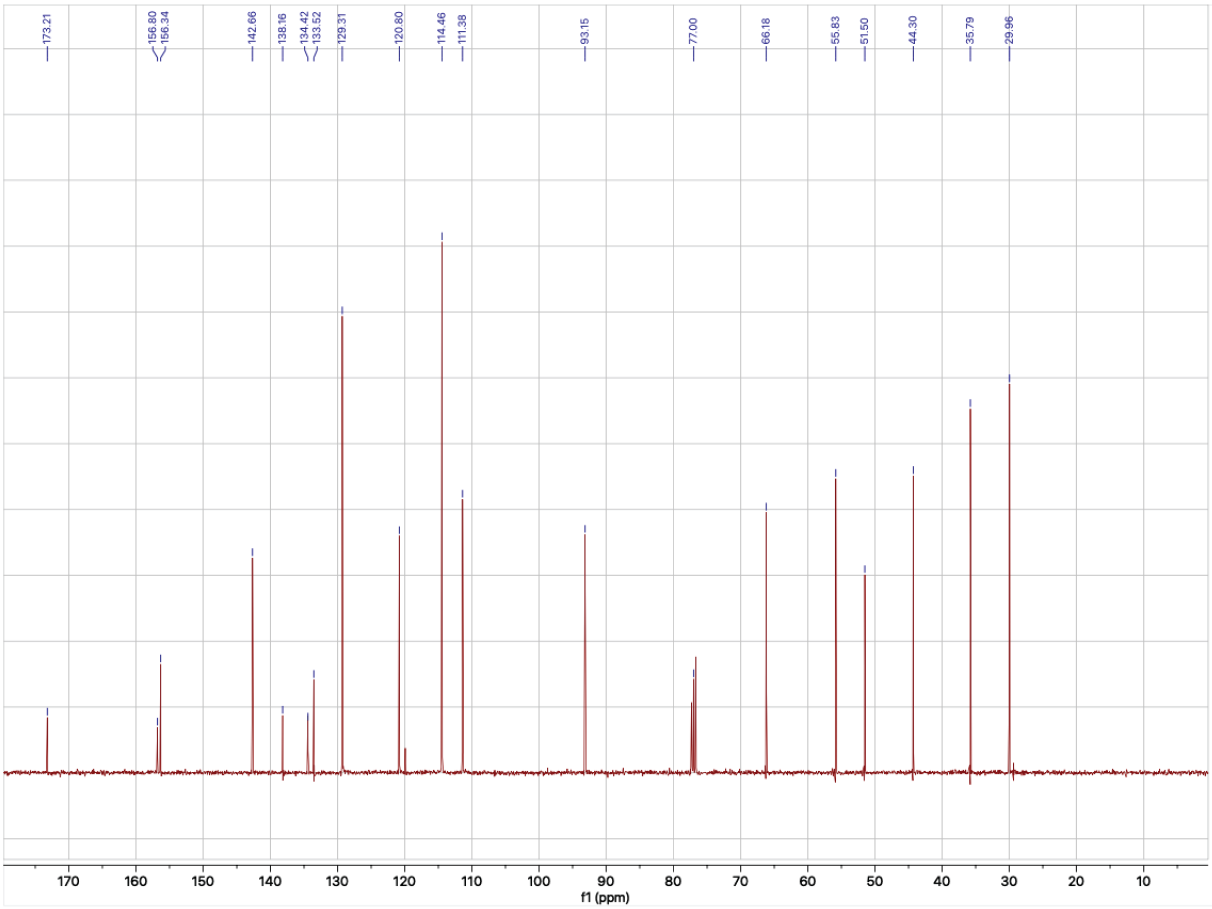

Figure S79: **31**, ^{13}C-NMR spectra.

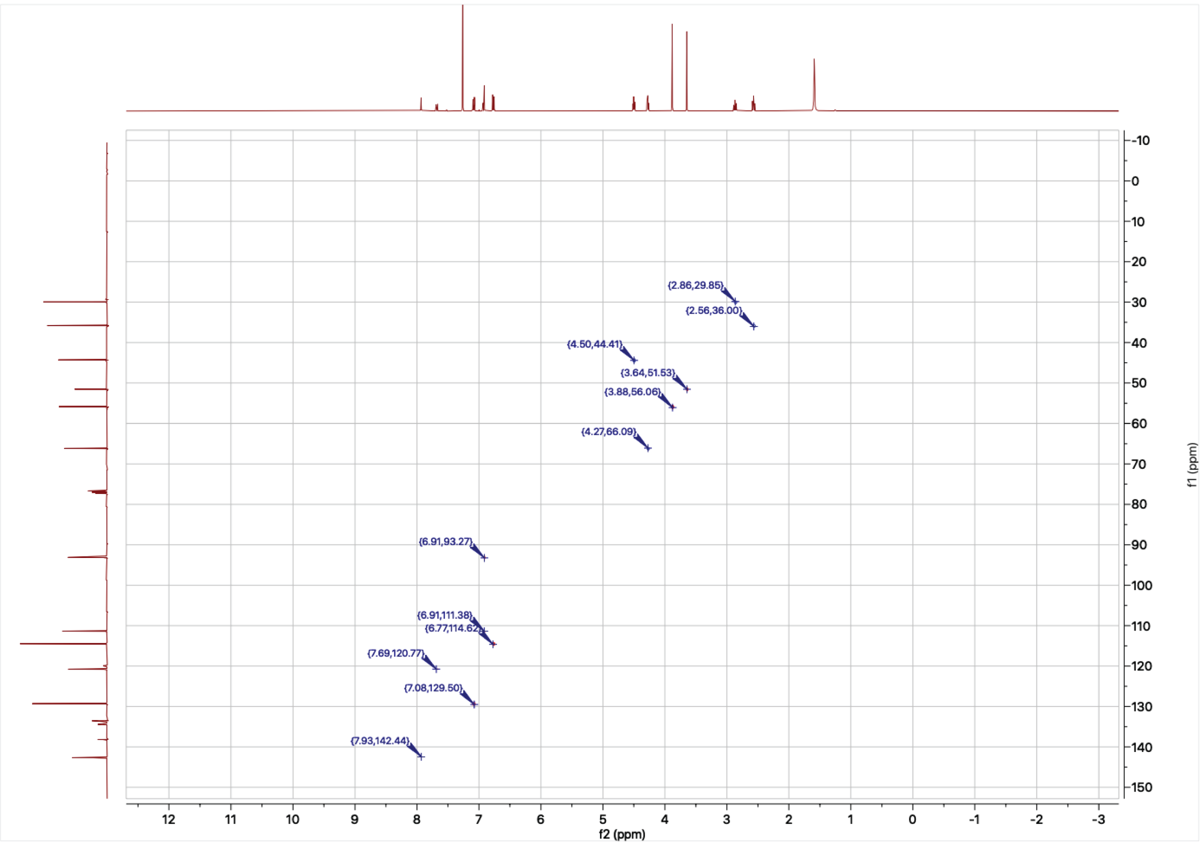

Figure S80: **31**, HSQC-NMR spectra.

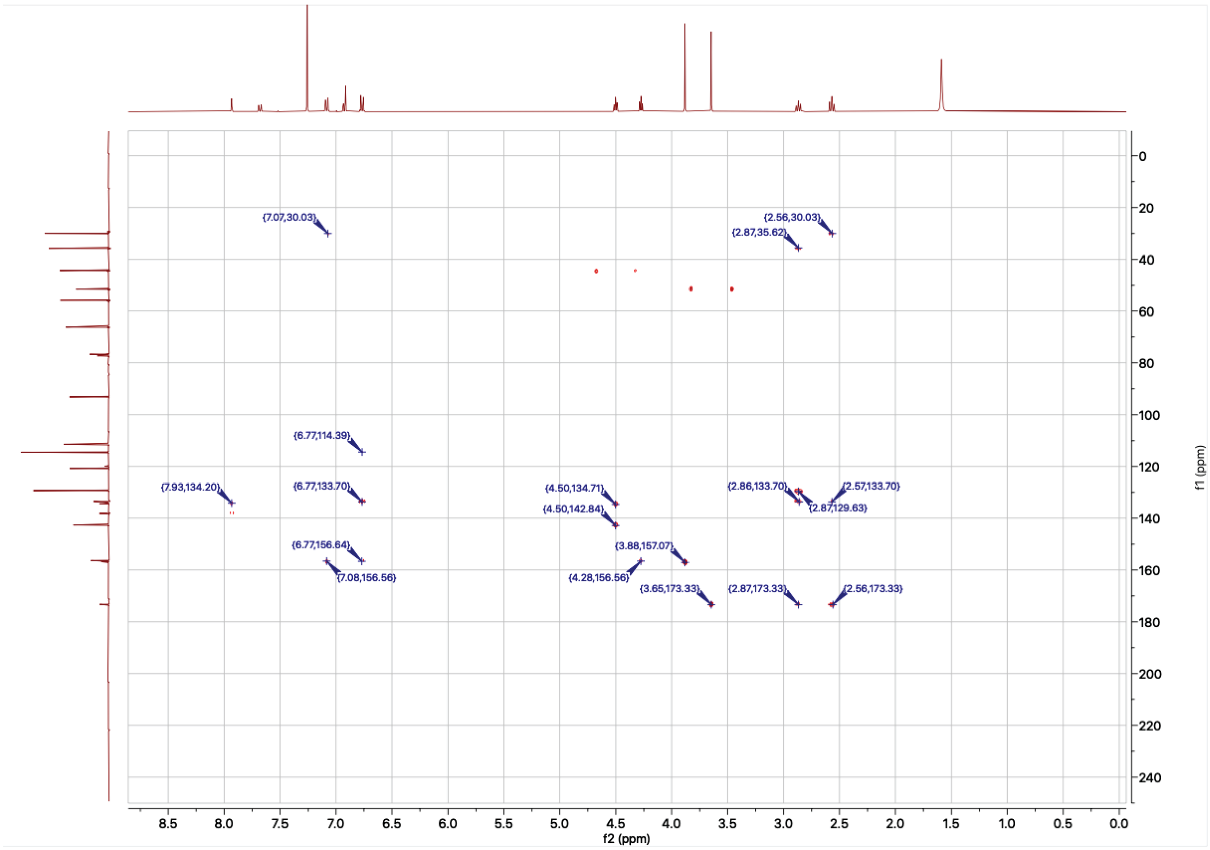

Figure S81: **31**, HMBC-NMR spectra.

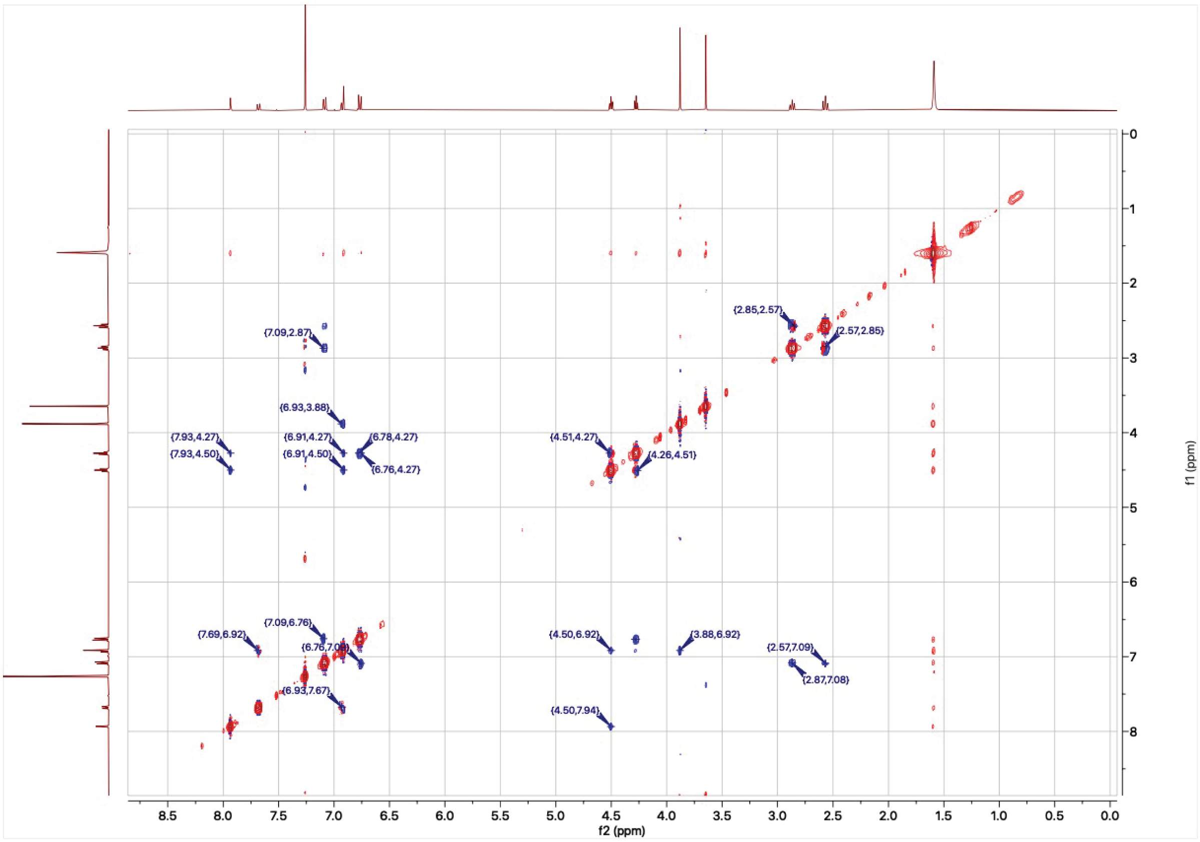

Figure S82: **31**, NOESY-NMR spectra.

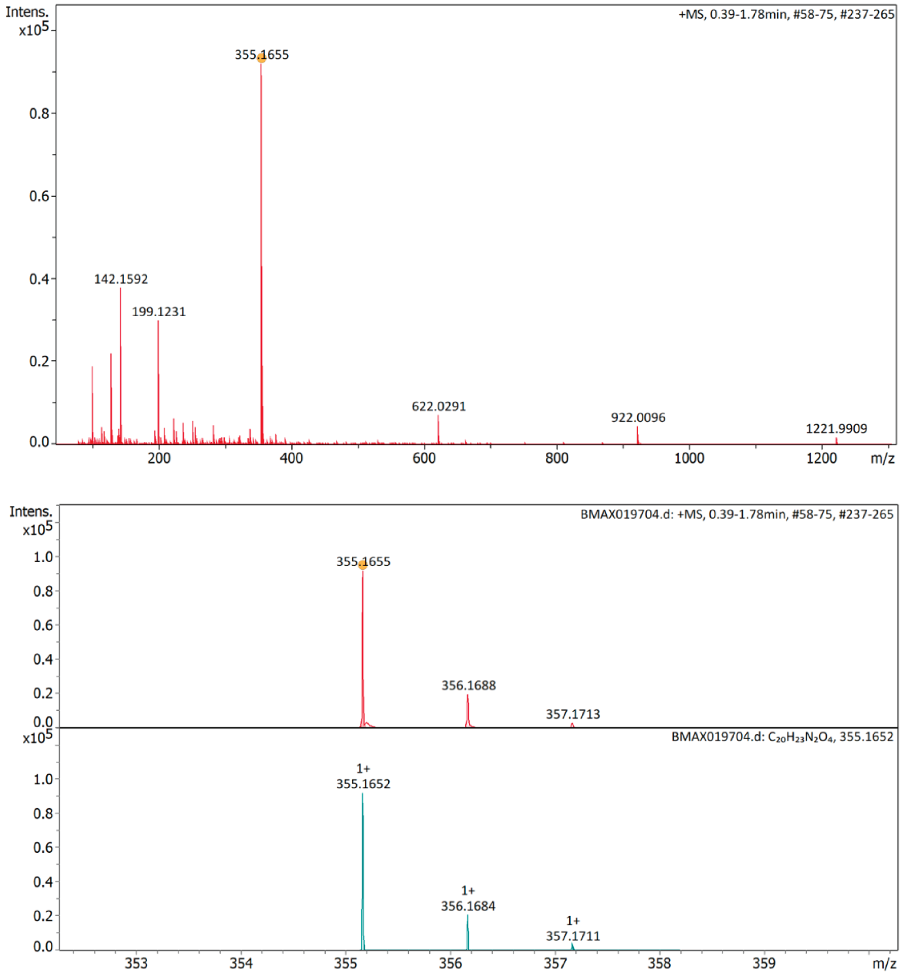

#	Ion Formula	Adduct	m/z	z	Meas. m/z	mSigma	N-Rule	err [mDa]	err [ppm]
1	C20H23N2O4	M+H	355.1652	1+	355.1655	7.4	ok	-0.2	-0.7

Figure S83: **31**, HRMS.

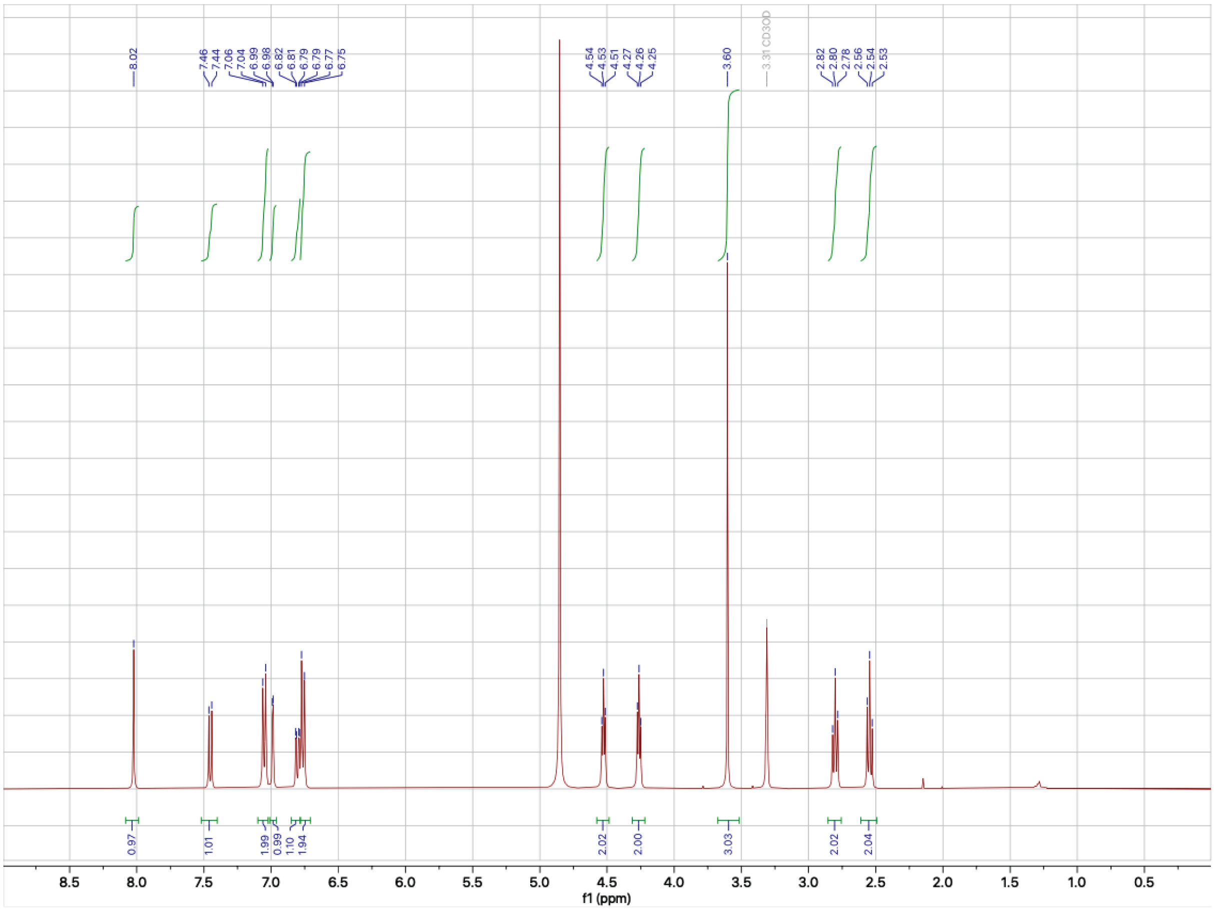

Figure S84: **33**, ^{1}H-NMR spectra.

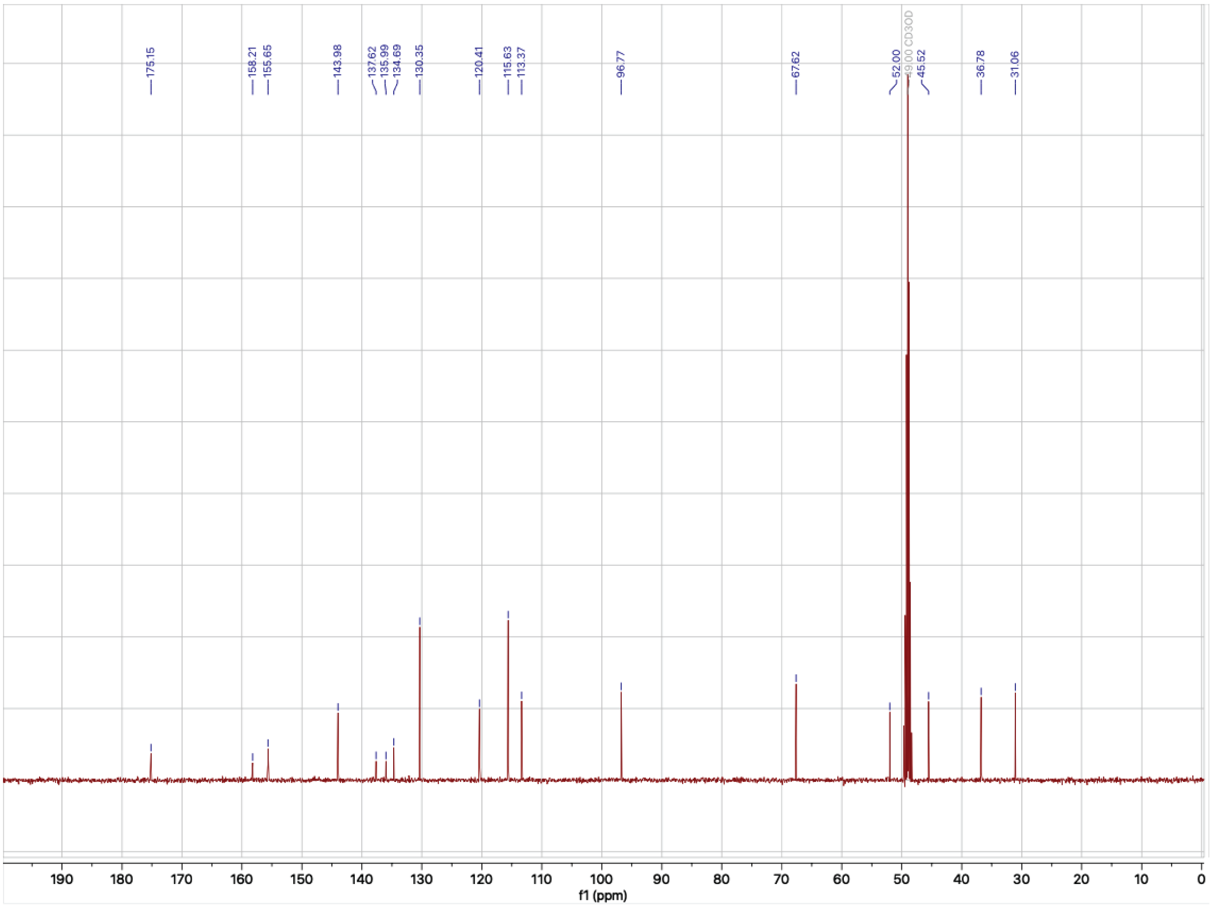

Figure S85: **33**, ^{13}C-NMR spectra.

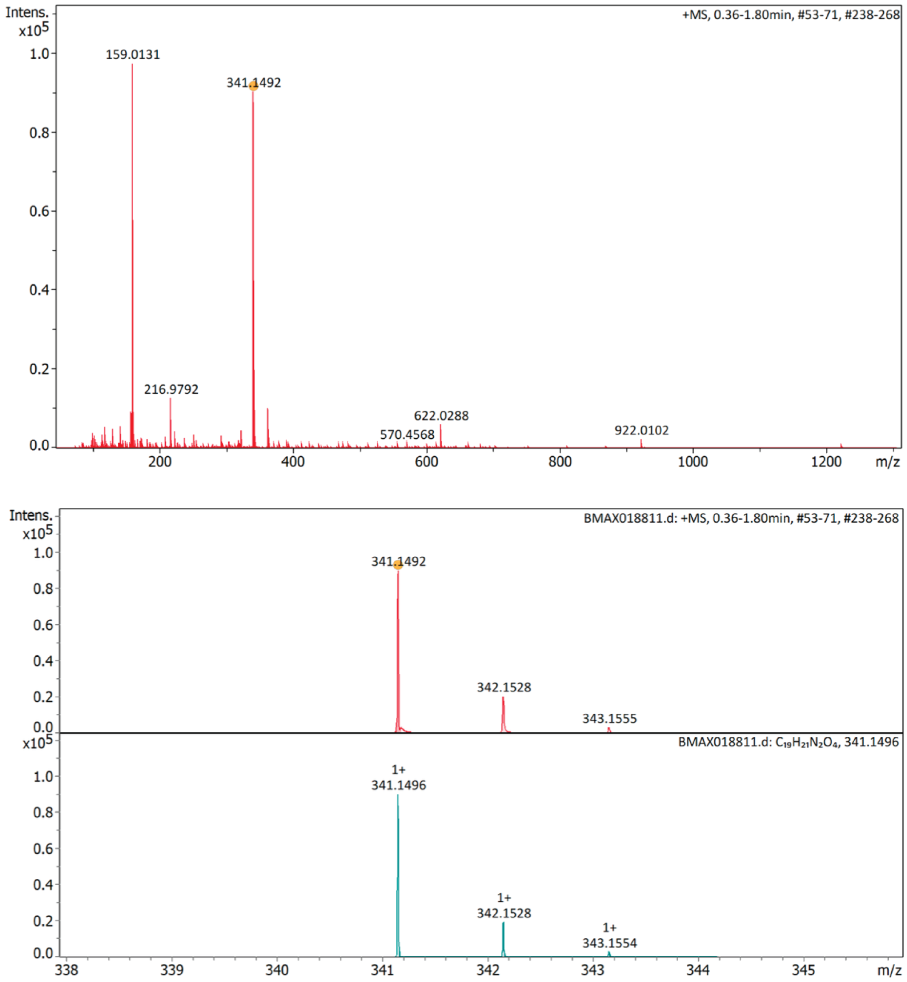

#	Ion Formula	Adduct	m/z	z	Meas. m/z	mSigma	N-Rule	err [mDa]	err [ppm]
1	C19H21N2O4	M+H	341.1496	1+	341.1492	6.6	ok	0.4	1.2

Figure S86: **33**, HRMS.

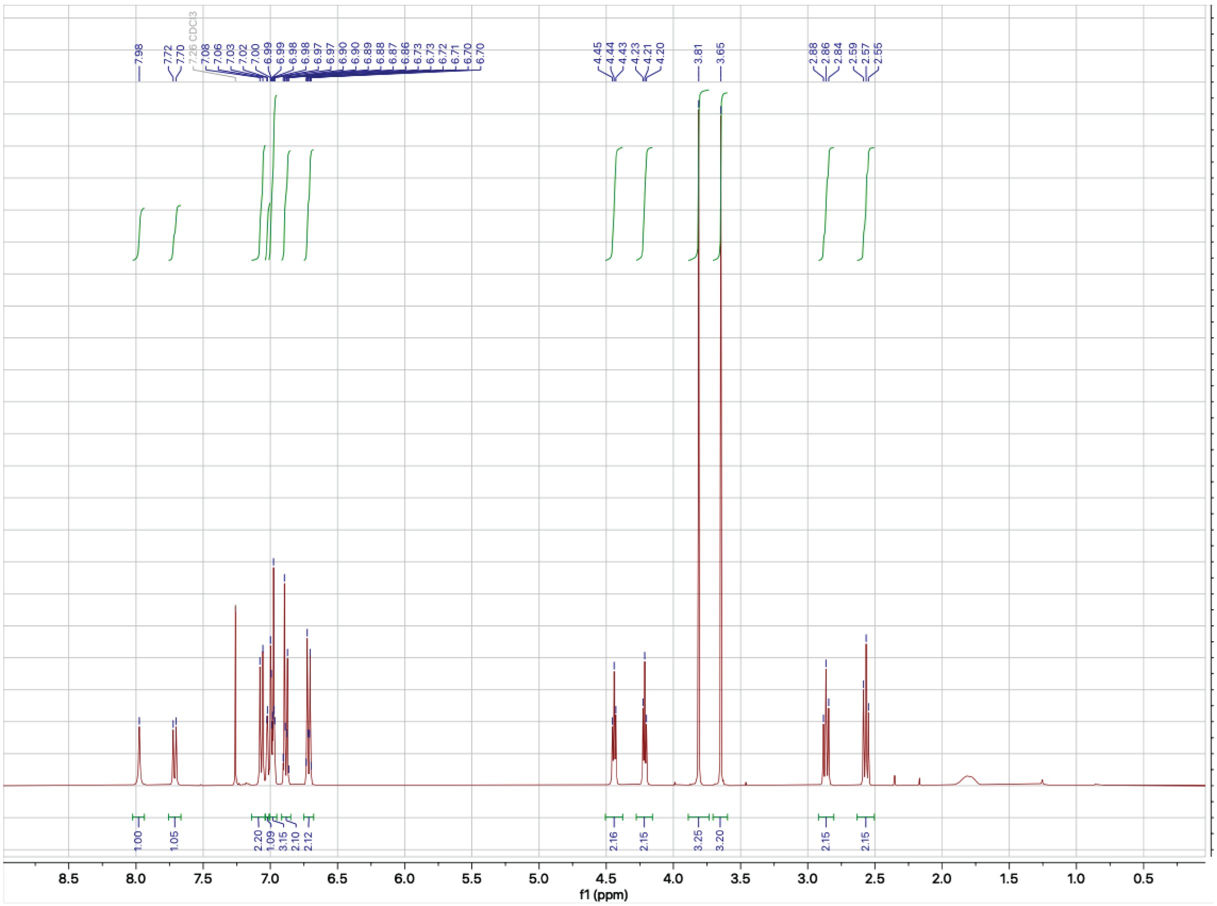

Figure S87: **35**, ^{1}H-NMR spectra.

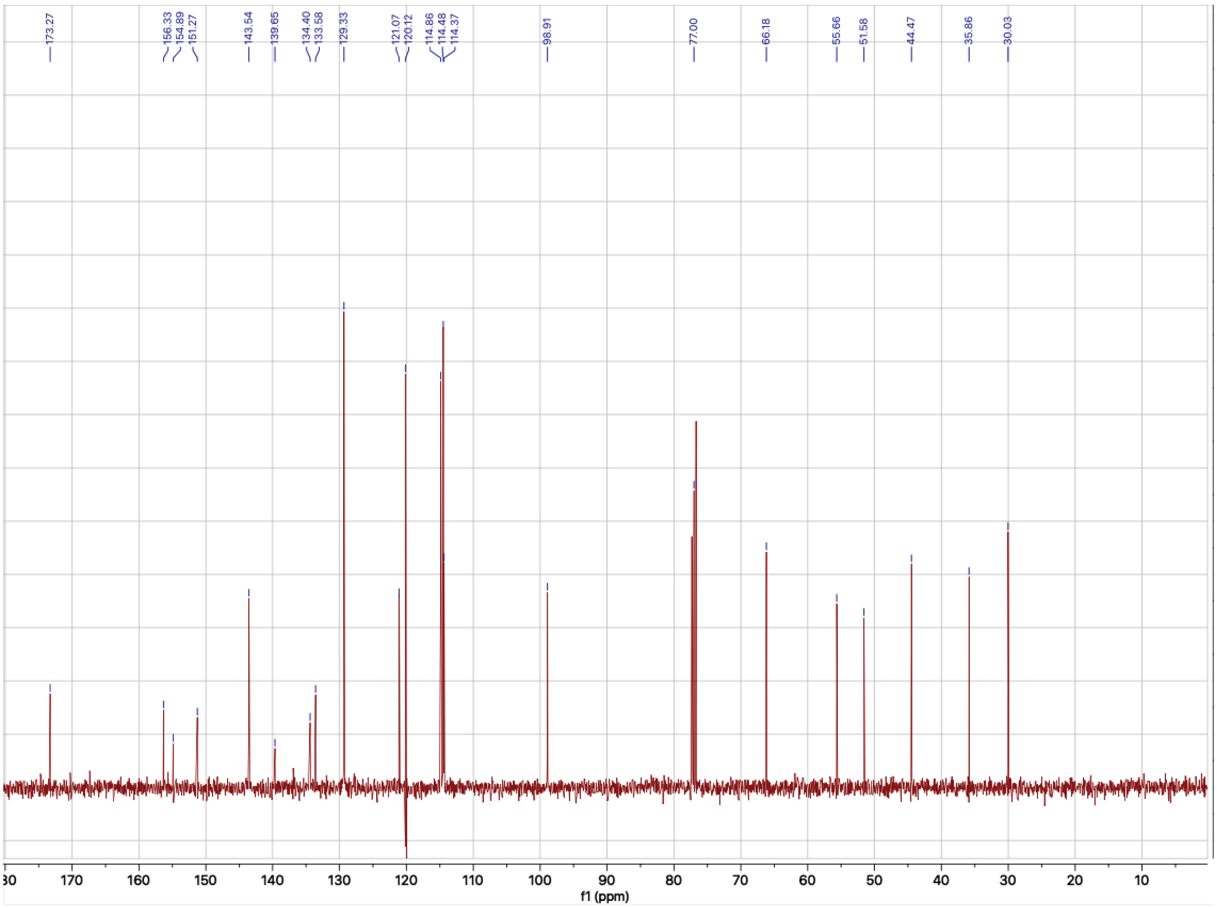

Figure S88: **35**, ^{13}C-NMR spectra.

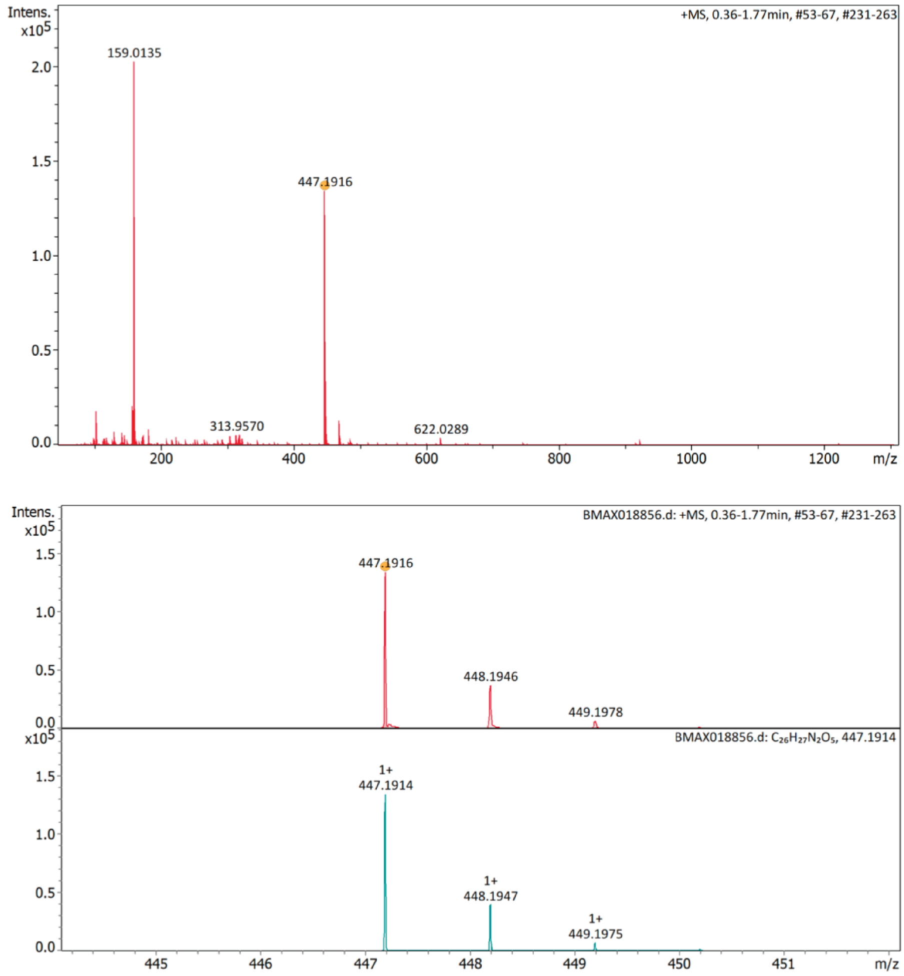

#	Ion Formula	Adduct	m/z	z	Meas. m/z	mSigma	N-Rule	err [mDa]	err [ppm]
1	C26H27N2O5	M+H	447.1914	1+	447.1916	8.9	ok	-0.2	-0.4

Figure S89: **35**, HRMS.

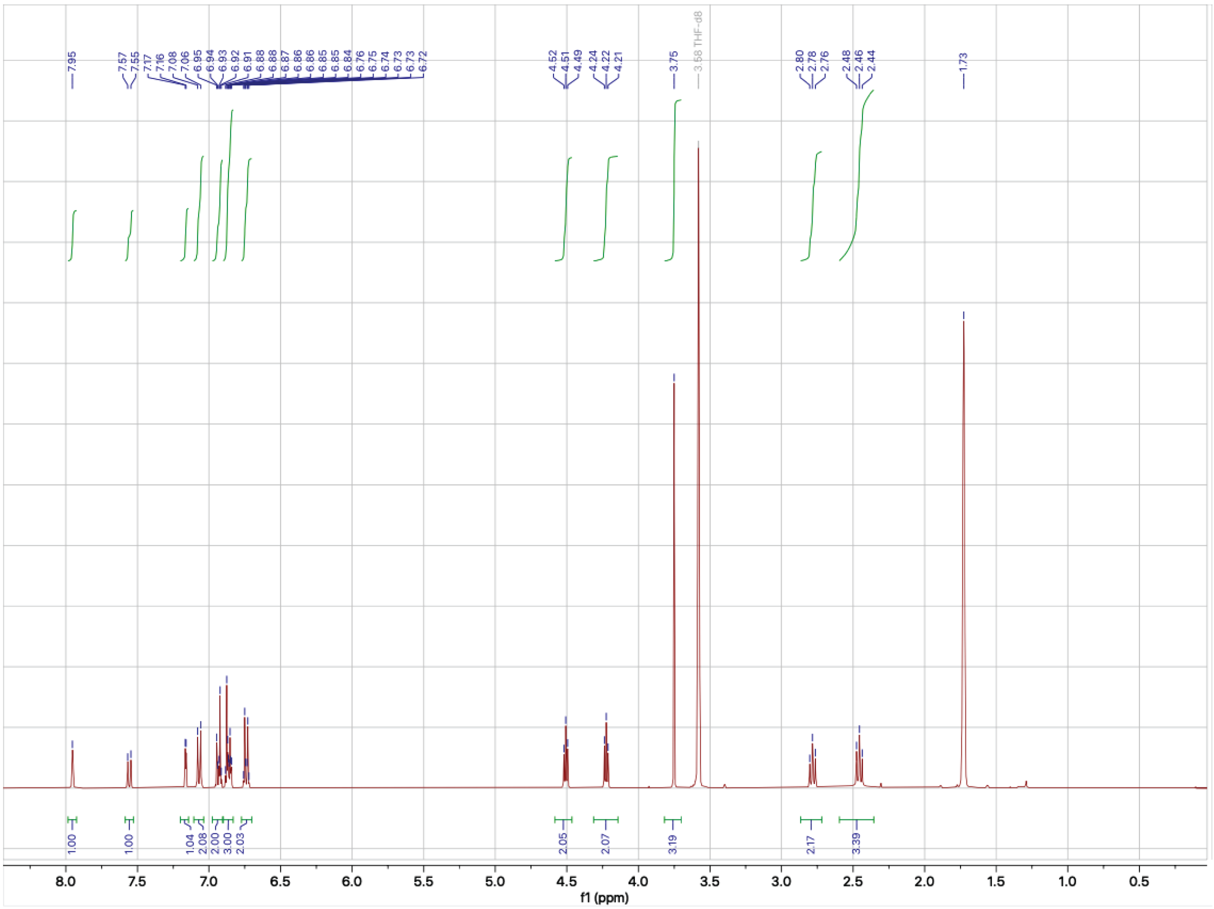

Figure S90: **2**, ^{1}H-NMR spectra.

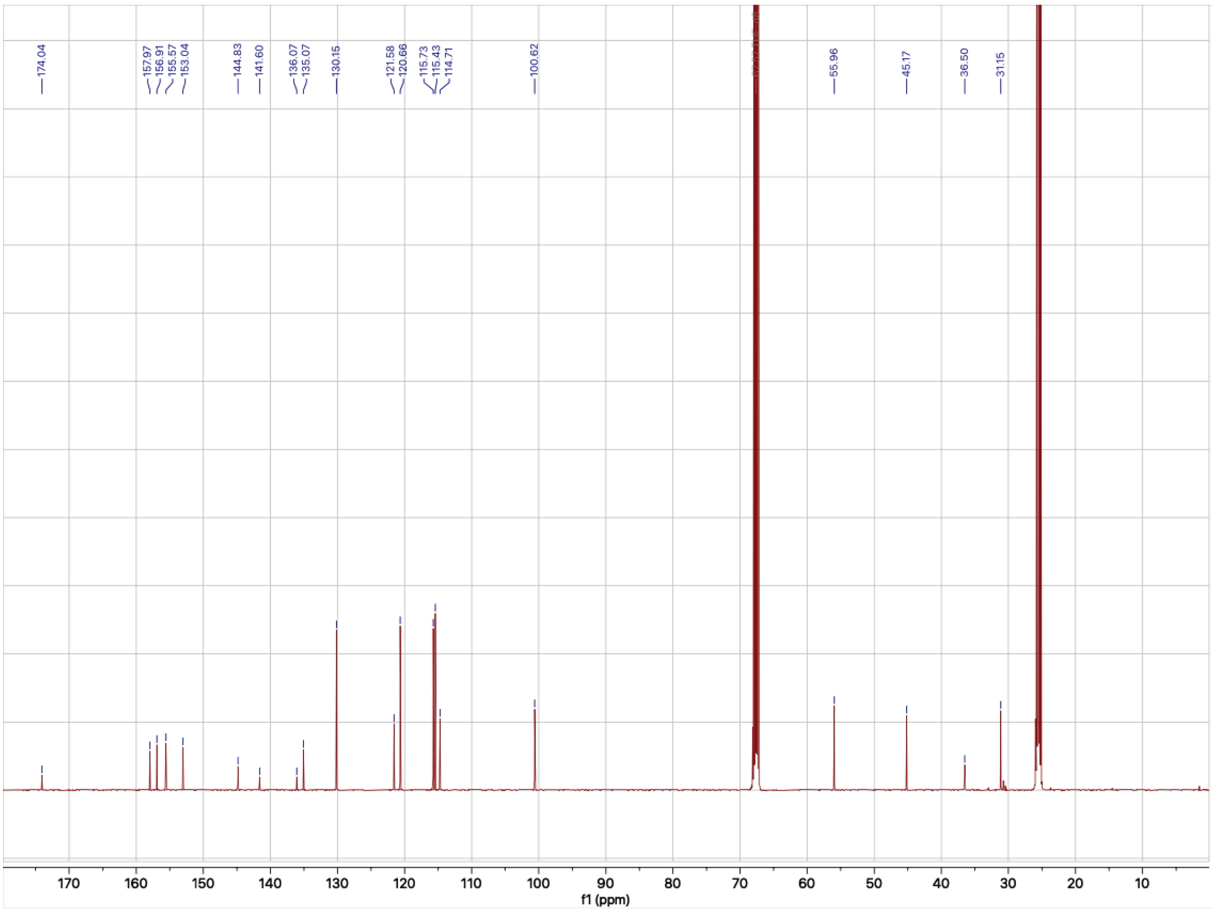

Figure S91: **2**, ^{13}C-NMR spectra.

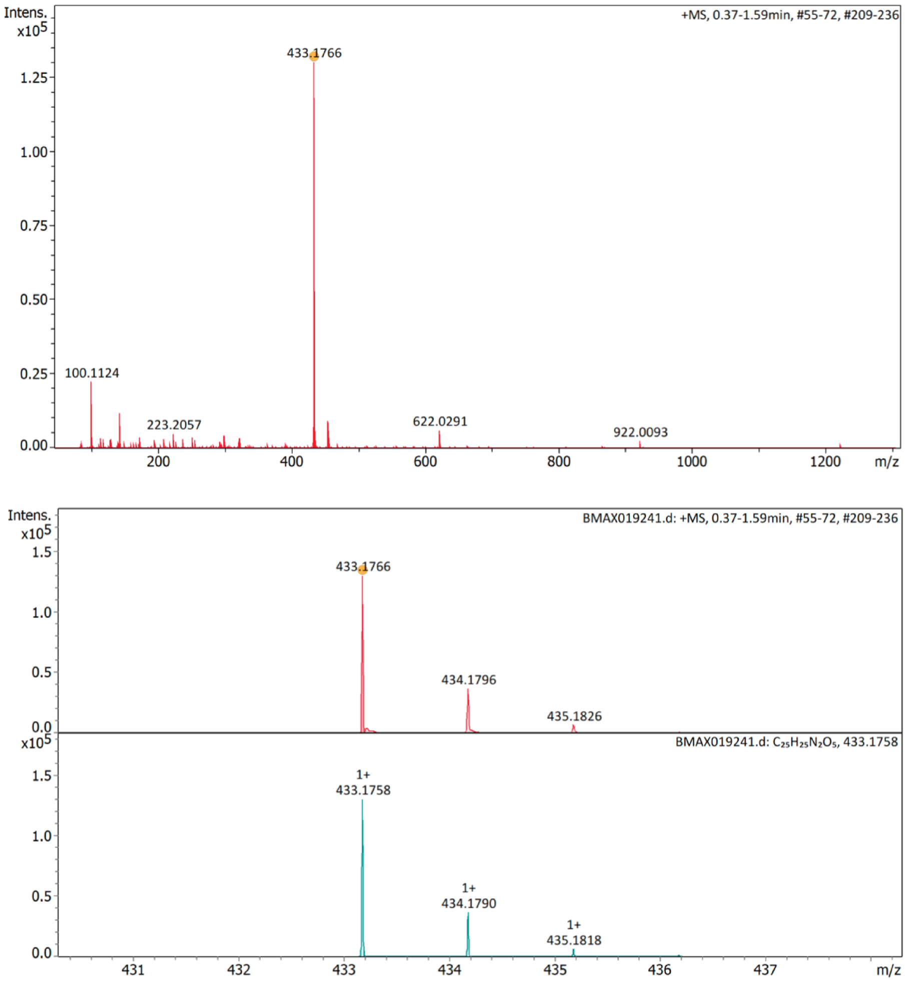

#	Ion Formula	Adduct	m/z	z	Meas. m/z	mSigma	N-Rule	err [mDa]	err [ppm]
1	C25H25N2O5	M+H	433.1758	1+	433.1766	1.2	ok	-0.8	-1.9

Figure S92: **2**, HRMS.

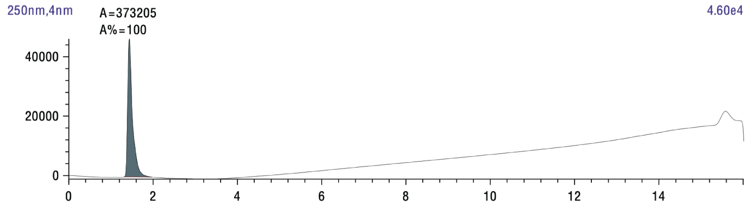

Pk#	RT	Area	Area %	Height	Height %
1	1.435	373'205	100.000	46'469	100.000
**Totals:**		**373'205**	**100.000**	**46'469**	**100.000**

Figure S93: **2**, HPLC.

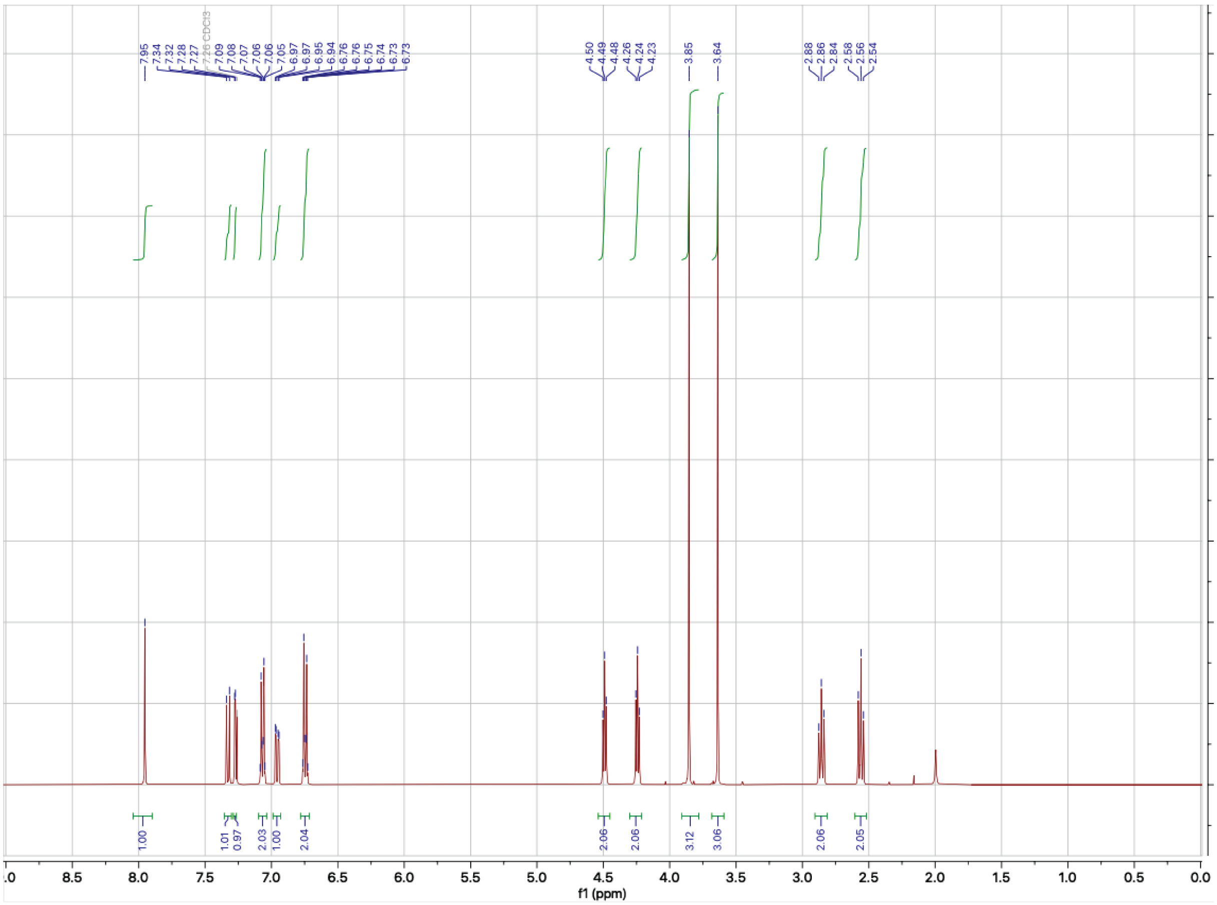

Figure S94: **32**, ^{1}H-NMR spectra.

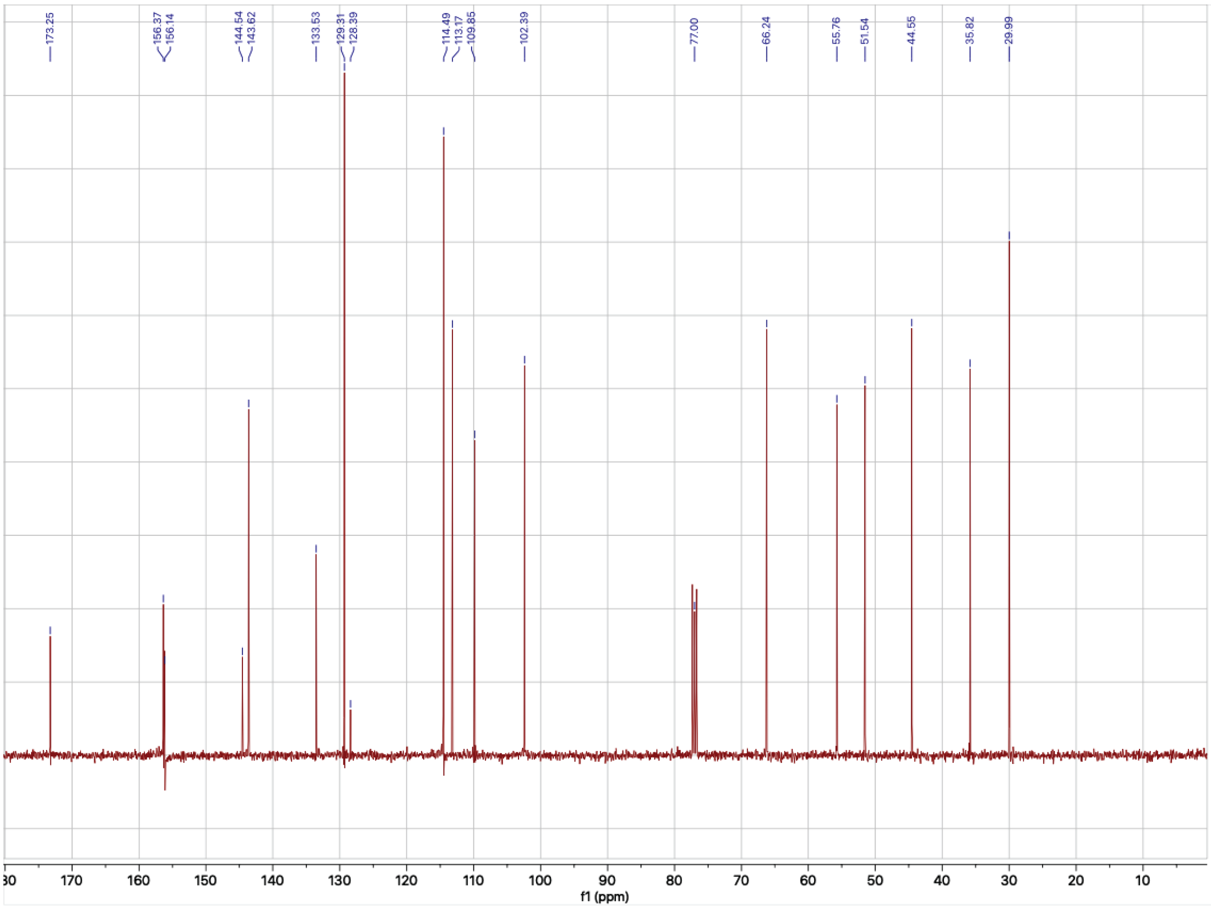

Figure S95: **2**, ^{13}C-NMR spectra.

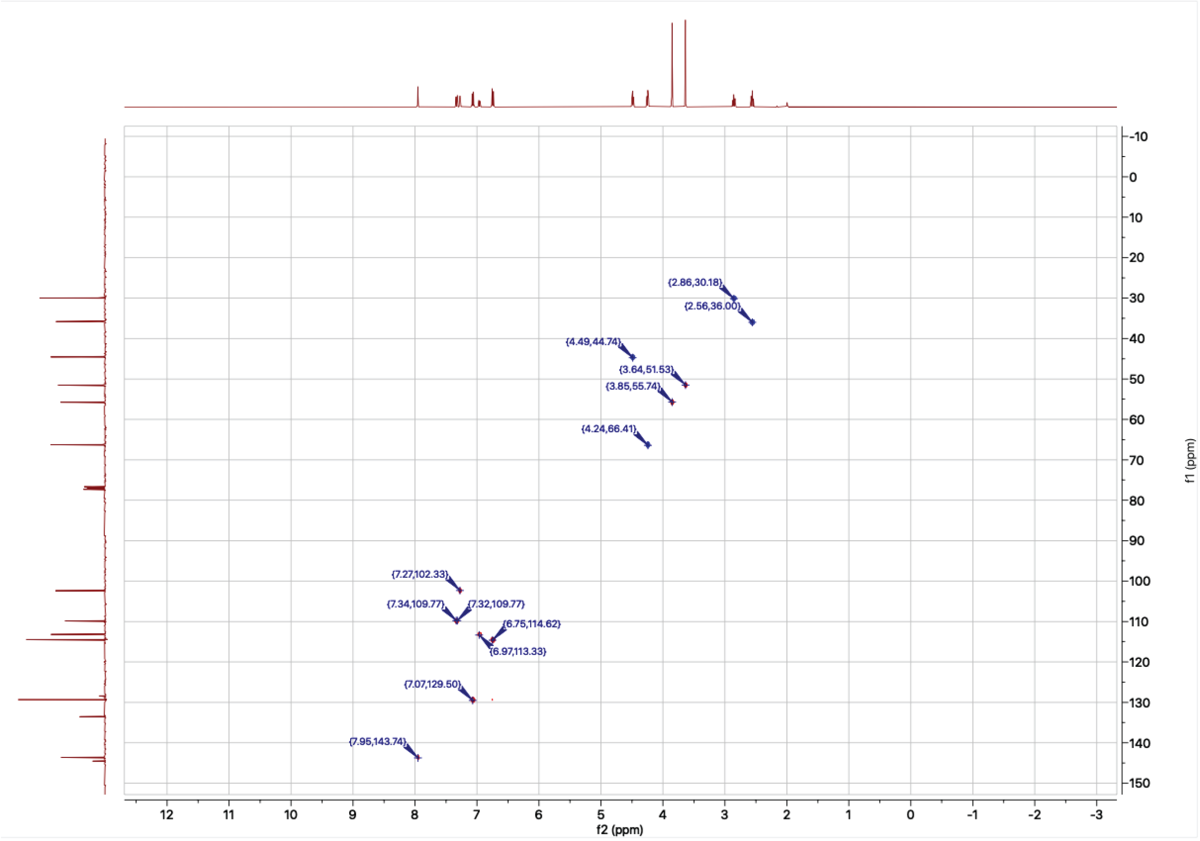

Figure S96: **32**, HSQC-NMR spectra.

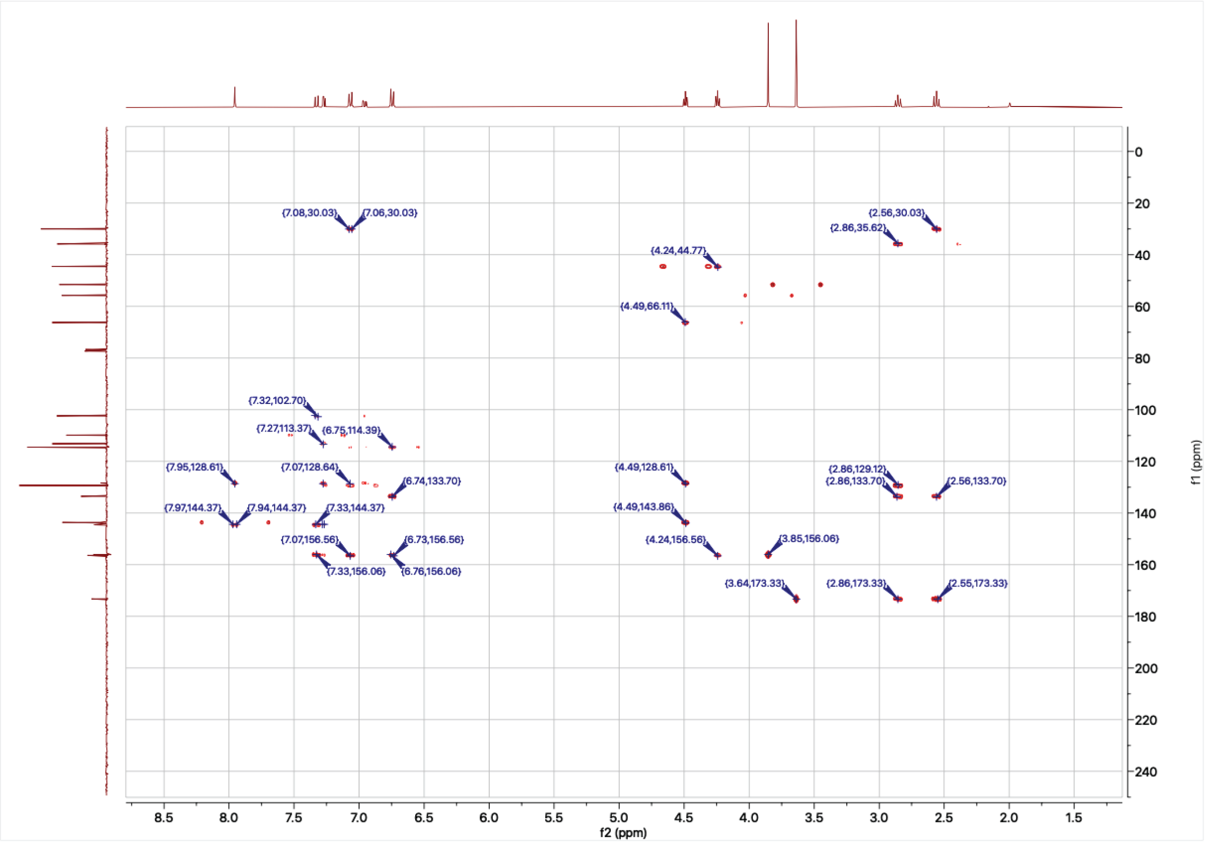

Figure S97: **32**, HMBC-NMR spectra.

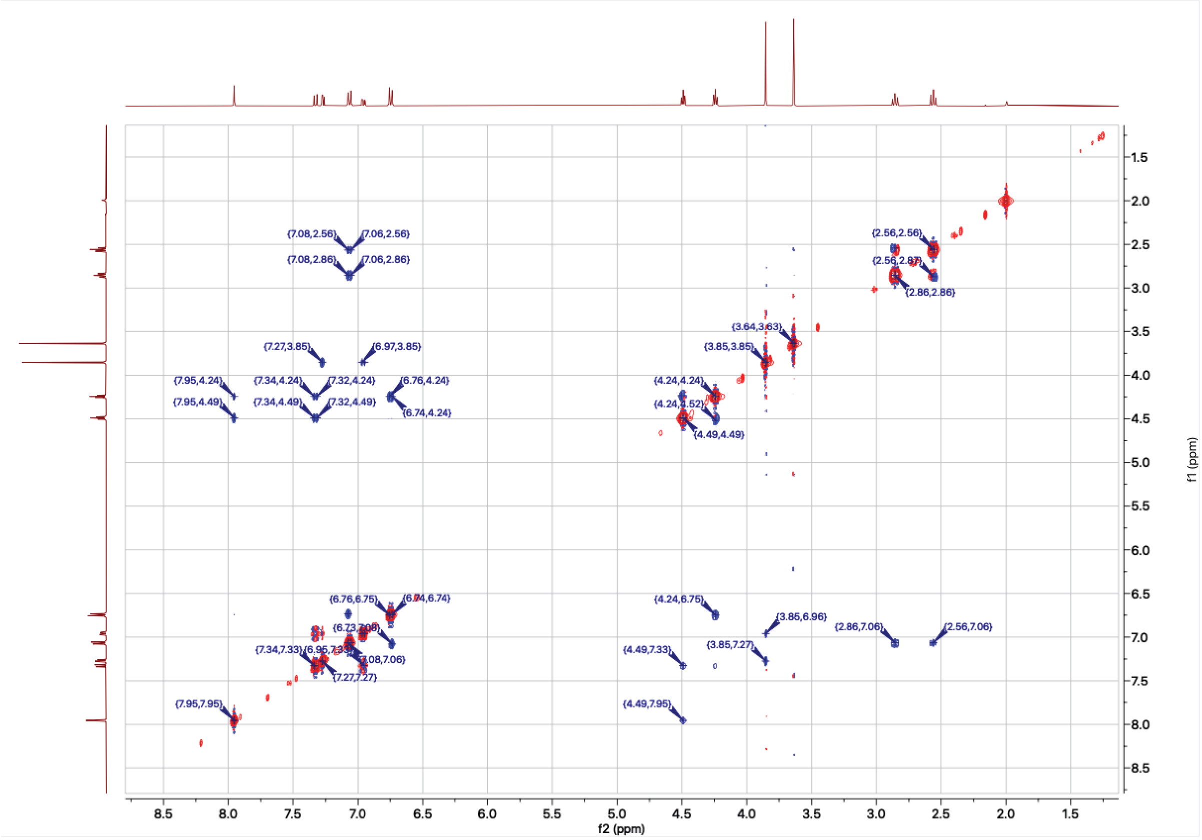

Figure S98: **32**, NOESY-NMR spectra.

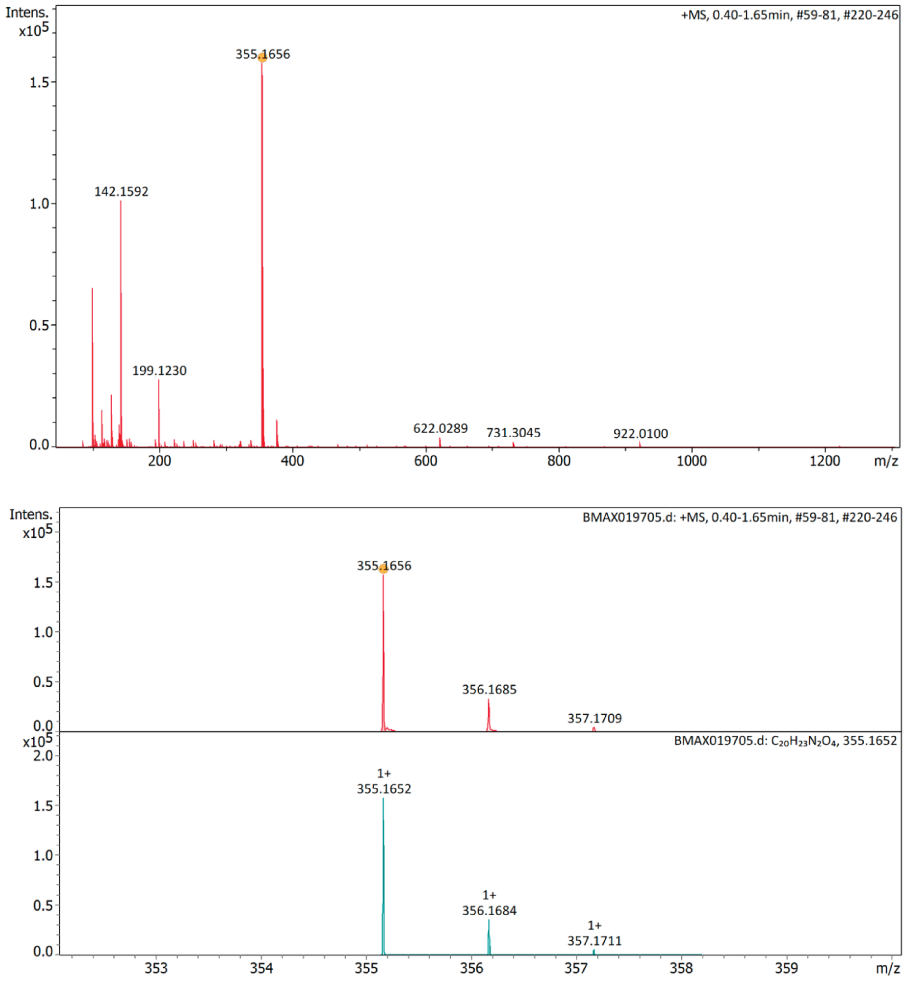

#	Ion Formula	Adduct	m/z	z	Meas. m/z	mSigma	N-Rule	err [mDa]	err [ppm]
1	C20H23N2O4	M+H	355.1652	1+	355.1656	9.3	ok	-0.4	-1.1

Figure S99: **32**, HRMS.

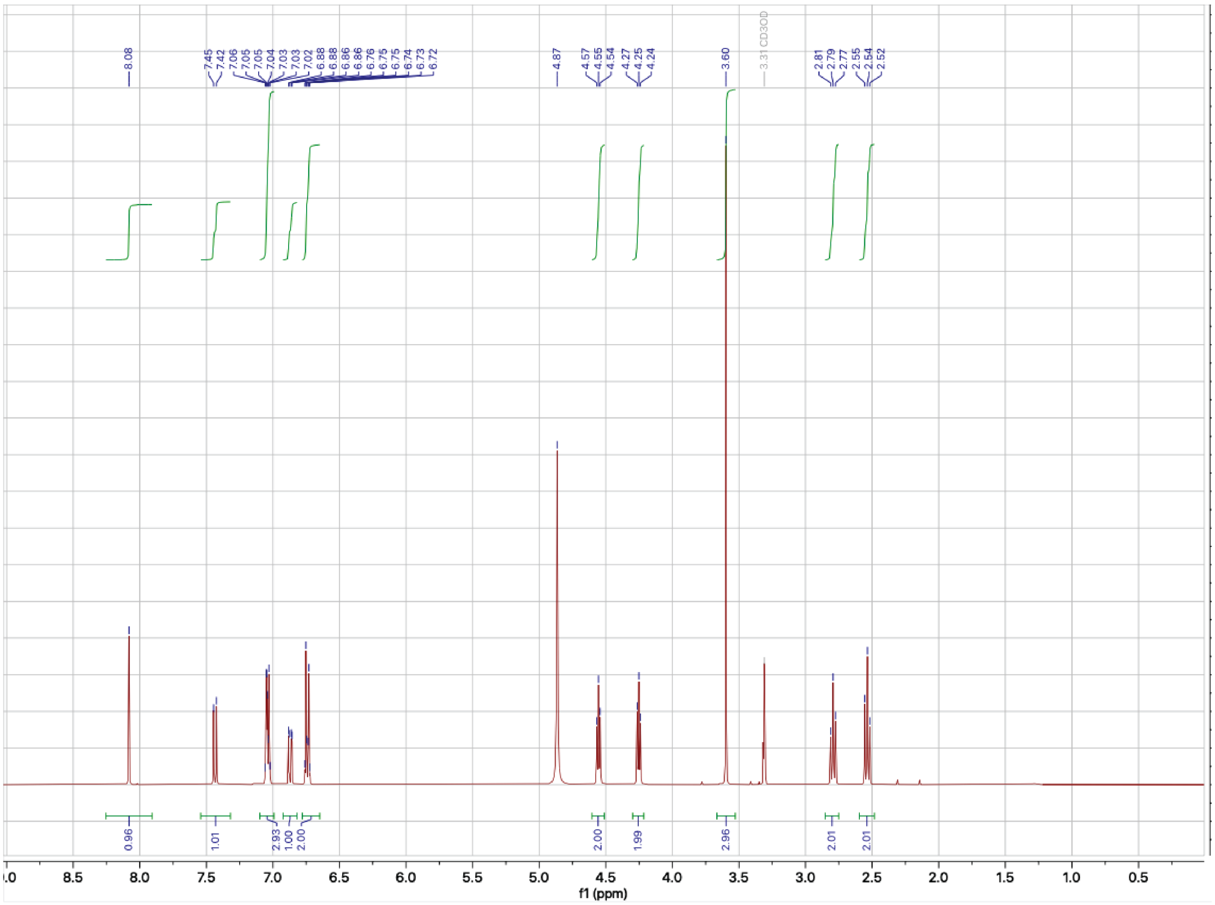

Figure S100: **36**, ^{1}H-NMR spectra.

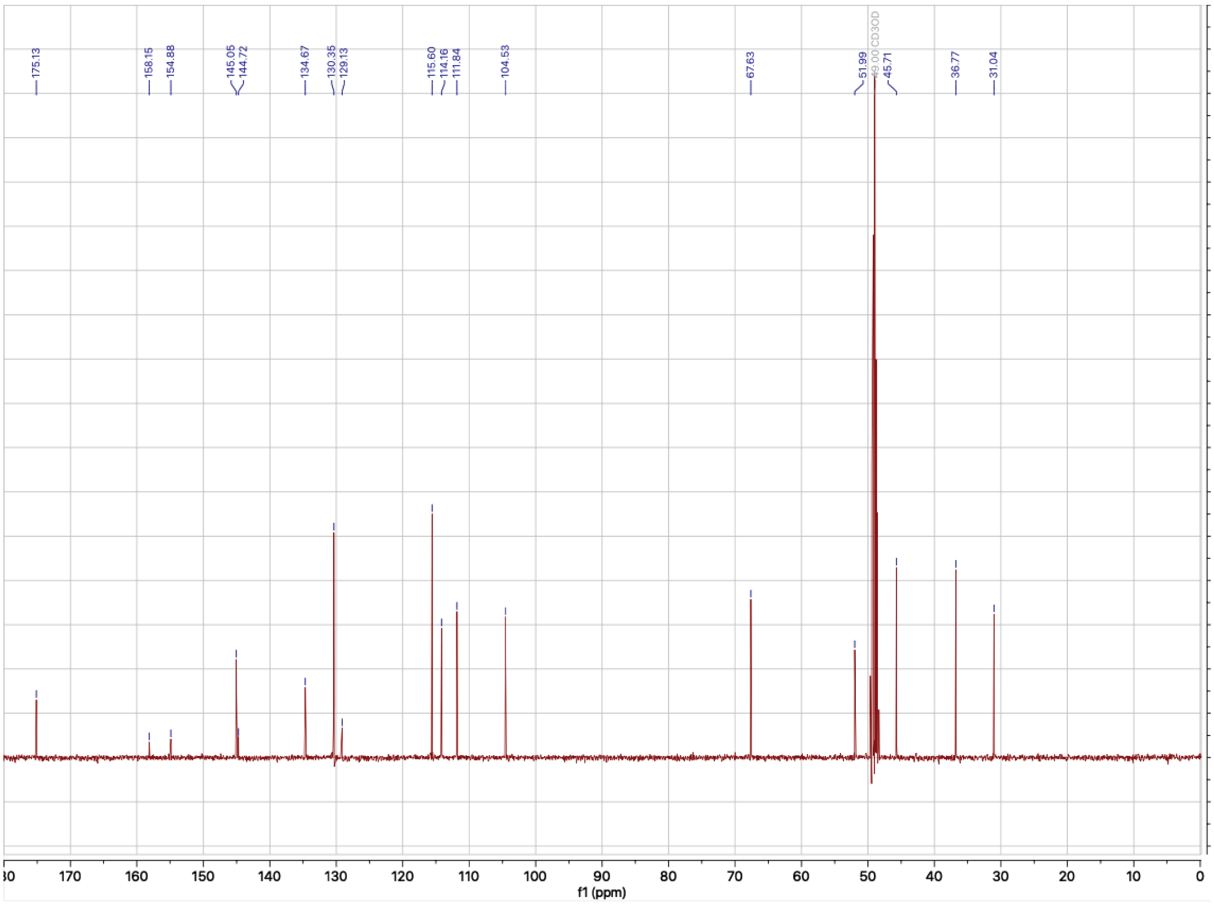

Figure S101: **36**, ^{13}C-NMR spectra.

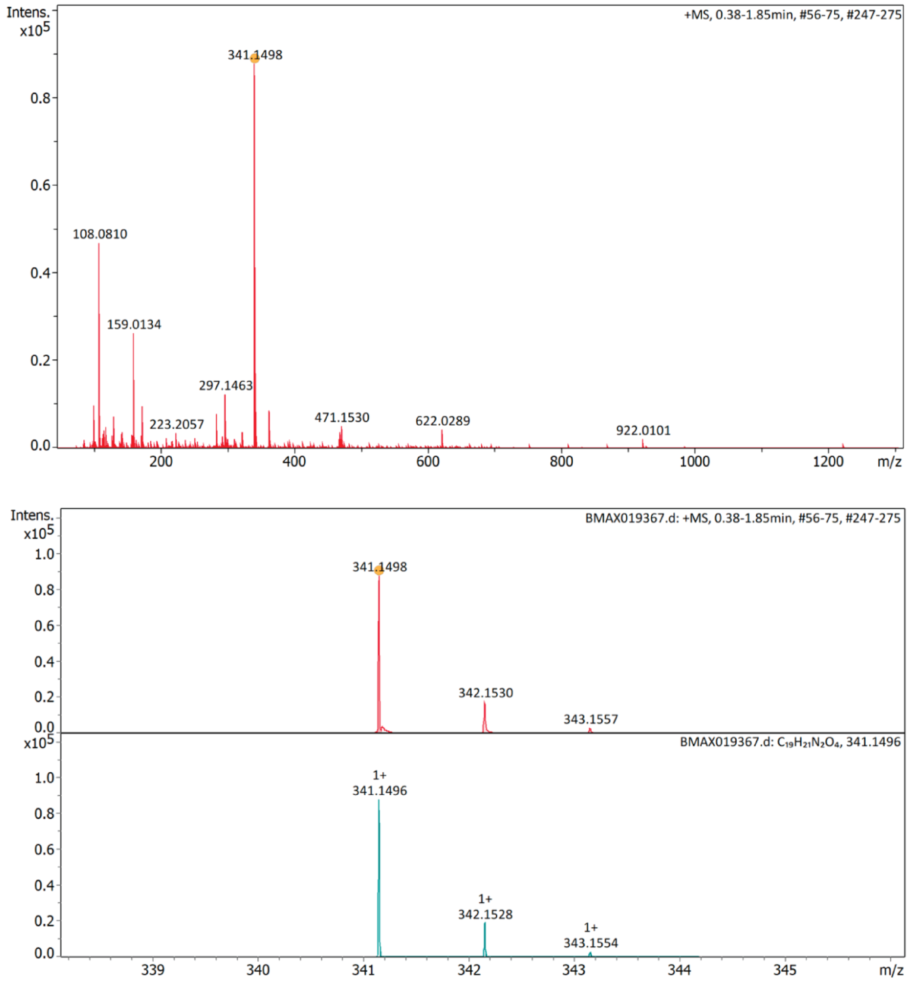

#	Ion Formula	Adduct	m/z	z	Meas. m/z	mSigma	N-Rule	err [mDa]	err [ppm]
1	C19H21N2O4	M+H	341.1496	1+	341.1498	9.6	ok	-0.2	-0.7

Figure S102: **36**, HRMS.

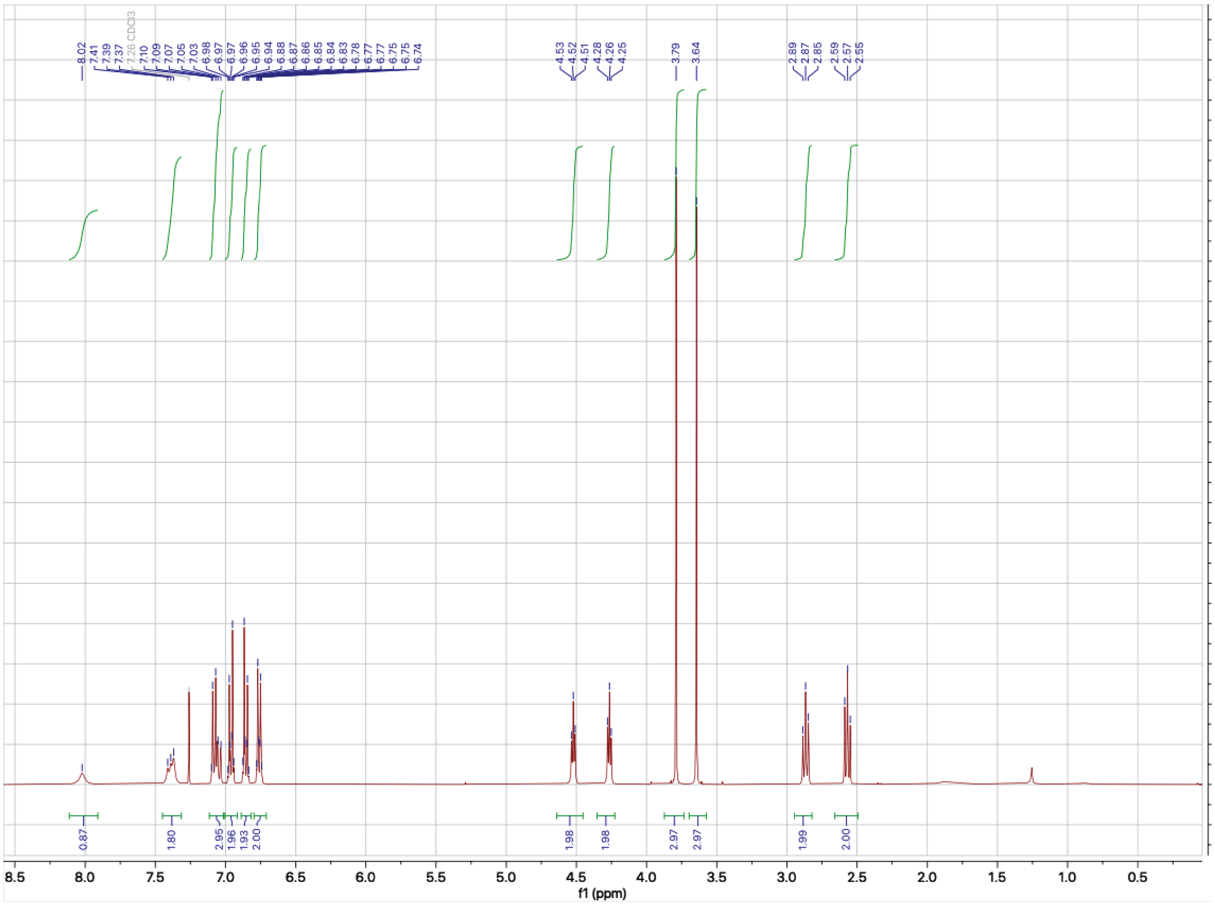

Figure S103: **38**, ^{1}H-NMR spectra.

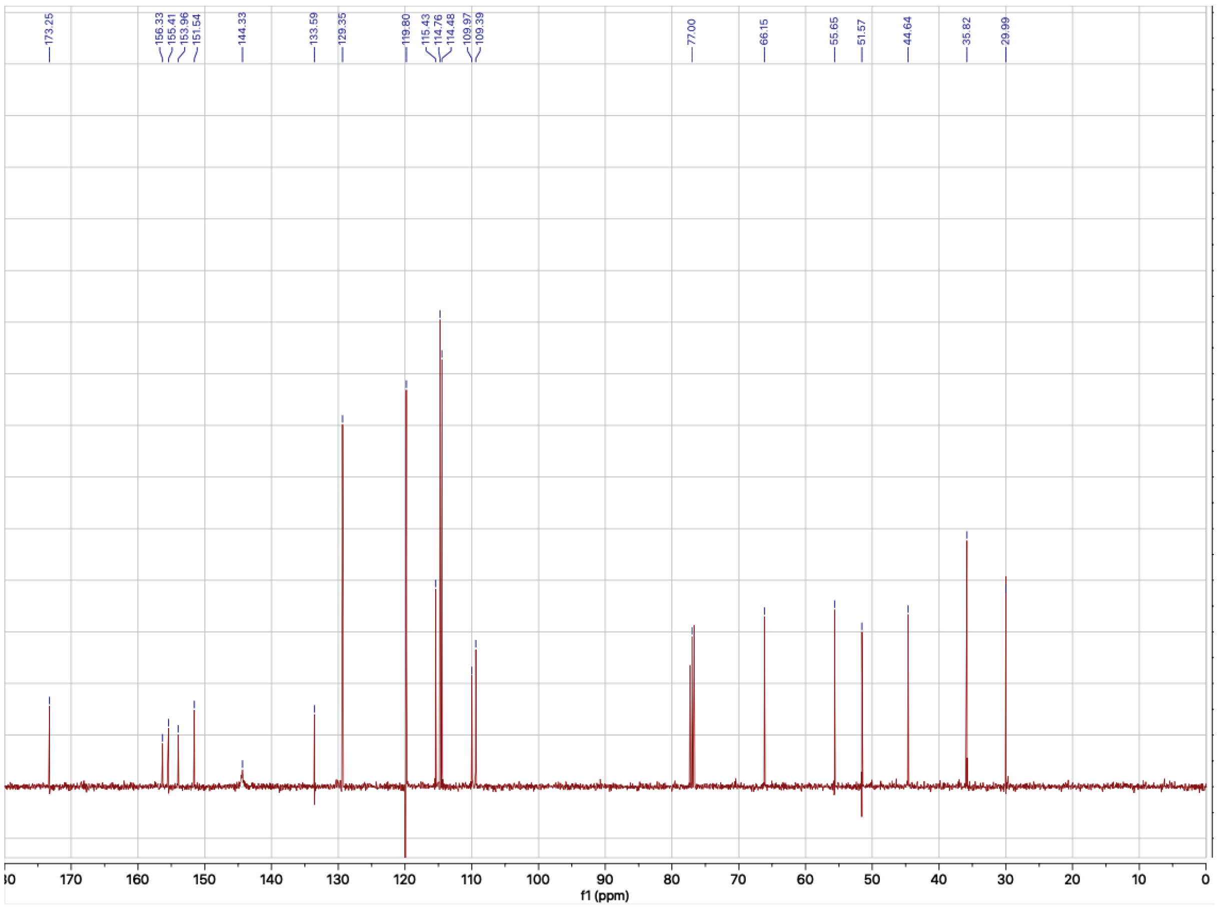

Figure S104: **38**, ^{13}C-NMR spectra.

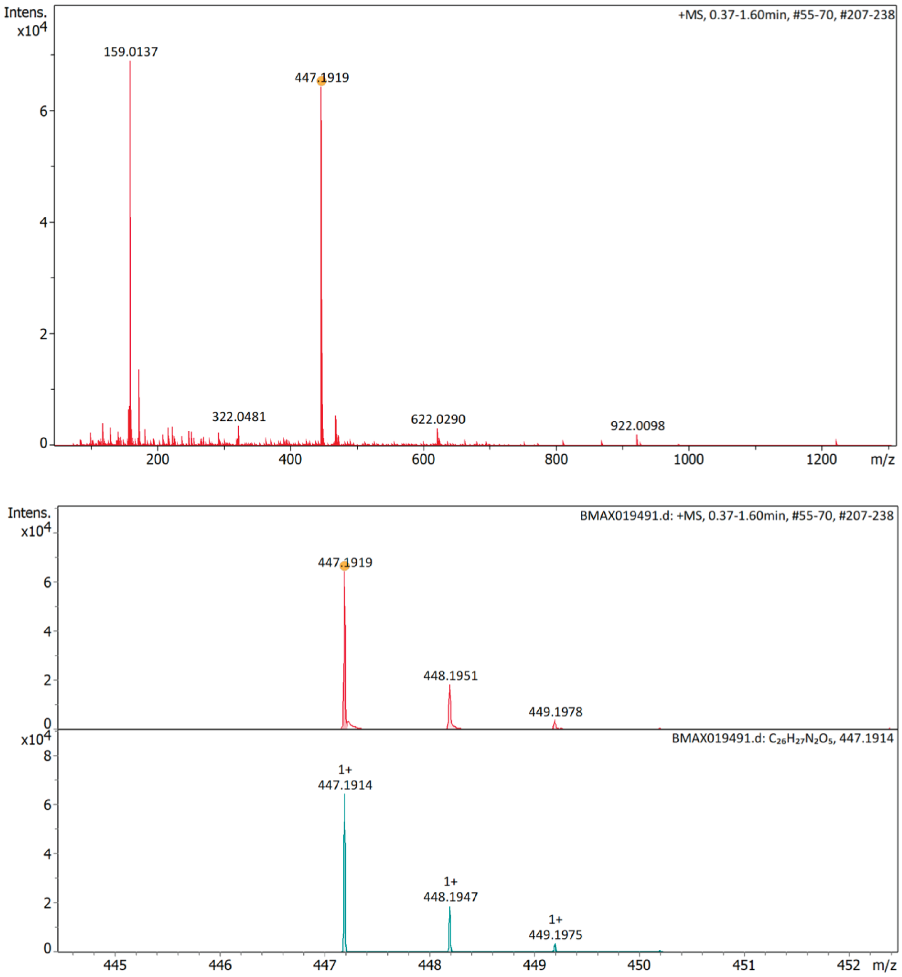

#	Ion Formula	Adduct	m/z	z	Meas. m/z	mSigma	N-Rule	err [mDa]	err [ppm]
1	C26H27N2O5	M+H	447.1914	1+	447.1919	4.7	ok	-0.5	-1.0

Figure S105: **38**, HRMS.

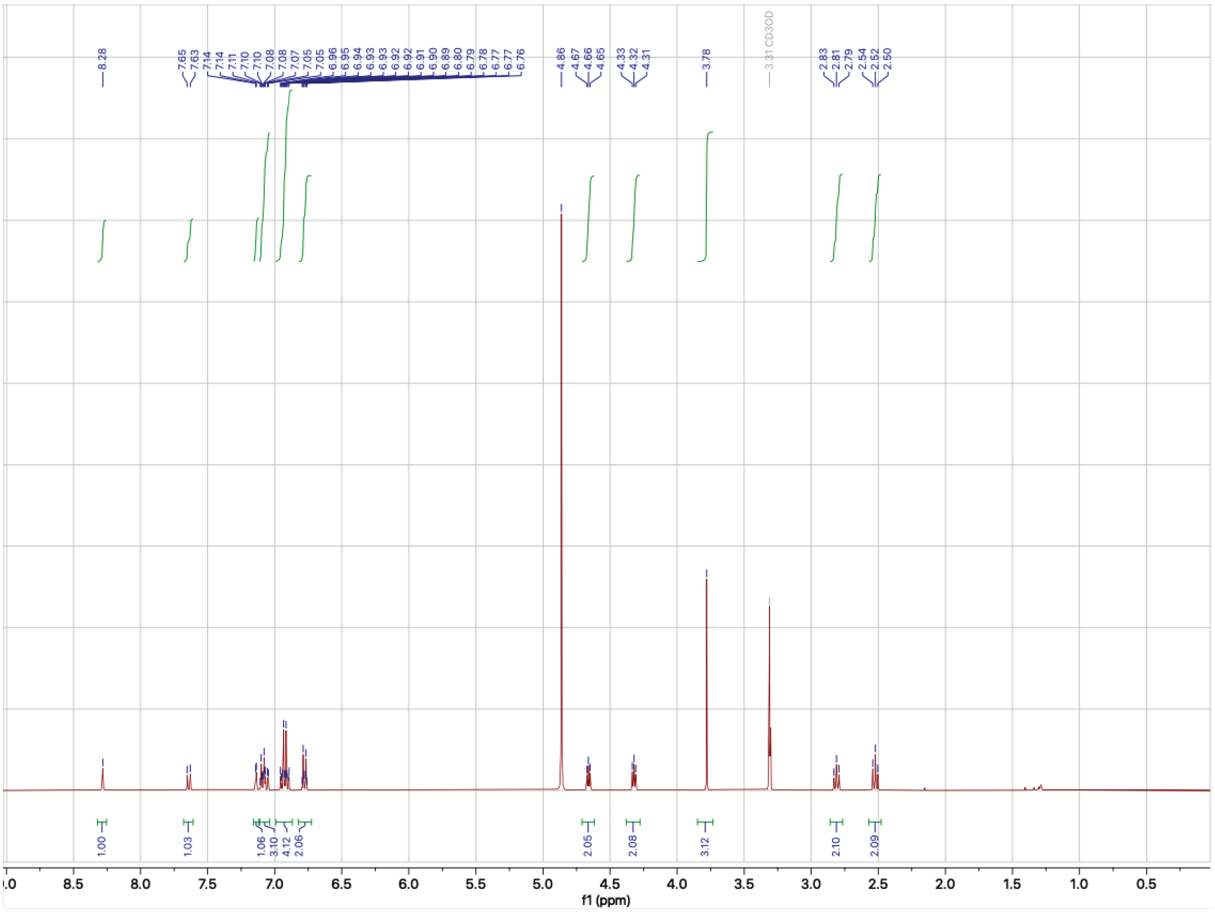

Figure S106: **3**, ^{1}H-NMR spectra.

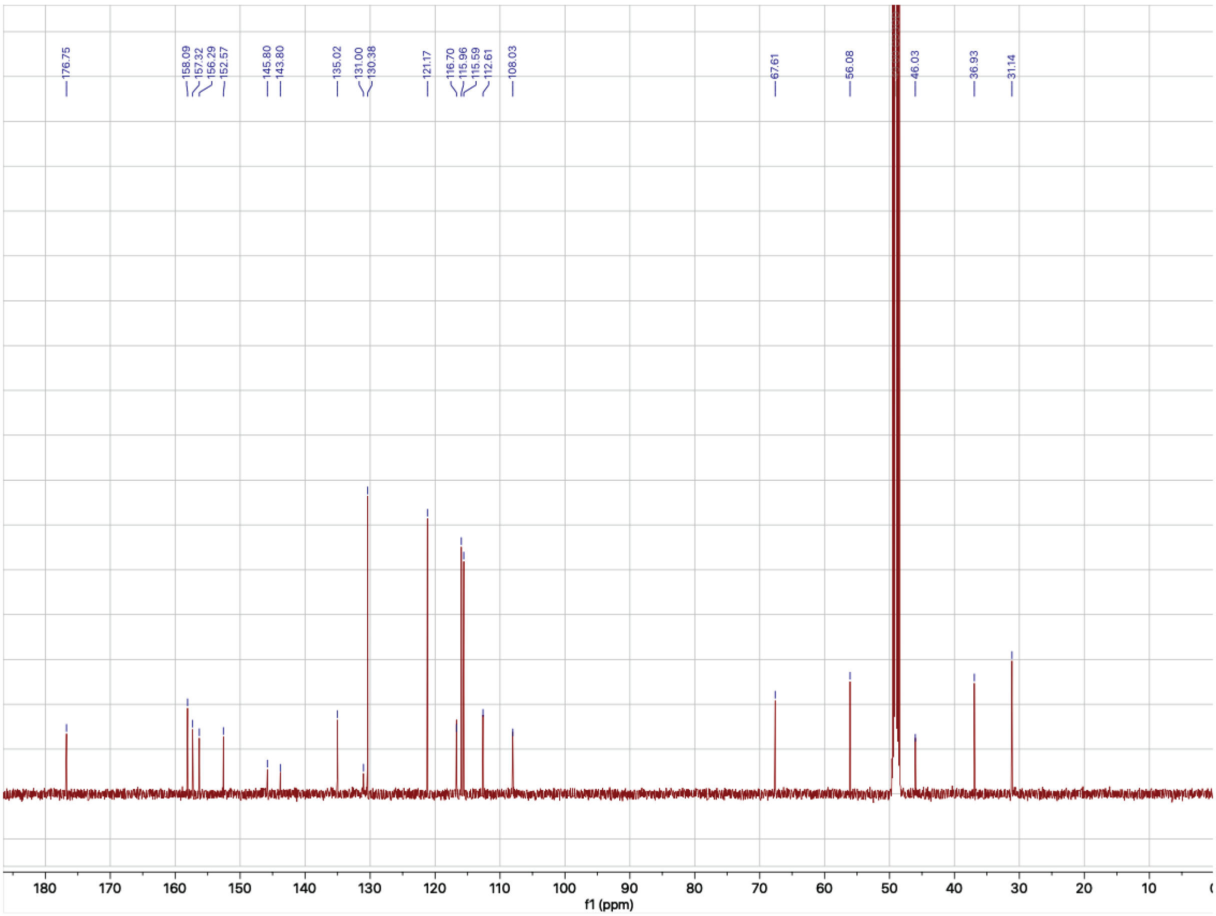

Figure S107: **3**, ^{13}C-NMR spectra.

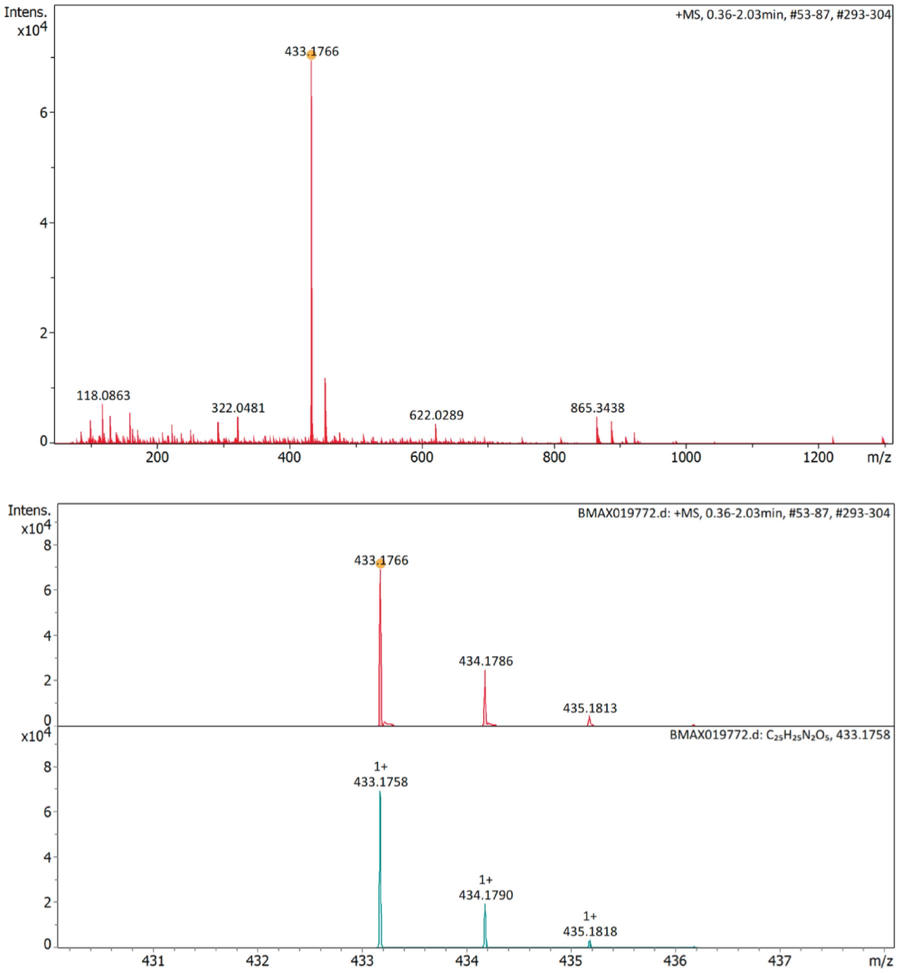

#	Ion Formula	Adduct	m/z	z	Meas. m/z	mSigma	N-Rule	err [mDa]	err [ppm]
1	C25H25N2O5	M+H	433.1758	1+	433.1766	37.5	ok	-0.8	-1.9

Figure S108: **3**, HRMS.

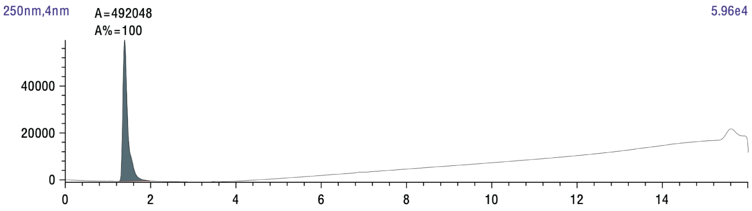

Pk#	RT	Area	Area %	Height	Height %
1	1.390	492'048	100.000	60'183	100.000
**Totals:**		**492'048**	**100.000**	**60'183**	**100.000**

Figure S109: **3**, HPLC.

References

1. Fey, M. & Lenssen, J. E. *Fast Graph Representation Learning with PyTorch Geometric* in *International Conference on Learning Representations (ICLR) Workshop on Representation Learning on Graphs and Manifolds* (2019).
2. Paszke, A. *et al.* Pytorch: An imperative style, high-performance deep learning library. *Adv. Neural. Inf. Process. Syst.* **32,** 8026–8037 (2019).
3. Kingma, D. P. & Ba, J. Adam: A method for stochastic optimization. *arXiv preprint at arXiv:1412.6980* (2014).
4. Grisoni, F., Moret, M., Lingwood, R. & Schneider, G. Bidirectional Molecule Generation with Recurrent Neural Networks. *J. Chem. Inf. Model.* **60,** 1175–1183 (2020).
5. Blaschke, T. *et al.* REINVENT 2.0: an AI tool for de novo drug design. *J. Chem. Inf. Model.* **60,** 5918–5922 (2020).
6. Wang, M. *et al.* Deep learning approaches for de novo drug design: An overview. *Curr. Opin. Struct. Biol.* **72,** 135–144 (2022).
7. Mendez, D. *et al.* ChEMBL: Towards direct deposition of bioassay data. *Nucleic Acids Res.* **47,** D930–D940 (2019).
8. Müller, K.-R., Finke, M., Murata, N., Schulten, K. & Amari, S. A numerical study on learning curves in stochastic multilayer feedforward networks. *Neural Comput.* **8,** 1085–1106 (1996).
9. Chen, T. & Guestrin, C. Xgboost: A scalable tree boosting system. *International Conference on Knowledge Discovery and Data Mining,* 785–794 (2016).
10. Pedregosa, F. *et al.* Scikit-learn: Machine learning in Python. *J. Mach. Learn. Res.* **12,** 2825–2830 (2011).
11. Thakkar, A., Chadimová, V., Bjerrum, E. J., Engkvist, O. & Reymond, J.-L. Retrosynthetic accessibility score (RAscore)–rapid machine learned synthesizability classification from AI driven retrosynthetic planning. *Chem. Sci.* **12,** 3339–3349 (2021).
12. Genheden, S. *et al.* AiZynthFinder: a fast, robust and flexible open-source software for retrosynthetic planning. *J. Cheminformatics* **12** (2020).
13. Zhavoronkov, A. *et al.* Deep learning enables rapid identification of potent DDR1 kinase inhibitors. *Nat. Biotechnol.* **37,** 1038–1040 (2019).
14. Walters, W. P. & Murcko, M. Assessing the impact of generative AI on medicinal chemistry. *Nat. Biotechnol.* **38,** 143–145 (2020).
15. Bemis, G. W. & Murcko, M. A. The properties of known drugs. 1. Molecular frameworks. *J. Med. Chem.* **39,** 2887–2893 (1996).
16. Reetz, M. T., Carballeira, J. D. & Vogel, A. Iterative saturation mutagenesis on the basis of B factors as a strategy for increasing protein thermostability. *Angew. Chem. Int. Ed.* **118,** 7909–7915 (2006).
17. Radivojac, P. *et al.* Protein flexibility and intrinsic disorder. *Protein Sci.* **13,** 71–80 (2004).
18. Oracle Corporation. *MySQL. Accessed September 2020.* `https://dev.mysql.com/`.
19. Bento, A. P. *et al.* An open source chemical structure curation pipeline using RDKit. *J. Cheminformatics* **12** (2020).
20. Wang, R., Fang, X., Lu, Y. & Wang, S. The PDBbind database: Collection of binding affinities for protein-ligand complexes with known three-dimensional structures. *J. Med. Chem.* **47,** 2977–2980 (2004).
21. Liu, Z. *et al.* PDB-wide collection of binding data: current status of the PDBbind database. *Bioinformatics* **31,** 405–412 (2015).
22. Consortium, U. UniProt: a hub for protein information. *Nucleic Acids Res.* **43,** D204–D212 (2015).
23. Burggraaff, L. *et al.* Annotation of Allosteric Compounds to Enhance Bioactivity Modeling for Class A GPCRs. *J. Chem. Inf. Model.* **60,** 4664–4672 (2020).
24. Weininger, D. SMILES, a chemical language and information system. 1. Introduction to methodology and encoding rules. *J. Chem. Inf. Comput. Sci.* **28,** 31–36 (1988).
25. Krenn, M., Häse, F., Nigam, A., Friederich, P. & Aspuru-Guzik, A. Self-Referencing Embedded Strings (SELFIES): A 100% robust molecular string representation. *Mach. Learn.: Sci. Technol.* **1,** 045024 (2020).
26. Moret, M., Friedrich, L., Grisoni, F., Merk, D. & Schneider, G. Generative molecular design in low data regimes. *Nat. Mach. Intell.* **2,** 171–180 (2020).

27. Bénardeau, A. *et al.* Aleglitazar, a new, potent, and balanced dual PPARα/γ agonist for the treatment of type II diabetes. *Bioorg. Med. Chem. Lett.* **19,** 2468–2473 (2009).

28. M. Nakata, T. Maeda, T. Shimazaki, M. Hashimoto. *The PubChemQC Project* `http://pubchemqc.riken.jp/` Accessed Sept. 2020.

29. Madeira, F. *et al.* Search and sequence analysis tools services from EMBL-EBI in 2022. *Nuc. Ac. Res.* **50,** W276–W279 (2022).

30. Willems, S. *et al.* Endogenous vitamin E metabolites mediate allosteric PPARγ activation with unprecedented co-regulatory interactions. *Cell Chem. Biol.* **28,** 1489–1500 (2021).

31. Winter, G. *et al.* DIALS: implementation and evaluation of a new integration package. *Acta Crystallogr. D* **74,** 85–97 (2018).

32. Kabsch, W. xds. *Acta Crystallogr. D* **66,** 125–132 (2010).

33. Evans, P. R. & Murshudov, G. N. How good are my data and what is the resolution? *Acta Crystallogr. D* **69,** 1204–1214 (2013).

34. McCoy, A. J. *et al.* Phaser crystallographic software. *J. Appl. Crystallogr.* **40,** 658–674 (2007).

35. Murshudov, G. N. *et al.* REFMAC5 for the refinement of macromolecular crystal structures. *Acta Crystallogr. D* **67,** 355–367 (2011).

36. Emsley, P., Lohkamp, B., Scott, W. G. & Cowtan, K. Features and development of Coot. *Acta Crystallogr. D* **66,** 486–501 (2010).

37. Long, F. *et al.* AceDRG: a stereochemical description generator for ligands. *Acta Crystallogr. D* **73,** 112–122 (2017).

38. Schrodinger, L. The PyMOL molecular graphics system. *Version* **1,** 8 (2015).

39. Kansy, M., Senner, F. & Gubernator, K. Physicochemical high throughput screening: parallel artificial membrane permeation assay in the description of passive absorption processes. *Journal of medicinal chemistry* **41,** 1007–1010 (1998).

40. Haider, A. *et al.* Structure–activity relationship studies of pyridine-based ligands and identification of a fluorinated derivative for positron emission tomography imaging of cannabinoid type 2 receptors. *Journal of medicinal chemistry* **62,** 11165–11181 (2019).

41. Haider, A. *et al.* Identification and preclinical development of a 2, 5, 6-trisubstituted fluorinated pyridine derivative as a radioligand for the positron emission tomography imaging of cannabinoid type 2 receptors. *Journal of Medicinal Chemistry* **63,** 10287–10306 (2020).

42. Deehan, R. *et al.* Comparative transcriptional network modeling of three PPAR-α/γ co-agonists reveals distinct metabolic gene signatures in primary human hepatocytes. *PLoS One* **7,** e35012 (2012).

43. Soethoudt, M. *et al.* Cannabinoid CB2 receptor ligand profiling reveals biased signalling and off-target activity. *Nat. Communs.* **8,** 13958 (2017).

44. Barau, C., Ghaleh, B., Berdeaux, A. & Morin, D. Cytochrome P450 and myocardial ischemia: potential pharmacological implication for cardioprotection. *Fundamental & clinical pharmacology* **29,** 1–9 (2015).

45. Tamaoki, T. *et al.* Staurosporine, a potent inhibitor of phospholipidCa++ dependent protein kinase. *Biochem. Biophys. Rep.* **135,** 397–402 (1986).

46. Ma, D. & Cai, Q. N, N-Dimethyl glycine-promoted Ullmann coupling reaction of phenols and aryl halides. *Org. Lett.* **5,** 3799–3802 (2003).

47. Evans, D. A., Katz, J. L. & West, T. R. Synthesis of diaryl ethers through the copper-promoted arylation of phenols with arylboronic acids. An expedient synthesis of thyroxine. *Tetrahedron Lett.* **39,** 2937–2940 (1998).
